# Uncovering of natural allelic variants of key yield contributing genes by targeted resequencing in rice (*Oryza sativa* L.)

**DOI:** 10.1038/s41598-019-44708-z

**Published:** 2019-06-03

**Authors:** Lakshminarayana R. Vemireddy, Gopalakrishnamurty Kadambari, G. Eswar Reddy, Vijaya Sudhakara Rao Kola, Eswarayya Ramireddy, Venkata Ramana Rao Puram, Jyothi Badri, Suresh N. Eslavath, Swarajyalakshmi N. Bollineni, Bukya J. Naik, Sreelakshmi Chintala, Rameshbabu Pottepalem, Srividhya Akkareddy, Ranjithkumar Nagireddy, Lachagari V. B. Reddy, Reddaiah Bodanapu, Sivarama P. Lekkala, Navajeet Chakravartty, E. A. Siddiq

**Affiliations:** 1 Department of Genetics and Plant Breeding, S.V Agricultural College, Acharya NG Ranga Agricultural University (ANGRAU), Tirupati, 517502 Andhra Pradesh India; 2 Regional Agricultural Research Station, ANGRAU, Tirupati, India; 3 Biology division, Indian Institute of Science Education and Research Tirupati (IISER Tirupati), Tirupati, India; 4 RegionalAgricultural Research Station, ANGRAU, Maruteru, India; 5 ICAR-Indian Institute of Rice Research (ICAR-IIRR), Hyderabad, India; 6 Agricultural Research Station, ANGRAU, Nellore, India; 7 Institute of Biotechnology, PJTSAU, Hyderabad, India; 8 AgriGenome Labs Pvt. Ltd., Hyderabad, India

**Keywords:** Genome evolution, Plant hybridization

## Abstract

In rice (*Oryza sativa* L.), during the course of domestication, numerous beneficial alleles remain untapped in the progenitor wild species and landraces. This study aims at uncovering these promising alleles of six key genes influencing the yield, such as *DEP1*, *Ghd7*, *Gn1a*, *GS*3, *qSW5* and *sd1* by targeted resequencing of the 200 rice genotypes. In all, 543 nucleotide variations including single nucleotide polymorphisms and insertion and deletion polymorphisms were identified from the targeted genes. Of them, 225 were novel alleles, which identified in the present study only and 91 were beneficial alleles that showed significant association with the yield traits. Besides, we uncovered 128 population-specific alleles with *indica* being the highest of 79 alleles. The neutrality tests revealed that pleiotropic gene, *Ghd7* and major grain size contributing gene, *GS3* showed positive and balanced selection, respectively during the domestication. Further, the haplotype analysis revealed that some of the rice genotypes found to have rare haplotypes, especially the high yielding variety, BPT1768 has showed maximum of three genes such as *Gn1a-8*, *qSW5-12* and *GS3-29*. The rice varieties with novel and beneficial alleles along with the rare haplotypes identified in the present study could be of immense value for yield improvement in the rice breeding programs.

## Introduction

Crop domestication from wild and *landraces* and subsequent improvement by breeding methods shaped the genetic constitution of the present-day modern cultivars. During crop evolution, domestication and improvement are two crucial events that led to the generation of novel alleles in modern cultivars, and at the same time, many alleles remain untapped in wild and landraces/primitive cultivars. These promising untapped alleles can be of immense value for the development of superior cultivars. Uncovering of these untapped alleles will enable the breeders to design varieties with customised traits- a viable option to feed the projected 10 billion global population by 2050^1^. The advent of innovative sequencing techniques facilitated in uncovering these hidden alleles from the available germplasm of the crops.

Rice (*Oryza sativa* L.), is a staple crop and rich source of calories for over three billion people on the planet. Having a domestication history of 9000 years, rice is endowed with a wealth of genetic diversity in the existing germplasm. Although, the origin of domestication of rice has contrasting evidences as to whether single or multiple origins, the current germplasm can be classified into five sub-groups- *indica*, *tropical japonica*, *temperate japonica/javanica*, *aromatic* and *aus*^2^. The native population’s harbour wealth of nucleotide variations for different traits and provide an excellent opportunity to uncover essential novel and favourable alleles of single nucleotide polymorphism (SNPs) and insertions and deletions (indels). Besides, the nucleotide variations allow us for the elucidation of valuable information about the evolutionary history of a gene of interest within and between species. With the recent efforts of IRRI and other Institutions, now the sequences of more than 4,000 rice accessions are publicly available in databases such as Rice Variation Map (http://ricevarmap.ncpgr.cn/v2/) and Rice SNP-Seek database (http://snp-seek.irri.org/). However, establishing an association between untapped potential alleles in the germplasm and their respective phenotypes–a prerequisite for introgression of these alleles into cultivated varieties still poses a challenge to the breeders. Transferring of these superior alleles to elite genetic backgrounds would aid in increased trait performance.

There is a pressing need to break the yield plateau and raise the yield levels of rice at least by 50% in the coming 30 years of time^3^. Moreover, this mammoth task has to be achieved with the continuous declining of land and water resources coupled with unpredictable extreme environmental changes. Rice grain yield is largely determined by three of its essential component traits *viz*., grain number, number of panicles per plant (productive tillers) and grain weight or grain size, which are governed mainly by quantitative trait loci (QTLs). As of now, more than 34 QTLs related to yield have been precisely cloned and functionally characterized^4^. Some of the major genes are – *Gn1a*^5^, *DEP1*^6^ and *Ghd7*^7^ for grain number, *GS3*^8^, *GS5*^9^ and *qSW5*^10^ for grain size and grain weight.

Till now, few attempts have been made by various research groups on allele mining of yield contributing genes such as *Gn1a*^11^, *GS3*^12^, *Ghd7*^13^, *DEP1*^14^, and *sd1*^15^ in isolation using diverse rice germplasm. For instance, Lu *et al*.^13^ identified 76 SNPs and six indels for *Ghd7* gene that governs plant height, heading date and yield. Similarly, for *Gn1a* gene, 14 alleles have been identified and phylogenetic analysis revealed migration of three main alleles, *AP3*, *AP8* and *AP9* in the cultivars from a common ancestor allele, *AP1*, in the wild rice^11^. However, the extent of the existing natural variation and its association with phenotype for many of the genes is still unclear. Comprehensive elucidation of each gene in grain yield formation facilitates raising the yield levels. Hence, it is quite worthwhile to investigate nucleotide variants of the key yield genes in a comprehensive manner as many of them act in concert to result in final yield. Earlier, Lu *et al*.^16^ identified substantial variation in four grain size controlling genes *viz*., *GW2*, *GS5*, *GS3* and *qSW5* using 127 rice varieties. Keeping this in view, the present investigation was aimed to identify allelic variants of the six yield contributing genes *viz*., *Ghd7*, *Gn1a*, *GS3*, *DEP1*, *qSW5* and *sd1* employing sequence-based allele mining strategy.

## Results and Discussion

### Phenotypic variation for yield and its component traits

The phenotypic variation for important yield attributing traits of the rice genotypes was recorded in two environments *i*.*e*., Agricultural Research Station (ARS), Nellore and Regional Agricultural Research Station (RARS), Maruteru are provided in Supplementary Table S1. We found substantial variation for many of the yield and its component traits in all the genotypes in both the locations. The economic yield is positively correlated with number of panicles, number of filled grains, spikelet number per panicle and spikelet fertility as expected since these component traits largely determine the yield while negatively correlated with the chaffy grains in both the environments (Supplementary Table S2).

### Nucleotide variations in the targeted yield genes

**Figure 1:**
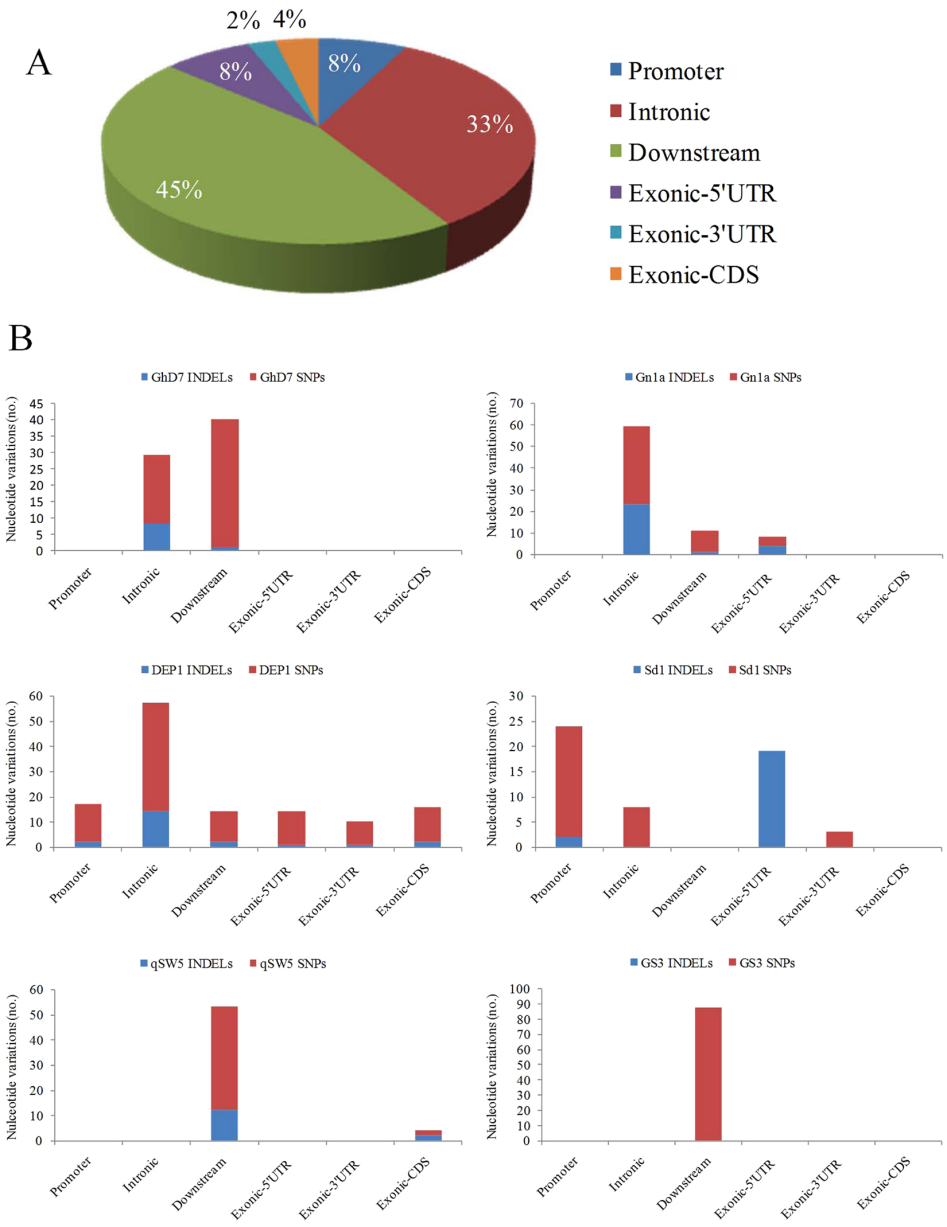
Nucleotide variations in the targeted yield genes. (**A**) Overall nucleotide variations in the targeted six genes obtained by resequencing of the 200 rice genotypes. (**B**) The gene-wise nucleotide variations including indels and SNPs in the different gene components. UTR- Untranslated region; CDS- Coding sequence; Promoter-1000bp upstream region of the gene.

In this study, we uncovered as many as 543 nucleotide variations in the targeted six genes *viz*., *GS3*, *Ghd7*, *Gn1a*, *qSW5*, *DEP1* and *sd1* by resequencing of 200 rice genotypes using next-generation sequencing (NGS)-based DNA-pooled amplicon sequencing. Majority of these variations (45%) were located in the downstream region followed by in the intronic region (33%) of the genes. Very limited number of variations were found in the promoter (8%), exonic-5′UTR (8%), exonic −3′UTR (2%) and exonic-CDS (4%) region of the genes (Fig. 1A,B). Of total alleles, 3.7% of missense mutations or non-synonymous SNPs (nsSNPs), 23.5% of population-specific alleles and 13.8% of novel alleles were found (Supplementary Table S3). Of six genes, *DEP1* found to have a maximum number of total nucleotide variations (128), especially SNPs (106), whereas, *Gn1a* recorded a maximum number of indels (28). The gene-wise findings from the nucleotide variation, association and haplotype analysis are provided hereunder.

**Figure 2:**
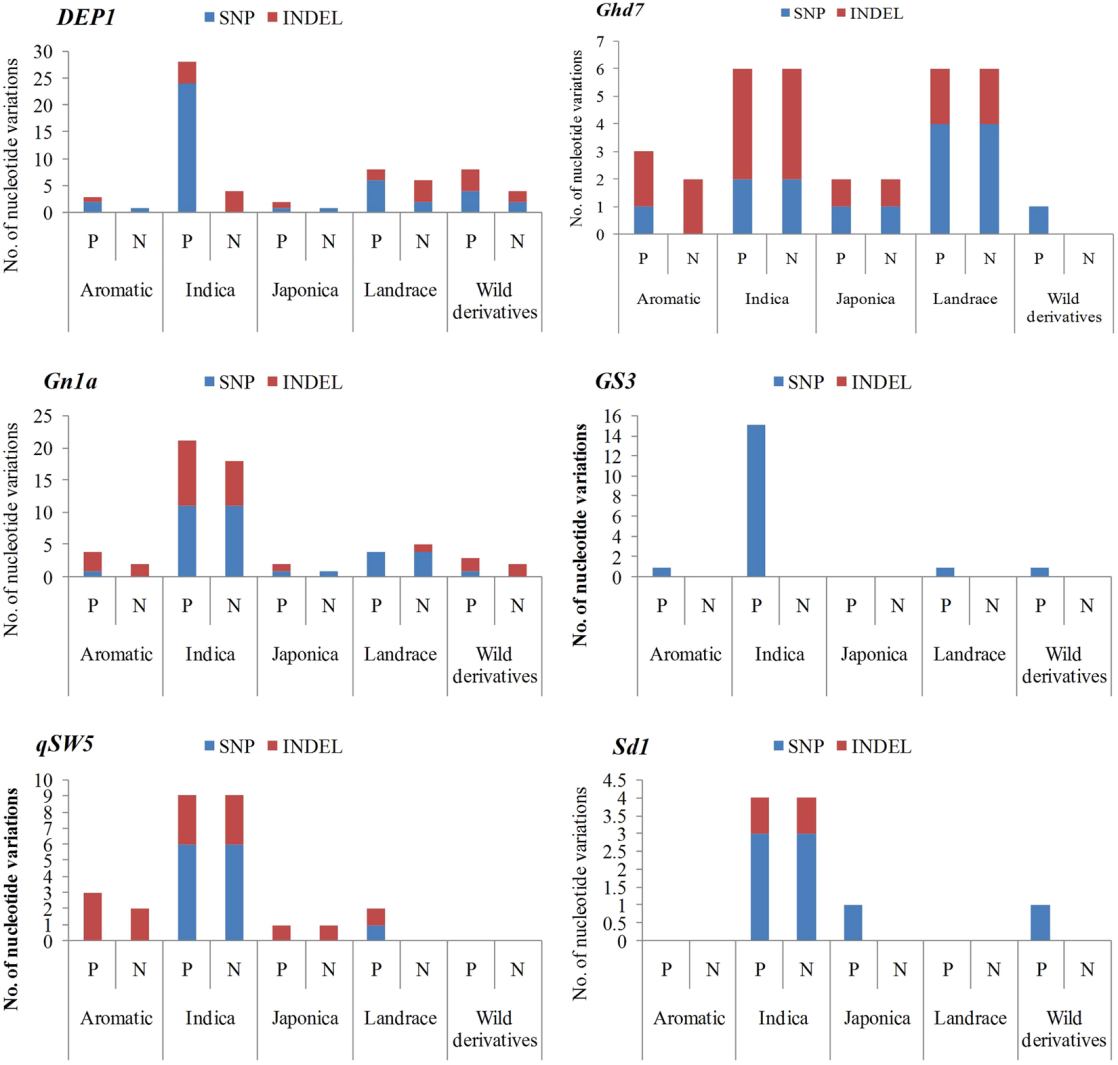
The gene-wise population-specific (P) and novel (N) nucleotide variations in all five rice groups. The nucleotide variations include both indels and SNPs in the genic region along with the promoter region. N - The novel alleles found within the total population-specific (P) alleles. For more details of the population-specific and novel alleles refer Materials and Methods section.

#### Ghd7 (Grain number, Plant height, and Heading date7)

The pleiotropic gene, *Ghd7* controls three major agronomic traits such as plant height, heading date and grain number and functionally encodes CCT domain protein^7^. The resequencing of *Ghd7* locus (3918 bp) in all genotypes revealed 69 nucleotide variations including 60 SNPs and 9 indels. Of which, 29 variations were found in the intronic region while 40 were in the downstream region of the gene (Fig. 1B). Further, of 69 alleles, 44 were found to be common alleles to RiceVarMap, and the remaining 25 were novel alleles identified in the present investigation. *Indica* and *landraces* consisting of six group–specific alleles each and notably all are novel alleles. In the remaining groups, *aromatic* and *japonica* groups comprised of three and two-group specific alleles and of which two and one are novel alleles, respectively (Supplementary Table S3; Fig. 2). Interestingly, all the indels identified in *Ghd7* gene were found to be novel. Previously, Lu *et al*.^13^ identified 76 SNPs and 6 indels using 104 rice accessions in *Ghd7* gene.

The nucleotide diversity analysis of the *Ghd7* gene revealed that the Pi values were higher in *wild derivatives* than the other group genotypes. Neutrality analysis of the *Ghd7* locus showed significant negative values only in the *indica* group, indicating a positive selection and deviation from the model of neutrality. Contrast to this, Lu *et al*.^13^ found significant positive Tajima’s D parameter in the entire genomic region of *Ghd7* and suggested a balancing selection in the locus during rice evolution and breeding. The *japonica* group showed significant positive values in both Fu and Li’s F* and D* tests. While all five groups showed significant positive values in Fu and Li’s D* test, in Fu and Li’s F* test, they showed only positive non-significant values (Table 1).

**Table 1:** Nucleotide diversity and neutrality tests of the targeted genes.

Gene	Group	Number of polymorphic (segregating) sites, S	Fu and Li’s D*	Fu and Li’s F*	Nucleotide diversity, Pi	Tajima’s D
*Ghd7*	*Aromatic*	67	1.868**	1.023	0.127	−1.160
	*Indica*	72	2.611**	0.649	0.060	−1.832*
	*Japonica*	19	1.554**	1.833**	0.068	1.684
	*Landrace*	29	1.812**	0.842	0.099	−1.323
	*Wild derivatives*	69	1.604**	1.392	0.209	0.176
*DEP1*	*Aromatic*	69	1.872**	1.896**	0.165	1.064
	*Indica*	77	2.635**	1.843*	0.136	0.286
	*Japonica*	63	1.694**	1.622*	0.121	0.670
	*Landrace*	75	2.050**	1.580	0.160	0.071
	*Wild derivatives*	58	1.665**	1.620*	0.122	0.734
*Gn1a*	*Aromatic*	52	1.563**	1.518	0.191	0.720
	*Indica*	11	1.367	1.593	0.231	1.259
	*Japonica*	41	1.658**	1.926**	0.231	1.690
	*Landrace*	27	1.789**	1.897*	0.279	1.241
	*Wild derivatives*	32	1.360*	1.086	0.130	−0.156
*GS3*	*Aromatic*	103	1.914**	1.897**	0.240	0.984
	*Indica*	103	2.800**	3.799**	0.319	3.582***
	*Japonica*	84	1.712**	1.987**	0.225	1.735
	*Landrace*	87	2.076**	2.569**	0.304	2.282*
	*Wild derivatives*	105	1.695**	1.976**	0.312	1.782
*qSW5*	*Aromatic*	56	1.844**	1.826*	0.253	0.940
	*Indica*	23	1.835**	0.830	0.095	−0.979
	*Japonica*	16	1.522	1.635	0.092	1.154
	*Landrace*	37	1.886	1.557	0.187	0.265
	*Wild derivatives*	76	1.422	1.316	0.046	0.406
*sd1*	*Aromatic*	33	1.751	1.406	0.175	0.033
	*Indica*	34	2.101	0.955	0.094	−0.956
	*Japonica*	10	1.416	0.914	0.047	−0.842
	*Landrace*	21	1.700	0.869	0.097	−1.104
	*Wild derivatives*	1	0.716	0.508	0.005	−0.341

***p < 0.001; **p < 0.02; *p < 0.05.

The *Ghd7* gene did not show any significant linkage disequilibrium (LD) blocks in the sequenced region. Eight marker-trait associations (MTA) were observed for the trait ‘number of panicles’ with a PVE (Phenotypic Variance Explained) range of 10.1–21.8% in ARS, Nellore. In case of RARS, Maruteru, six MTAs for EY, FG and SP traits were observed with 12–17.5% phenotypic variation (Supplementary Table S4).

**Figure 3:**
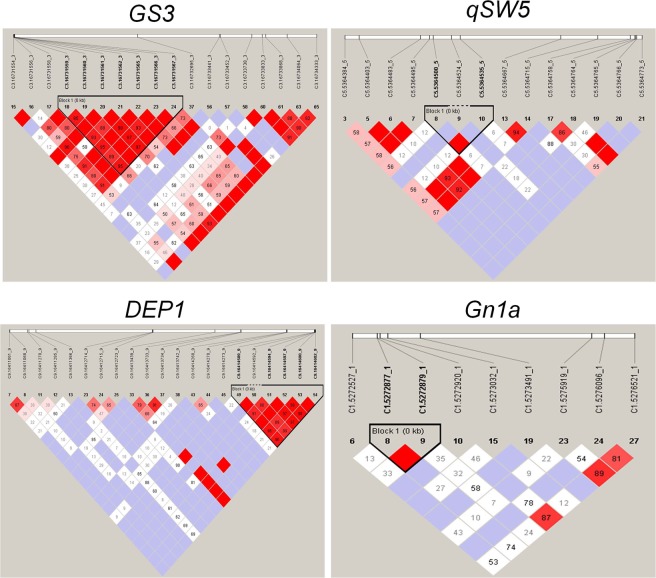
Linkage disequilibrium (LD) patterns of the targeted yield genes in the rice genotypes. LD is shown by the R^2^ value, with white R^2^ = 0, light red for 0 < R^2^ < 1 and Red for R^2^ = 1. The LD blocks are highlighted with black triangle in the diagram. LD blocks were identified as described in methods part.

In all, 21 haplotypes were constructed using 47 SNPs from *Ghd7* gene. Of them, *Ghd7-1* was shared, surprisingly, by 80% of the total genotypes which includes all five sub populations. Interestingly, 100% *japonica* varieties are falling under the haplotype, *Ghd7-1*. Remaining haplotypes were represented by very few varieties. Phylogenetic analysis revealed total five clades, in which, notably, *Ghd7-14* haplotype consisting of two *indica* genotypes, *i*.*e*., Nilagiri and RNR19186, which formed a separate outgroup compared to other clusters (Supplementary Fig. S1; Supplementary Table S5). Earlier, Lu *et al*.^13^ reported 16 haplotypes and two clades with 76 SNPs and six indels while studying with 104 accessions in *Ghd7* gene in rice. Moreover, there was a significant difference found among the *Ghd7* derived haplotypes for number of panicles trait (Supplementary Fig. S3) indicating their potential applicability in rice breeding for broadening the genetic base.

#### DEP1(Dense and Erect Panicle1)

The *DEP1* gene found to significantly increase the grain yield by regulating erect panicle and number of grains per panicle besides nitrogen uptake and stress tolerance. *DEP1* locus is a gain-of-function mutation causing truncation of a phosphatidylethanolamine-binding protein-like domain protein^6^. The whole genome DNA resequencing of *DEP1* gene (4.7 kb) revealed, altogether, 106 SNPs and 22 indels (Fig. 1B). This number is far more than the variations (SNP-45 and indel-26) identified by Zhao *et al*.^14^ using 72 rice germplasm. The majority of the variations are located in the intronic regions (57) followed by the promoter (15), exonic-CDS (14) regions of the gene. Astonishingly, present study revealed more number of total alleles than RiceVarMap and novel alleles at this locus (Fig. 2A). Interestingly, 10 missense mutations were found in this gene, and among them, four were novel alleles without any polarity change in the amino acid side chains (Table 2). Among the rest of the six missense mutations, two each with non-polar to polar and polar to non-polar and one each with polar to polar and non-polar to basic polar changes of amino acid side chains was found. Of 10 missense mutations, six were due to transversions and four were due to transitions. Among group-specific alleles, *indica* has a major share (24) of alleles followed by *landraces* (6). Apart from indels found in *aromatic* group, all other groups were identified with novel indels only in this gene (Supplementary Table S3; Fig. 2).

**Table 2:** The details of non-synonymous alleles identified in the present study.

Gene	SNP position	Position in the gene	Nucleotide change	Amino acid change	Side chain class/polarity	Associated trait (in ARS, Nellore)
*qSW5*	5365232	*Exonic-CDS*	*TCG* > *TCGTCGTAC*	*Serine (p*.*S37)*	*Polar*	
	5365233	*Exonic-CDS*	*AGG* > *ACGTACGGG*	*Arginine (p*.*R38)*	*Basic*	
	5365234	*Exonic-CDS*	*AGG* > *ACG*^††^	*Arginine to Threonine (p*.*R38T)*	*Basic polar to Polar*	Grain weight, Harvest index
	5365236	*Exonic-CDS*	*GAC* > *TAC*^††^	*Aspartic acid to Tyrosine (p*.*D39Y)*	*Acidic polar to Polar*	Grain weight
*DEP1*	16412253	*Exonic-CDS*	*TGC* > *GGC*^††^	*Cystein to Glycine (p*.*C18G)*	*Non polar/Non polar*	
	16414268	*Exonic-CDS*	*CTT* > *ATT*^††^	*Leucine to Isoleucine (p*.*L88I)*	*Non polar/Non polar*	
	16414271	*Exonic-CDS*	*TTT* > *CTT*^†^	*Phenylalanine to Leucine (p*.*F89L)*	*Non polar/Non polar*	
	16414273	*Exonic-CDS*	*TTT* > *TTA*^††^	*Phenylalanine to Leucine (p*.*F89L)*	*Non polar/Non polar*	
	16414735	*Exonic-CDS*	TAC > TGC^**†**^	Tyrosine to Cystein (p.Y105C)	Polar/Non polar	
	16415032	*Exonic-CDS*	AGC > AAC^**†**^	Serine to Aspergine (p.S204N)	Polar/Polar	Chaffy grains, Spikelets per panicle
	16415104	*Exonic-CDS*	CTT > CAT^**††**^	Leucine to Histidine (p.L228H)	Non polar/Basic polar	
	16415203	*Exonic-CDS*	TGC > TAC^**†**^	Cystein to Tyrosine (p.C261Y)	Non polar/Polar	
	16415254	*Exonic-CDS*	TCG > TGG^**††**^	Serine to Tryptophan (p.S278W)	Polar/Non polar	Filled grains, Spikelets per panicle
	16415391	*Exonic-CDS*	TGC > AGC^**††**^	Cystein to Serine (p.C324S)	Non polar/Polar	Grain length

Note: Novel alleles are highlighted in italics; Transitions^†^ and Transversions^††^.

For *DEP1*, highest nucleotide diversity was observed in the *aromatic* group while low in *japonica* group. Neutrality analysis of Tajima’s D indicated non-significant positive values in all five groups while rest two methods showed significant positive values except in *landraces* in Fu and Li’s F* test wherein it showed non-significance indicating the evidence of balancing selection at this locus (Table 1). These results are not in good agreement with the previous report in which they found significant negative values for Tajima’s D indicating that the *DEP1* gene has undergone positive selection^14^.

The pattern of LD in the *DEP1* gene showed one tightly correlated block of LD between C9.16414589 to C9.16414602 region (Fig. 3). Of them, interestingly, two SNPs, *i*.*e*., C9.16414600 and C9.16414602 showed significant association with the seed width trait in ARS, Nellore. In all, 24 SNP trait associations for nine yield component traits with a PVE range of 6.3–17.14% were found in ARS, Nellore region, whereas only six associations for two traits were found in RARS, Maruteru in this locus. Notably, the SNP C9.16411020 identified in RARS, Maruteru explained maximum of 22.88% PVE. In addition, three non-synonymous SNPs (nsSNPs) located in 16415032, 16415254 and 16415391 positions of the exonic-CDS were found to be associated with CG, FG and GL while C9.16415254 and C9.16415032 showed association with SP (Supplementary Table S4).

#### Gn1a (Grain number per panicle)

*Gn1a (OsCKX2)*, which encodes *cytokinin oxidase/dehydrogenase*, is the first gene to be isolated that govern one of the vital grain yield-related traits, *i*.*e*., grain number in rice. Reduced expression of *Gn1a* leads to cytokinin accumulation in the inflorescence meristems thereby increases the grain number^5^. The resequencing of *Gn1a* gene revealed 50 SNPs and 28 indels in the 4.67 kb alignment. Of them, the majority (59) were located in the intronic region while remaining 11 were located in the downstream region and eight were in the exonic 5’UTR region of the gene (Fig. 1B). Among the 78 total alleles, 34 were found to be novel alleles while 44 were common alleles with RiceVarMap database (Fig. 2A). As far as population-specific alleles are concerned, *indica* group consisted majority (21) of them comprising both SNPs and indels; of them, 11 were novel SNPs, and seven were novel indels (Supplementary Table S3; Fig. 2). Wang *et al*.^11^ identified 17 SNPs and five indels by DNA sequencing of the *Gn1a* gene using 175 cultivars and 21wild rice accessions.

Highest nucleotide diversity was observed in the group that consists of *landraces* than in the other group. All five groups showed non-significant positive values with *japonica* group being the high value in Tajima’s D test except in the *wild derivatives*, which displayed negative values due to the positive selection (Table 1). Contrast to the present findings, Wang *et al*.^11^ identified non-significant negative values except *japonica* like accessions implying artificial selection of *Gn1a* gene during the domestication. The *landraces* and *japonica* groups showed significant highest positive values in Fu and Li’s D* and F* neutrality tests, respectively.

One LD block was found in *Gn1a* gene comprising two SNPs, *viz*., C1.5272877 and C1.5272879 (Fig. 3). Marker-trait association analysis revealed four SNPs as significantly associated with CG and HI traits with phenotypic variation ranging from 7.8 to 9.6% in ARS, Nellore while eleven MTA were recorded for NP, EY, GWT and PH traits with a phenotypic variance range of 9.0–17.3% at RARS, Maruteru (Supplementary Table S4).

Using 12 SNPs from *Gn1a* gene, 25 haplotypes were constructed. Of them, *Gn1a-2* was shared by 46.7% of the total genotypes followed by *Gn1a-1* with 16.48%, which includes both *indica* and *japonica* genotypes. Remaining haplotypes were represented by very few varieties. The predominant haplotypes, *Gn1a-2*, *Gn1a-1*, *Gn1a-5* and *Gn1a-7*, formed a separate cluster. Interestingly, some of the rare haplotypes also formed a separate cluster. For instance, *Gn1a-17* and *Gn1-19* haplotypes represented *landraces* of Hasan sona and Halya mamo, respectively (Supplementary Fig. S1). Interestingly, the *Gn1a-9* haplotype comprising of exclusively biotic and abiotic stress tolerant genotypes such as BM71- Brown plant hopper resistant, Tetep - Blast and bacterial leaf blight resistant while Disang, Kapilee and Mrunalini are drought tolerant genotypes. With 17 SNPs and 5 indels of *Gn1a* Wang *et al*.^11^ discovered 22 haplotypes of A1 to A22 (Supplementary Table S6).

#### GS3 (Grain Size and weight)

The major QTL, *Grain size 3 (GS3)* contributes to grain length and weight in rice, and it encodes a transmembrane protein with four putative domains^8^. The resequencing of 6 kb length of the *GS3* gene revealed 88 SNPs and all were located in the downstream of the gene (Fig. 1B). Of these 88 SNPs, 33 were found to be novel while 55 were common to RiceVarMap database (Fig. 2A). The *indica* group comprised maximum group-specific alleles (15) while remaining groups have one each except *japonica* group that has nil alleles (Supplementary Table S3; Fig. 2). Surprisingly, the resequenced region of the *GS3* gene consisted neither indels nor any novel alleles. Earlier, a total of 78 SNPs and 26 indels were identified by sequencing of the *GS3* gene in 10 strains of rice^12^.

The Pi value of nucleotide diversity was higher in the *indicia* group than the other groups. Notably, the *indica* group exhibited significant positive and highest values in all three neutrality tests suggesting apparent balancing selection acting on this gene (Table 1). Contrary to the present results, Takano *et al*.^12^ observed positive selection of the *GS3* locus.

In the *GS3* gene, one large LD block comprising seven SNPs was found between C3.16731559 and C3.16731567 (Fig. 3). Of them, two SNPs *viz*., C3.16731566 and C3.16731567 were significantly associated with the number of panicles in ARS, Nellore. In all, as many as 20 MTAs were found to be associated with seven traits with PVE range of 5.0–13.7% in ARS, Nellore; whereas in RARS, Maruteru, only seven SNPs for five traits were found to display the significant associations with phenotypic variation of 9.9–17.5% (Supplementary Table S4).

In the *GS3* gene, as many as 111 haplotypes were constructed with 89 alleles. Of them, *GS3-44* haplotype comprising maximum number (23) of genotypes followed by *GS3-45* (18) and *GS3-48* (14). Apparently, remaining are all genotype-specific rare haplotypes (Supplementary Fig. S1; Supplementary Table S6). Takano *et al*.^12^ identified three haplotypes and concluded that 320-bp and 13-bp deletions occurred in a *japonica-like* ancestor and that 4-bp and 1 + 3-bp deletions occurred in an *indica-like* ancestor. However, these deletions were not observed in the present study due to the fact that all of the nucleotide variations were located in the downstream region of the *GS3* gene.

#### qSW5 (Seed width)

Another major grain size related QTL, *qSW5* is located on chromosome 5 that specifically determines grain width in rice^10^. The 2.26 kb length of the *qSW5* gene resequencing uncovers a total of 57 nucleotide variations consisting of 43 SNPs and 14 indels, which is lower than the earlier study where they uncovered 67 SNPs and two indels using 127 rice varieties^16^. Among them, 93% of the alleles were located in the downstream region of the gene while rest were located in the exonic-CDS region; interestingly, all are having the missense mutations (Fig. 1B). Notably, more number of total alleles and novel alleles were found at this locus compared to RiceVarMap database. Twenty-nine of 57 were found to be novel alleles while the remaining 28 were commonly found in RiceVarMap also (Fig. 2A). The two missense novel mutations were observed at 5365234 bp and 5365236 bp of the gene causing amino acid changes of Arginine to Threonine (p.R38T) and Aspartic acid to Tyrosine (p.D39Y) due to the transversions with a polar change of the basic polar to polar and acidic polar to polar, respectively (Table 2). Maximum number of group-specific alleles was identified in the *indica* group (9). Surprisingly, all the population-specific alleles in the *qSW5* gene are novel except one indel that is specific to the *aromatic* group (Supplementary Table S3; Fig. 2).

The genotypes that fall in *aromatic* group showed the highest values in nucleotide diversity analysis than other group genotypes. In Tajima’s D neutrality test, although it has showed non-significance, *japonica* group showed positive values while *indica* group showed negative values (Table 1). This can be explained by the fact that the selection for medium slender grain types has been acting in most of the *indica* genotypes included in the study. Lu *et al*.^16^ reported negative values of Fu and Li’s D and found to be significantly deviated from the neutrality in *qSW5* gene.

One distinct LD block comprising three SNPs *viz*., C5.5364500, C5.5364524 and C5.5364535 was identified in the *qSW5* locus (Fig. 3). Candidate gene association analysis revealed significant association between four SNPs and grain weight and harvest index with PVE of 6.0–7.6% at ARS, Nellore, while only one association was found in RARS, Maruteru for panicle length, that explained maximum of 20.8% phenotypic variation. Interestingly, two nsSNPs from the exonic-CDS of the gene, C5.5365234 and C5.5365236showed significant association with grain weight and harvest index whereas, C5.5365234 with harvest index (Supplementary Table S4).

**Figure 4:**
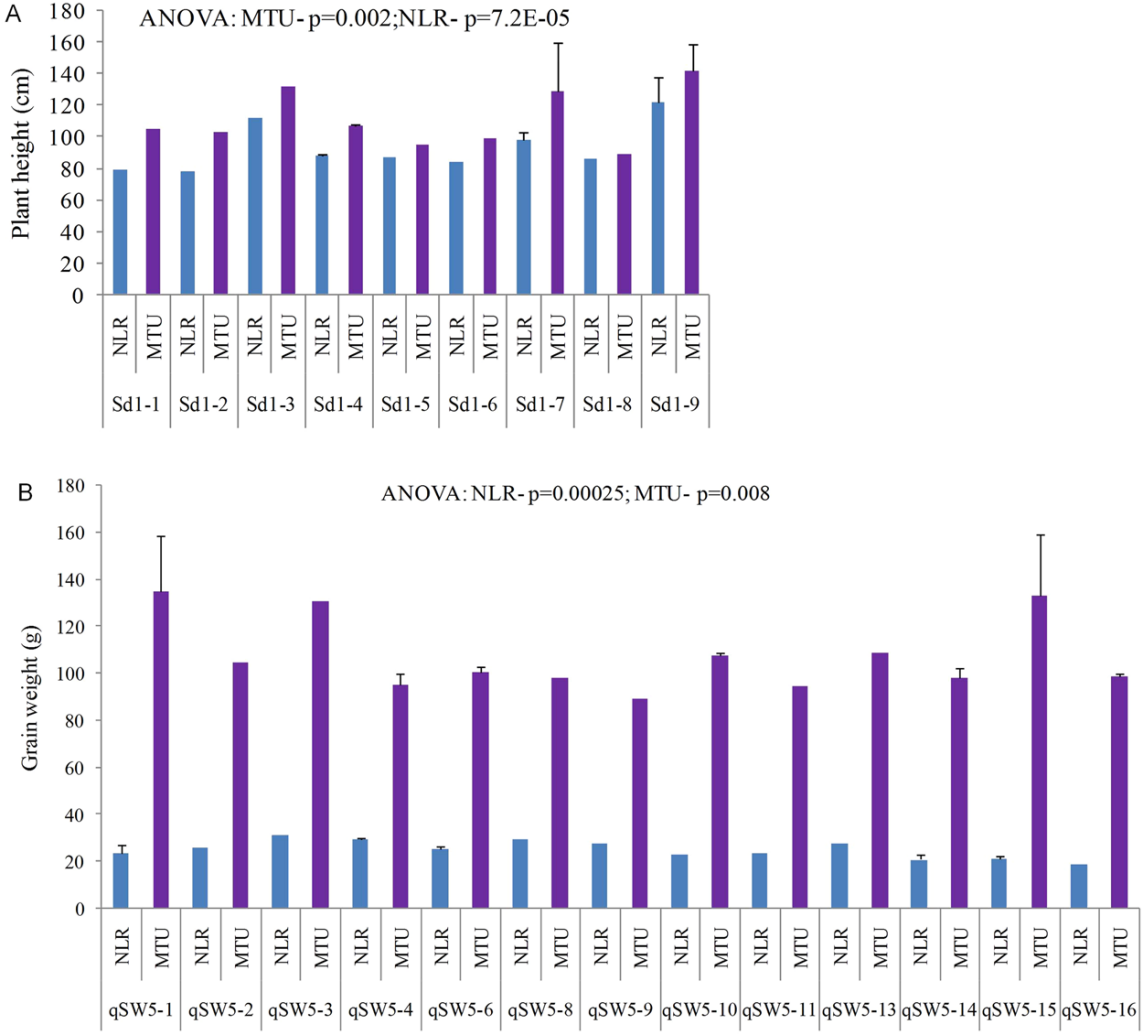
Comparison of mean values of the haplotypes of *sd1* gene for plant height (**A**), and *qSW5* (**B**) gene (**A**), for grain weight (recorded in both RARS, Maruteru [MTU] and ARS, Nellore [NLR]. The significant differences among the haplotypes were estimated using analysis of variance (ANOVA). p = Probability.

In *qSW5* gene, 19 haplotypes were constructed using 14 SNPs. Of them, *qSW5*-10 haplotype was shared by 70.9% of the total genotypes, which includes both *indica* and *japonica* genotypes. Like earlier genes, some of the rare haplotypes were also observed. For instance, *qSW5*-3 and *qSW5*-5 haplotypes were represented by single genotypes of MGD101 and VLDHAN66, respectively (Supplementary Fig. S1). Further, there is a significant difference found between the haplotypes *qSW5-10* and *qSW5-4* as far as grain weight is concerned (Fig. 4).

#### sd1 (Semi dwarf 1)

The major Green Revolution gene, *sd1 (Semi dwarf 1)* encodes *Gibberellic acid* 20 *Oxidase* (*GA20OX2*) enzyme that regulates plant height in rice^17^. The resequencing of 3.1 kb *sd1* gene in all genotypes revealed 21 indels and 33 SNPs. Of them, the majority (44.4%) were found to be located in the promoter region followed by exonic 5′UTR (35.2%), intronic (14.8%) and exonic-3′UTR (5.55%) regions of the gene (Fig. 1B). Among 54 alleles, 26 were found to be novel while 28 were common alleles (Fig. 2A). The group-specific alleles were observed in *indica* (4), *japonica* (1) and *wild derivatives* (1). All the four alleles identified in *indica* were found to be novel (Supplementary Table S3; Fig. 2). Previously, sequence analysis of the *sd1* locus of 57 semi-dwarf varieties showed to identify seven alleles, which have been used in the breeding of semi-dwarf rice varieties in China, USA and Japan^15^.

The *aromatic* group showed highest significant positive values than the other groups in the nucleotide diversity analysis. Surprisingly, all groups except *aromatic* group showed negative values with *landrace* group being the highest in Tajima’s D test. All groups except *wild derivatives* showed significant positive values with *indica* group being the highest in Fu and Li’s D* test while *aromatic* group showed highest non-significant value in Fu and Li’s F* test (Table 1). It is a clear case of positive selection acting on the *sd1* gene. These findings are in line with Reagon *et al*.^18^.

There are no LD blocks and SNP-trait associations found in *sd1* gene in RARS, Maruteru. However, one significant association of the SNP, C1.38381676 with the number of panicles was identified having a phenotypic variance of 11.6% in ARS, Nellore (Supplementary Table S4).

In this gene, using 28 SNPs, 17 haplotypes were constructed. The predominant haplotype, *sd1*-5 was shared by 80.7% of the total genotypes, which includes all sub-populations that were included in the study followed by *sd1-16*, which comprising of *aromatic*, *indica* and *landraces* only. Interestingly, the source for the Green Revolution allele (383 bp deletion) of the *sd1* gene *i*.*e*., Dee-Gee-Woo-Gen (DGWG) also belonging to the large haplotype *sd1-5* suggesting that strong selection was evident for this allele in the rice breeding since 1960s (Supplementary Fig. S1). Interestingly, there is a significant difference in the varieties carrying the haplotypes *sd1-5* and *sd1-16* for both plant height and culm height traits (Fig. 4).

### Allele mining analysis in the targeted yield contributing genes

In the present study, using as many as 200 rice genotypes, in all, 543 nucleotide variations including SNPs and indels were recorded across all six genes, which is much higher than the previous studies^11–15^. The higher frequency of SNPs and indels observed in the present study could be attributed to the inclusion of diverse accessions of rice i.e., *aromatic*, *indica*, *japonica*, *landraces* and *wild derivatives*. In addition, we identified 225 novel alleles in the present study only when compared to the total number of respective genic alleles that are present in the RiceVarMap database, which has the repository of resequencing data of more than 4000 rice accessions. Besides, we uncovered 128 alleles exclusively in certain sub-populations, especially high number in the *indica* group. The *indica* group largely comprising of modern cultivars released for cultivation post-Green Revolution era of the 1960s. The untapped group-specific alleles are of great value to broaden the genetic base of the cultivars. However, in order to accurately determine the effects of these novel and population-specific alleles, near-isogenic lines containing each of these alleles has to be constructed before being exploited in the rice breeding.

In addition, the non-synonymous alleles were found in exonic coding regions of two genes, *qSW5* and *DEP1*. Four alleles found in *qSW5* were novel, and they showed alteration of amino acids from one group to the other. In case of *DEP1* gene also, five of ten non-synonymous alleles have produced different amino acid groups largely from polar to non-polar group. Since two amino acids belong to the different groups, the changes in the structure/function of the protein could be profound, which may be reflected in the phenotype as well. Although most of the indels and SNPs identified in the present study in targeted yield genes may not correspond to amino acid alteration, they might have profound implications in marker-assisted breeding and genome-assisted breeding, respectively. However, further studies warranted to establish the role of these nsSNPs in determining phenotype of the yield traits. The non-synonymous SNPs identified in the present study in the protein-coding exonic regions of *DEP1* and *qSW5* might provide a beneficial source of functional markers, which can be used in maker-assisted breeding programs to transfer the desired QTLs as no recombination can separate this marker from the associated phenotype.

Generally, the nucleotide variations in the protein-coding regions of a gene or exons are known to influence the protein structure or function thereby the phenotype ultimately. However, growing evidence demonstrates that the non-coding regions of a gene including untranslated regions (UTRs), introns and promoters also regulate the gene expression. Samadder *et al*.^19^ demonstrated the transcriptional and post-transcriptional enhancement of gene expression by the 5′UTR intron of rice *rubi*3 gene. In the present study also, from all six genes, 10% of total alleles were located in the UTR region, and 8% were in the promoter region. Further characterization of the expression of these alleles would throw more light on the respective yield gene applicability in the rice breeding.

### Candidate gene association analysis

The candidate gene association has been extensively applied to discover sequence variants associated with many traits in rice, such as yield-related, disease resistance, quality characters etc.^20,21^. In this study, the association between yield-related traits and allelic variants underlying six targeted yield genes were assessed to identify favourable alleles having the potential to improve grain yield. In total, we identified 61 MTAs in ARS, Nellore while 31 in RARS, Maruteru for six and five genes, respectively. Interestingly, SNPs from the exonic-CDS of *DEP1* for CG, FG, GL, SP and *qSW5* for GW and HI traits also showed the association. The alleles that are located in exonic-CDS and associated with the yield component traits are of great value as they significantly impact the phenotype when transferred to the recipient background. The favourable alleles identified in the present study can be used as a resource for development of functional markers to use in molecular breeding. Furthermore, combining favourable alleles of multiple genes has the potential to produce high yields than using single favourable alleles alone.. The current study revealed that previously identified nucleotide variation in the six genes does not exclusively contribute to the corresponding traits. For instance, the 383 bp deletion that supposed to cause dwarfness^17^ in the *sd1* was not found in the present study. These results are in consistent with the Lu *et al*.^16^.

### Artificial selection of yield genes

During the course of domestication, important yield attributing traits have been subjected to artificial selection, which subsequently reduced the genetic diversity near the selected gene-regarded as selective sweep or conserved haplotype or signature of selection. In this study, both positive (*Ghd7*) and balancing (*GS3*) selection has been observed in the evolution of these targeted yield genes, especially *indica* genotypes. Main motto after the success of Green Revolution is to enhance the yield; hence, this gene might have been positively selected by the breeders in their breeding programmes over the decades. However, the *GS3* gene has exhibited balanced selection due to the diverse interests of the consumers in the world for various grain size traits within *indica* genotypes. For instance, long and slender grain of *indica* rice is preferred by the consumers of India, China, Thailand, Pakistan and USA, while short grain *japonica* rice is preferred in Japan and Koreas. Even, within India, North Indians prefer Basmati-like long slender grain while South Indians favor Samba mahsuri-like medium slender grain varieties. These varied preferences for the single grain size trait prompedt breeders to develop varieties as per the consumer preferences, which ultimately results in balanced selection.

### Haplotype analysis

An efficient approach to overcome the biallelic limitation of SNPs is to employ haplotypes- the specific combination of jointly inherited nucleotides or markers from polymorphic sites in the same chromosome segment^22^. In this study, we identified many haplotypes for each targeted gene, which consisting of many rare haplotypes with few genotypes as well. Among them, the high yielding variety, BPT1768 (Bapatla sannalu), notably, found to have maximum number of rare haplotypes for three genes *viz*., *Gn1a-8*, *qSW5-12* and *GS3-29* (Supplementary Table S6 and S9). It is also evident that the so called “Mega varieties of rice” such as BPT5204, MTU1010, MTU1001, Swarna and Swarna Sub1, all comes under the same haplotype in the targeted genes warranting to broaden the genetic base of these varieties using the genotypes with rare haplotypes. Hence, the genotypes that come under the rare haplotypes can be of potential value for downstream rice breeding as donors for replenishing the most shared alleles. We observed significant haplotype differences in two genes, i.e., *sd1* for plant height and *qSW5* for grain weight in two environments (RARS, Maruteru and ARS, Nellore) and in one environment (ARS, Nellore) for two genes i.e., *Ghd7-1* and *DEP1* for number of panicles (Fig. 4 and Supplementary Fig. S3) implying the fact that they have prospects in the current rice breeding programmes for yield enhancement.

### Population structure and phylogenetic analysis

**Figure 5:**
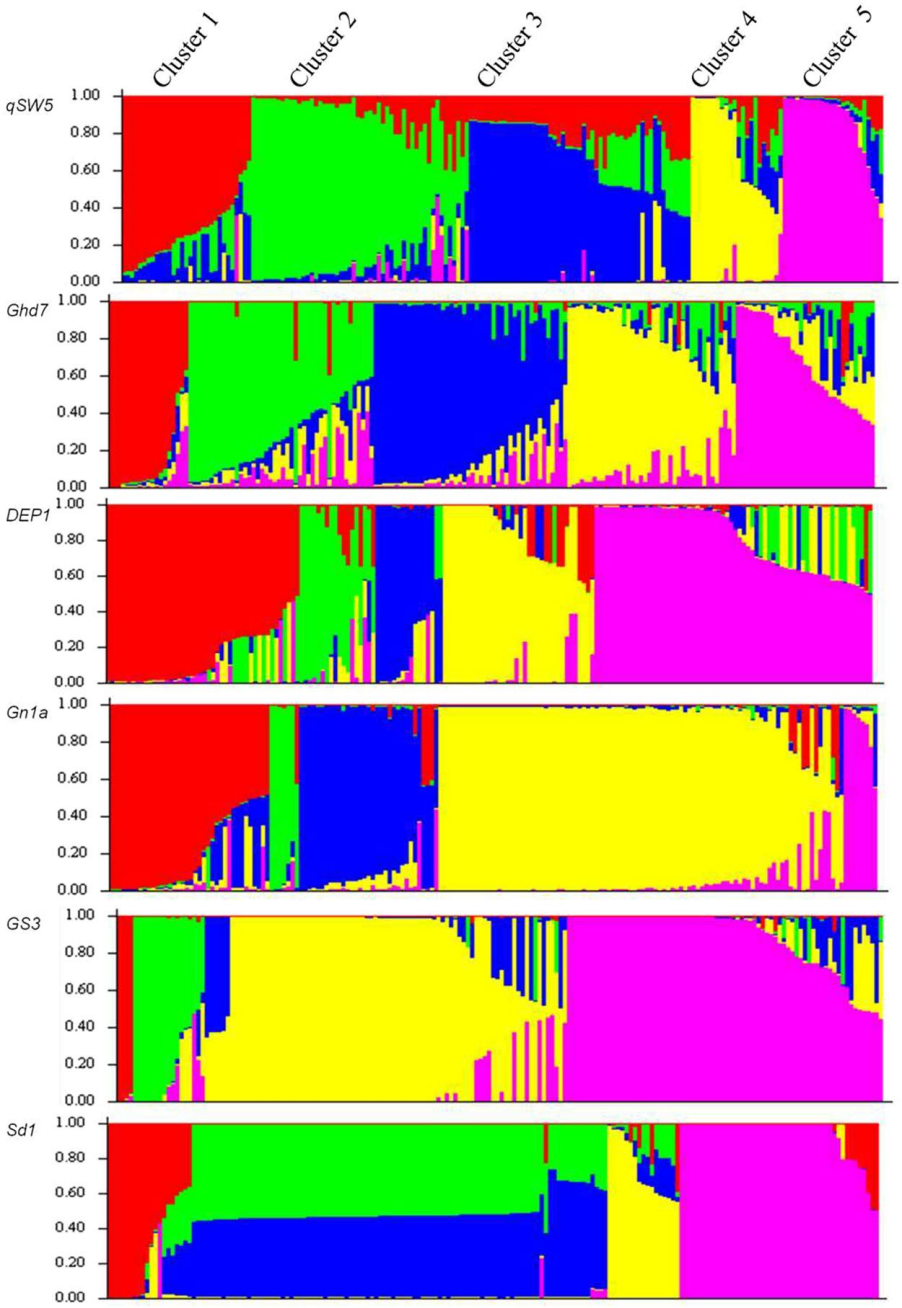
Population structure of the rice genotypes. The population structure analysis was done using STRUCTURE software. The five colours indicate the Clusters 1–5; Cluster 1(Red), Cluster 2 (Green), Cluster 3 (Blue), Cluster 4 (Yellow) and Cluster 5 (Purple). Every genotype is represented by a single vertical line with the lengths proportional to each of the clusters.

Population structure analysis and phylogenetic tree constructed based on the gene-specific SNPs obtained from the rice genotypes failed to constitute population-specific clustering (Fig. 5; Supplementary Fig. S2) and largely mixed type of grouping was obtained. Widespread sharing of alleles across all populations suggests that these alleles are predominant in the varieties developed in recent times. Moreover, the fact that the selection of same gene in different populations implies that these genic variations are quite likely to be shared among populations. Recently, Thakur *et al*.^20^ have also obtained mixed type of grouping in phylogeny tree constructed using *Pi54* alleles in rice.

## Conclusion

Finding natural allelic variants for yield and its component traits is instrumental for breeding rice varieties suitable to diverse agro-ecologies and to match the different consumer preferences. In the present study, an attempt was made to find the natural nucleotide variants and to analyze the evolutionary aspects of the six important yield attributing genes *viz*., *GS3*, *Ghd7*, *Gn1a*, *qSW5*, *DEP1* and *sd1* employing NGS-based resequencing by pooled-amplicon method. The nucleotide variations obtained and their evolutionary aspects of the targeted yield genes offer great prospects for enhancing the yield in rice. Moreover, the novel, superior and population-specific alleles uncovered in the present study would pave the way for customized designing of rice to meet the future demands. Further, the alleles that associated with high phenotypic variance would be of great value for targeted trait improvement using marker assisted breeding. However, further investigation of exploring for more yield contributing genes warranted for before being exploited in the downstream rice improvement research and comprehensive elucidation of the yield traits.

## Materials and Methods

### Plant material and phenotyping

A total of 200 rice genotypes encompassing *landraces*, elite *indica* cultivars, *japonica* accessions and introgression lines derived from *indica*/wild crosses-*wild derivatives* were included in the present study The rice accessions were chosen based on their diverse nature for their important yield and its component traits and also to ensure to maintain the population structure for association mapping study. The details of accessions are provided in Supplementary Table S7. The rice genotypes were planted at two locations, *i*.*e*., Agricultural Research Station (ARS), Nellore and Regional Agricultural Research Station (RARS), Maruteru, of Acharya NG Ranga Agricultural University, Andhra Pradesh, India, in June, 2016, following augmented block design. Each genotype was planted in four rows with 11 plants each with spacing of 15 cm between plants and 20 cm between rows. Standard agronomic practices were followed as per the recommendations. The phenotypic data of yield and its component traits such as plant height (PH), culm height (CH), number of panicles (NP), panicle length (PL), spikelets per panicle (SP), filled grains (FG), chaffy grains (CG), spikelet fertility (SF), 1000 grain weight (GW) economic yield (EY), biological yield (BY), harvest index (HI), grain length (GL), seed width (SW), days to 50% flowering (DFF) and spikelet sterility (SS) was recorded from six randomly chosen plants from each genotype. The standard procedure followed for recording the observations of agronomic traits.

### DNA extraction, PCR amplification and sequencing

Fresh leaves were collected from all field-grown rice genotypes and genomic DNA was isolated using the DNeasy Plant Mini Kit (Qiagen, Hilden, Germany). The DNA quality and quantity were checked by agarose gel electrophoresisand Qubit fluorometer (Life Technologies, Carlsbad, CA, USA). The genomic DNA was equalized to 50 ng/µl based on Qubit fluorometer estimation. The complete gene sequences of the targeted six genes related to yield and its component traits viz., *Gn1a*, *Ghd7*, *GS3*, *qSW5*, *DEP1* and *sd1* were retrieved from rice genome database of Nipponbare (http://rice.plantbiology.msu.edu/pub/data/Eukaryotic_Projects/o_sativa/annotation_dbs/pseudomolecules/version_7.0/all.dir/). The overlapping primers spanning 1 kb upstream region of the gene (promoter), 5′UTR, exons, introns, and 3′UTR were used to amplify each of the targeted gene (Supplementary Table S8). The PCR was carried out in a 20 µl volume using 50 ng/ul of DNA. The 20 µl PCR reaction was prepared by taking 2 µl of genomic DNA (50 ng/µl), 2 µl of 10X Taq buffer, 0.5 µl of 1 mM dNTPs, 0.5 µl of each forward and reverse primers (both 10 pmoles), 0.1 µl (5 U/µl) of Taq DNA polymerase. The thermo profile for amplification was 94 °C for 5 min, 35 cycles of 94 °C for 30 sec, annealing at 56–60 °C for 45 sec, 72 °C for 40 sec, extension at 72 °C for 1 min and final extension at 72°Cfor 10 min and held at 4 °C. The quality of PCR products was checked by agarose gel electrophoresis. The PCR amplicons of six genes were pooled and re-sequenced using Illumina HiSeq2500 platform.

### Data analysis

The raw reads were pre-processed before considering for the downstream analysis. The adapter sequences were removed using cutadapt^23^ (version 1.8.1), followed by quality trimming [Q20] using sickle^24^ (version 1.33). The clean reads were aligned to MSU7 reference genome. The Paired-end alignment was performed using bowtie2^25^ (version 2.2.9). The variant calling was performed using samtools^26^ (version 0.1.18). The variant calling results were compiled and filtered at read depth (RD) 2, 5 and 10 using in-house custom perl scripts. The variant calling results at RD10 were considered for the downstream analysis and annotation. The variant annotation was performed based on MSU7 rice gene model using variant annotation pipeline “Varimat” (In-house pipeline developed by AgriGenome Labs). The haplotype analysis was done using Haploview^27^ software. The haplotype blocks were recognition using the algorithm proposed by Gabriel *et al*.[2002]^28^.

Population structure and kinship (*K*) of the rice genotypes was estimated employing an admixture ancestry model of STRUCTURE^29^ (version 2.3.4) software. STRUCTURE was run with five replicates for K with a run-length of 100,000 repetitions of Markov Chain Monte Carlo model following a burn-in period of 100,000 iterations. The best *K* was determined by the log likely hood of the data (LnP(*D*)) in the STRUCTURE output and an *ad hoc*statistic_*K*based on the second-order rate of change in LnP(*D*) between successive *K* values^30^. TASSEL^31^ (version 5.2.44) software was used for identifying the significant marker-trait associations (MTA) using SNP data from the resequencing of the rice varieties. Rare alleles with an allele frequency of 5% or less were removed from the dataset before the association analysis. Both Q and K matrices were used as covariates using both GLM (General Linear Model) and MLM (Multiple Linear Model) methods in the MTA analysis. The Q matrix was obtained from the STRUCTURE analysis.

Multiple sequence alignment was performed using Clustal X 2.1 (www.clustal.org) and was further edited manually. The software DNASP^32^ (version 6.11.01) (www.ub.es.dnasp) was used to analyse sequence nucleotide polymorphism and allelic diversities. Sequences were first aligned using Clustal X 2.1. The output aligned file was saved in Fasta format and was used as an input file for haplotype analysis in DnaSP (version 6.11.01). For each gene, haplotypes were constructed using DnaSP (version 6.11.01). The phylogenetic trees for each haplotype were drawn using the haplotype aligned sequences in Clustal X 2.1 and Tree View (version 1.6.6) software (https://www.treeview.co.uk). The phylogeny trees using SNPs of each gene was constructed using FigTree (version 1.4.3) software (http://tree.bio.ed.ac.uk/software/figtree/). Nucleotide diversity (Pi) and Tajima’s D^33^ and Fu and Li’s^34^ D* and F* statistical tests were used to test the evidence of neutral evolution within each group and each gene using DnaSP (version 6.11.01) software.

### Identification of novel and population-specific alleles

The nucleotide variations such as SNPs and indels from the targeted six genes were compared with the publicly available database RiceVarMap 2.0 (http://ricevarmap.ncpgr.cn/v2/)^35^. RiceVarMap 2.0 is a comprehensive database for rice genomic variation. It provides curated information of 17,397,026 genomic variations (including 14,541,446 SNPs and 2,855,580 indels) from sequencing data of 4,726 rice accessions, which includes 3243 rice accessions from 3,000 Rice Genome Project. These variations were identified based on the assembly Os-Nipponbare-Reference-IRGSP-1.0. The alleles that are identified in the present investigation and not found in the RiceVarMap 2.0 were considered as “novel”. The population-specific alleles were identified using Convert (version 1.31) software^36^.

## Supplementary information

Supplementary Information

## Data Availability

All the raw data for 200 rice genotypes has been submitted to NCBI under BioProjectPRJNA419763.
