# Supplementary material for: Uncovering of natural allelic variants of key yield contributing genes by targeted resequencing in rice (Oryza sativa L.)
Source: Sci Rep. 2019 Jun 3;9:8192. doi: 10.1038/s41598-019-44708-z (PMC6547710; doi:10.1038/s41598-019-44708-z)
Supplement: Supplementary file 1 — Supplementary Information [file 41598_2019_44708_MOESM1_ESM.pdf]

Uncovering of natural allelic variants of key yield contributing genes by targeted resequencing in rice (*Oryza sativa* L.)

Lakshminarayana R Vemireddy[1,2*†], Gopalakrishnamurty Kadambari[1†], G. Eswar Reddy[1†], Vijaya Sudhakara Rao Kola[3], Eswarayya Ramireddy[3], Venkata Ramanarao Puram[4], Jyothi Badri[5], Suresh N Eslavath[1], Swarajyalakshmi N Bollineni[1], Bukya J Naik[1], Sreelakshmi Chintala[6], Rameshbabu Pottepalem[6], Srividhya Akkareddy[2], Ranjithkumar Nagireddy[7], LachagariVB Reddy[8], Reddiah Bodanapu[8], Sivarama P Lekkala[8], Navajeet Chakravarty[8], E.A. Siddiq[7].

Supplementary Fig. S1 Haplotype analysis of the targeted yield genes. The haplotypes were constructed using DNAsp software. The phylogeny trees were constructed using Tree software.

Gn1a

Haplotype	A1	A2	A3	A4	A5	A6	A7	A8	A9	A10	A11	A12	*Aromatic*	*Indica*	*Japonica*	*Landrace*	*Wild derivatives*
Gn1a_17	G	G	G	G	G	A	G	G	C	C	A	A	0	0	0	1	0
Gn1a_19	A	A	G	G	G	G	G	G	C	C	A	A	0	0	0	1	0
Gn1a_16	G	G	G	G	A	A	G	G	C	C	A	A	0	2	0	0	0
Gn1a_15	G	G	G	A	A	A	G	G	C	C	A	A	1	9	1	0	0
Gn1a_14	G	G	A	A	A	A	G	G	C	C	A	T	0	1	0	0	0
Gn1a_13	G	G	A	A	A	A	G	G	C	C	A	A	0	1	0	0	0
Gn1a_4	G	A	G	G	G	G	A	G	T	T	A	A	0	1	0	0	0
Gn1a_5	A	A	G	G	G	G	A	A	T	T	A	A	0	10	1	2	0
Gn1a_2	G	A	G	G	G	G	A	A	T	T	A	A	6	64	3	8	4
Gn1a_1	G	G	G	G	G	G	A	A	T	T	A	A	5	14	1	8	2
Gn1a_7	A	T	G	G	G	G	A	A	T	T	A	A	0	0	0	1	0
Gn1a_6	G	A	G	A	G	A	A	G	C	T	A	A	0	1	0	0	0
Gn1a_20	G	G	G	A	G	A	A	G	C	T	A	A	0	2	1	0	1
Gn1a_12	A	A	G	G	G	G	A	G	C	T	A	A	0	2	0	0	0
Gn1a_3	G	A	G	G	G	A	A	G	C	T	A	A	0	4	0	0	0
Gn1a_9	G	A	G	G	G	G	A	G	C	T	A	A	0	5	0	1	0
Gn1a_21	G	G	G	G	G	A	A	G	C	T	A	A	0	4	0	0	0
Gn1a_18	G	G	G	A	A	A	G	G	C	T	A	A	0	0	1	0	0
Gn1a_22	G	G	A	A	A	A	G	G	C	T	A	A	0	1	0	0	0
Gn1a_10	G	A	G	G	G	G	G	G	C	C	A	A	2	2	0	2	0
Gn1a_24	G	G	G	G	G	G	A	G	C	T	A	A	0	2	0	0	0
Gn1a_11	G	G	G	G	G	G	G	G	C	C	A	A	0	1	0	0	0
Gn1a_25	G	G	G	G	G	G	A	G	C	T	C	C	1	0	0	0	0
Gn1a_8	T	T	C	C	A	A	A	A	C	C	A	A	0	1	0	0	0
Gn1a_23	G	G	G	G	C	C	A	A	T	T	A	A	0	1	0	0	0

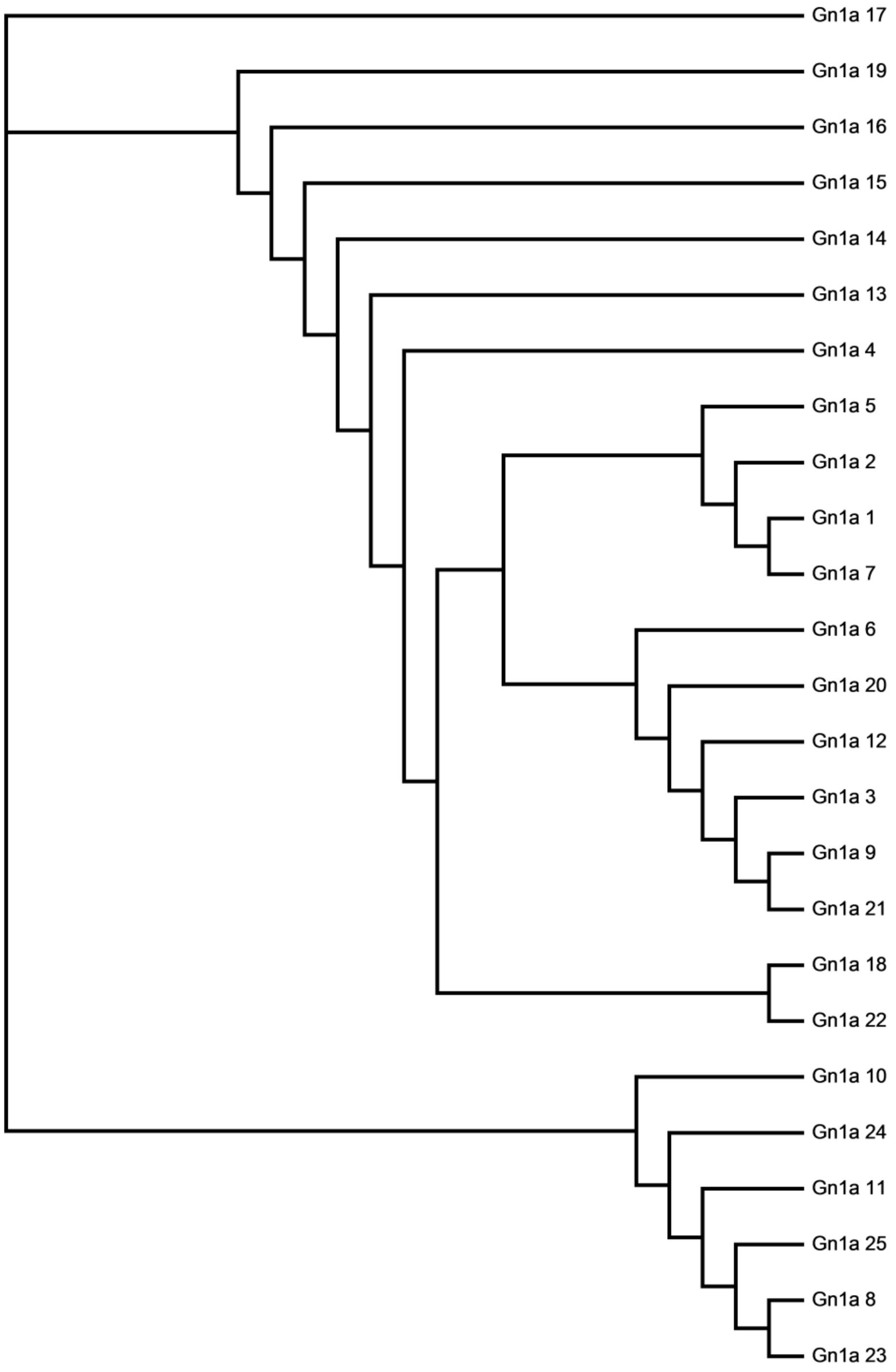

Gn1a 17
Gn1a 19
Gn1a 16
Gn1a 15
Gn1a 14
Gn1a 13
Gn1a 4
Gn1a 5
Gn1a 2
Gn1a 1
Gn1a 7
Gn1a 6
Gn1a 20
Gn1a 12
Gn1a 3
Gn1a 9
Gn1a 21
Gn1a 18
Gn1a 22
Gn1a 10
Gn1a 24
Gn1a 11
Gn1a 25
Gn1a 8
Gn1a 23

qSW5

Haplotype	A1	A2	A3	A4	A5	A6	A7	A8	A9	A10	A11	A12	A13	A14	Aromatic	Indica	Japonica	Landrace	Wild derivatives
qSW5_3	T	T	A	A	C	C	A	A	T	C	C	C	A	C	0	1	0	0	0
qSW5_5	T	T	A	A	C	C	A	A	C	C	C	C	A	A	0	1	0	0	0
qSW5_12	T	T	A	A	C	C	A	A	T	C	C	C	C	A	0	1	0	0	0
qSW5_6	T	T	A	A	C	C	A	A	T	C	C	T	A	A	0	9	0	0	0
qSW5_9	T	T	A	A	C	C	A	A	T	T	C	C	A	T	0	0	0	1	0
qSW5_7	T	T	A	A	C	C	A	A	T	T	C	C	T	C	0	1	0	0	0
qSW5_11	T	T	A	A	C	A	A	A	T	T	C	C	A	A	0	1	0	0	0
qSW5_1	T	C	A	G	C	A	A	G	T	C	C	T	A	A	2	3	0	1	0
qSW5_19	C	C	A	G	C	A	A	G	C	C	T	T	A	A	1	0	0	1	0
qSW5_17	C	C	G	G	A	A	G	G	C	C	T	T	A	A	1	1	0	0	0
qSW5_18	T	C	G	G	A	A	G	G	C	C	T	T	A	A	0	0	0	1	0
qSW5_10	T	T	A	A	C	C	A	A	T	T	C	C	A	A	8	95	6	13	7
qSW5_8	T	T	A	A	C	C	A	A	T	T	C	C	G	T	0	2	0	0	0
qSW5_13	T	T	A	A	C	C	A	A	T	T	C	T	A	A	0	1	0	0	0
qSW5_2	T	T	A	A	C	C	A	A	T	C	C	C	A	G	1	1	1	0	0
qSW5_4	T	T	A	A	C	C	A	A	T	C	C	C	A	A	0	7	1	4	0
qSW5_16	T	C	A	A	C	C	A	A	T	C	C	T	A	A	0	2	0	1	0
qSW5_14	T	C	A	A	C	C	A	A	C	C	T	T	A	A	1	2	0	1	0
qSW5_15	C	C	A	A	C	C	A	A	C	C	T	T	A	A	1	0	0	1	0

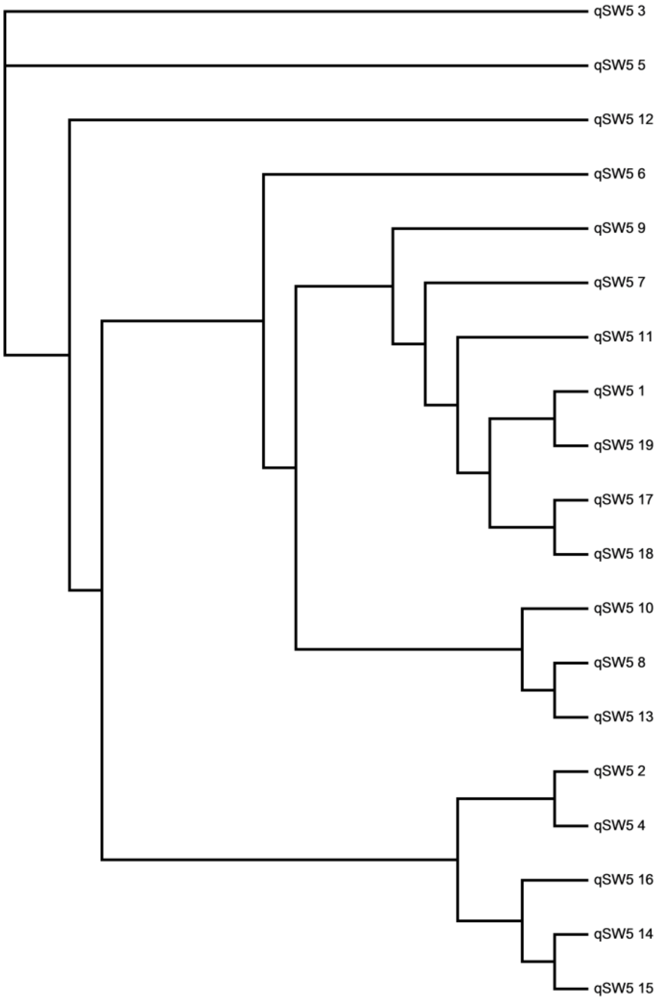

Sd1

Haplotype	A1	A2	A3	A4	A5	A6	A7	A8	A9	A10	A11	A12	A13	A14	A15	A16	A17	A18	A19	A20	A21	A22	A23	A24	A25	A26	A27	A28	Aromatic	Indica	Japonica	Landrace	Wild derivatives
sd1_6	T	T	C	C	T	T	A	A	G	G	G	G	A	G	C	C	C	C	A	A	C	C	C	C	G	G	C	A	1	0	0	1	0
sd1_1	A	T	C	G	C	T	A	A	G	A	G	G	G	G	C	T	C	C	G	A	C	C	C	C	G	G	C	A	1	3	0	1	0
sd1_17	A	T	C	C	C	T	T	A	G	G	G	G	A	G	C	C	C	C	G	A	C	C	T	C	C	G	C	A	0	1	0	0	0
sd1_13	T	T	C	C	C	T	A	A	G	G	G	A	G	G	C	C	C	T	G	A	C	C	C	C	G	G	C	A	1	0	0	2	0
sd1_14	A	T	C	C	C	T	A	A	G	G	G	A	G	G	C	C	C	T	G	A	C	C	C	C	G	G	C	A	0	1	0	0	0
sd1_2	T	T	C	C	C	C	A	A	G	G	A	A	G	G	C	C	T	T	G	G	C	C	C	C	G	G	C	A	0	2	0	0	0
sd1_10	A	T	C	G	C	T	A	A	G	G	G	G	G	G	C	T	C	C	G	A	C	C	C	C	G	G	C	A	0	1	0	0	0
sd1_12	T	T	C	C	C	C	A	A	G	G	G	G	G	G	C	C	C	C	A	A	C	C	C	C	C	C	C	A	0	1	0	1	0
sd1_3	A	A	C	C	C	C	A	A	G	G	G	G	G	G	C	C	C	C	G	G	C	A	C	C	G	G	C	A	0	0	0	1	0
sd1_16	A	A	C	C	C	C	T	T	G	G	G	G	A	A	C	C	C	C	G	G	C	C	T	T	C	C	C	A	2	4	0	1	0
sd1_4	A	A	G	G	C	C	A	A	A	A	G	G	G	G	T	T	C	C	G	G	C	C	C	C	G	G	C	A	0	5	0	0	0
sd1_11	G	G	G	G	C	C	T	T	G	G	G	G	G	G	C	C	C	C	A	A	C	C	C	C	G	G	C	A	0	0	0	1	0
sd1_8	T	T	C	C	T	T	A	A	G	G	G	G	G	G	C	C	T	T	G	G	T	T	C	C	G	G	C	A	0	0	1	0	0
sd1_15	A	T	C	C	C	T	T	A	G	G	G	G	G	G	C	C	C	C	A	A	C	C	C	C	G	G	C	A	0	1	0	0	0
sd1_9	T	T	C	C	T	T	A	A	G	G	G	G	G	G	C	C	C	C	A	A	C	C	T	T	G	G	C	A	0	1	0	0	0
sd1_5	T	T	C	C	T	T	A	A	G	G	G	G	G	G	C	C	C	C	A	A	C	C	C	C	G	G	C	A	10	107	7	16	7
sd1_7	T	T	C	C	T	T	A	A	G	G	G	G	G	G	C	C	C	C	A	A	C	C	C	C	G	G	A	T	0	1	0	0	0

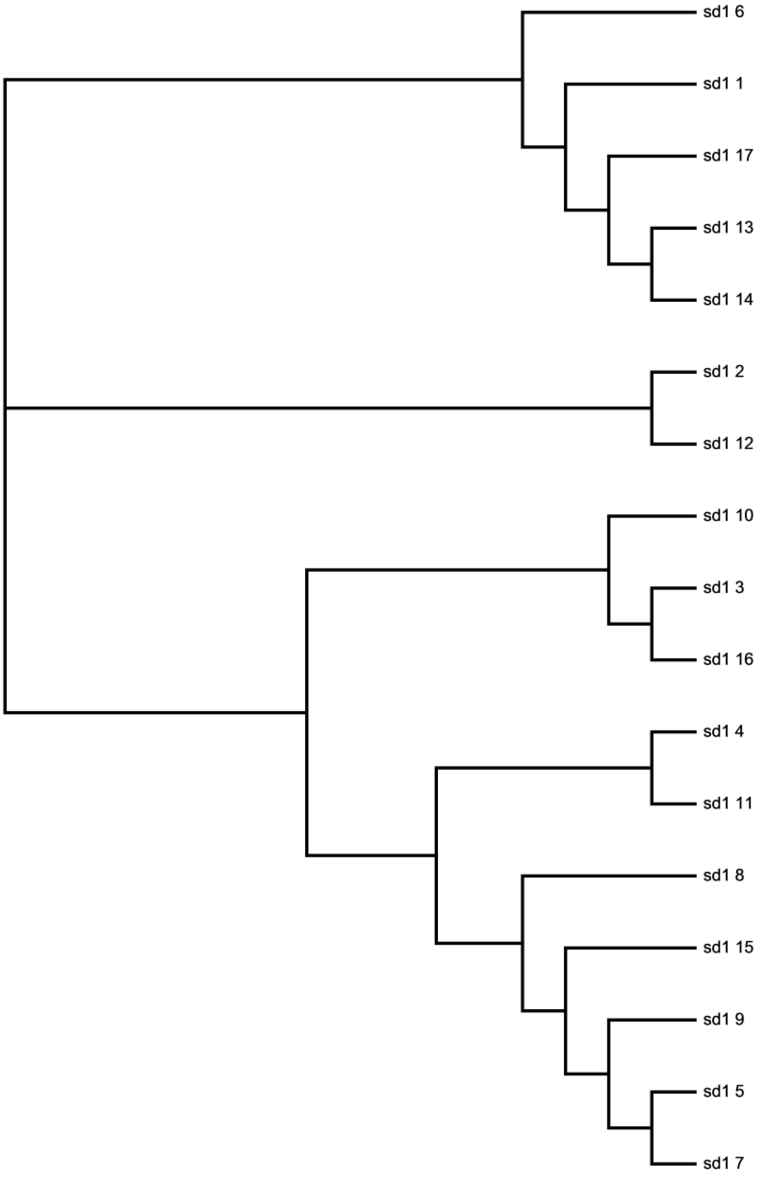

Ghd7

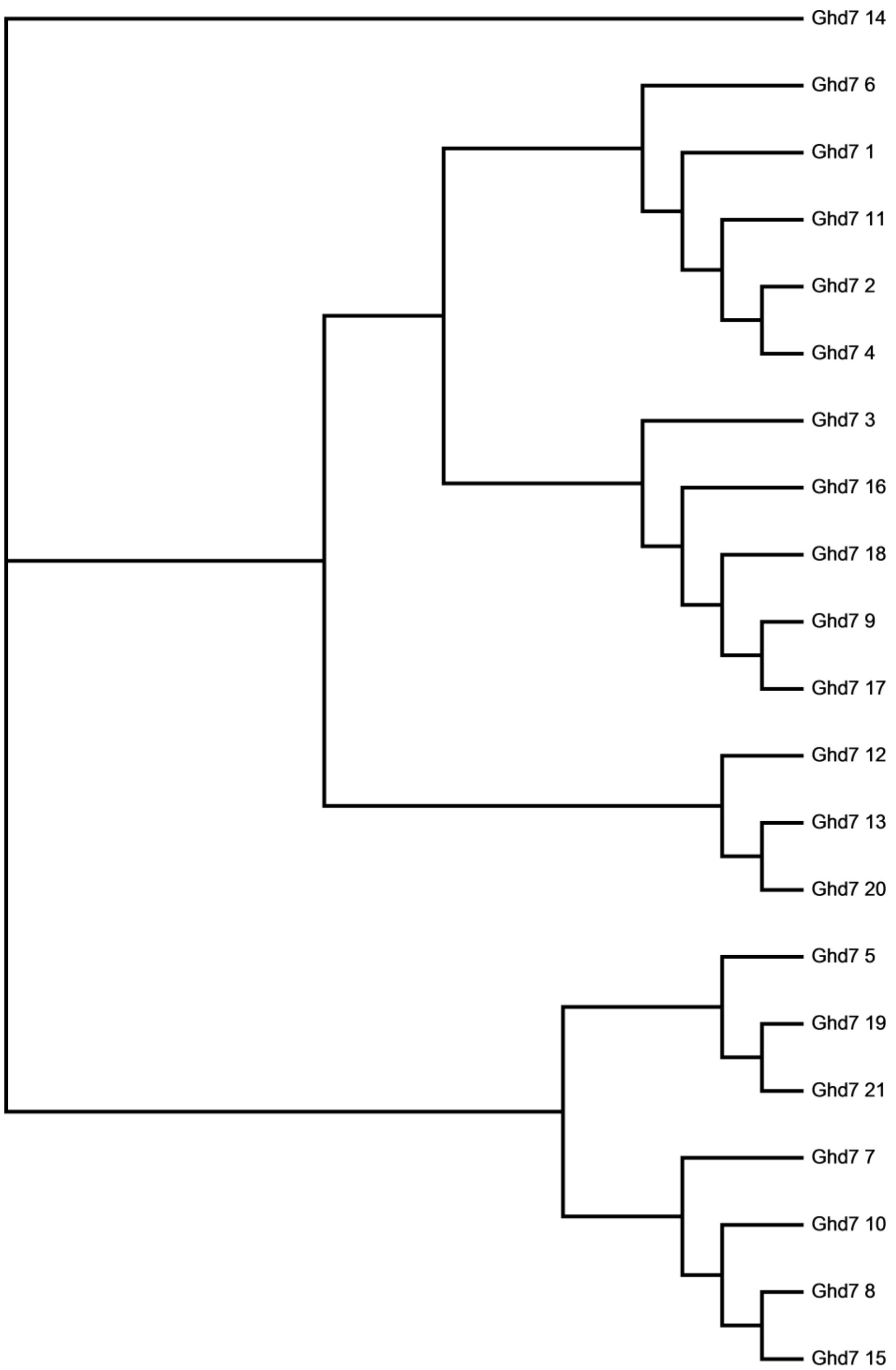

Ghd7 14
Ghd7 6
Ghd7 1
Ghd7 11
Ghd7 2
Ghd7 4
Ghd7 3
Ghd7 16
Ghd7 18
Ghd7 9
Ghd7 17
Ghd7 12
Ghd7 13
Ghd7 20
Ghd7 5
Ghd7 19
Ghd7 21
Ghd7 7
Ghd7 10
Ghd7 8
Ghd7 15

Gs3

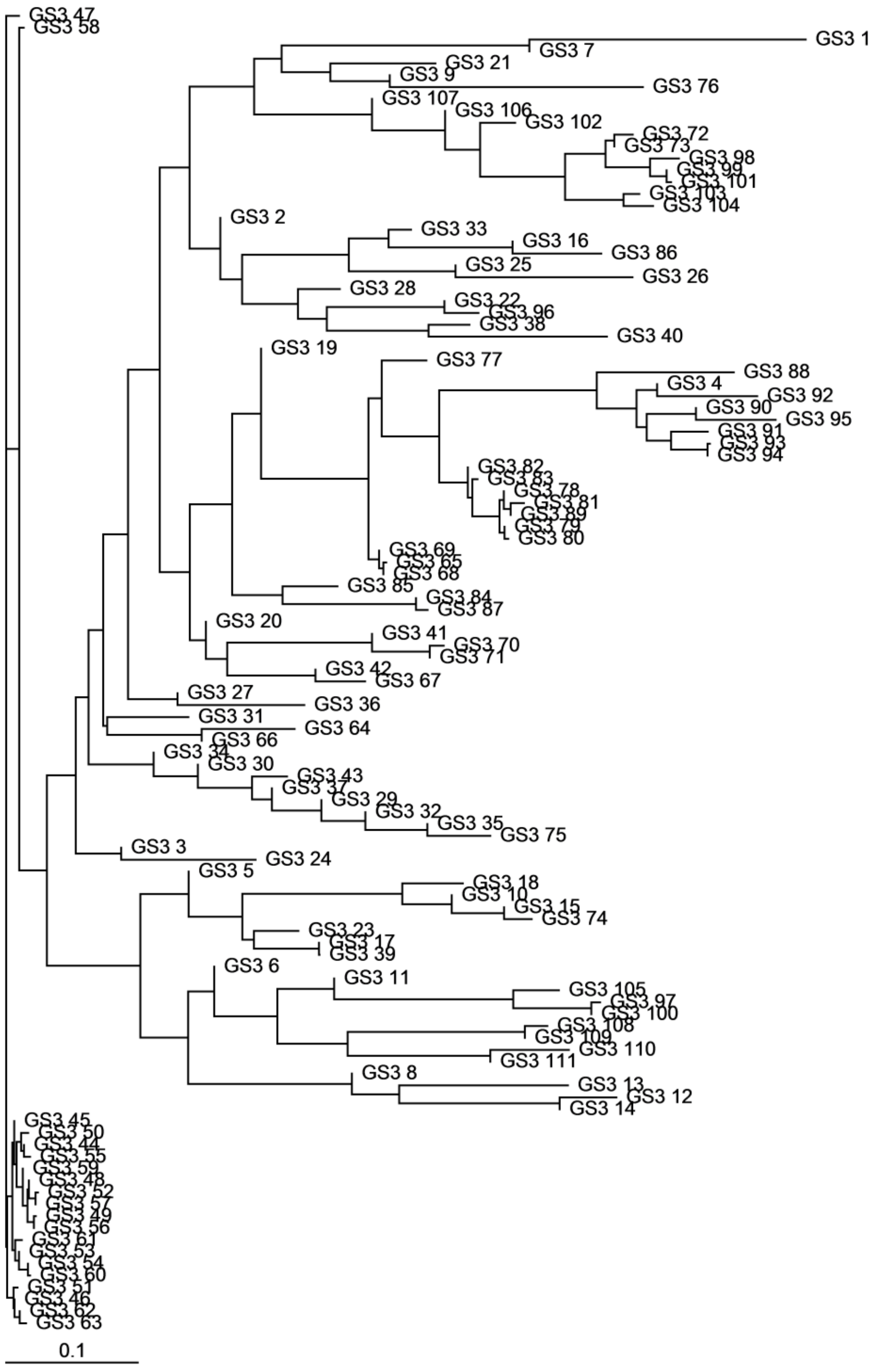

GS3 47
GS3 58
GS3 1
GS3 7
GS3 21
GS3 9
GS3 76
GS3 107
GS3 106
GS3 102
GS3 72
GS3 73
GS3 98
GS3 99
GS3 101
GS3 103
GS3 104
GS3 2
GS3 33
GS3 16
GS3 86
GS3 25
GS3 26
GS3 28
GS3 22
GS3 96
GS3 38
GS3 40
GS3 19
GS3 77
GS3 88
GS3 4
GS3 92
GS3 90
GS3 95
GS3 91
GS3 93
GS3 94
GS3 82
GS3 83
GS3 78
GS3 81
GS3 89
GS3 79
GS3 80
GS3 69
GS3 65
GS3 68
GS3 85
GS3 84
GS3 87
GS3 20
GS3 41
GS3 70
GS3 71
GS3 42
GS3 67
GS3 27
GS3 36
GS3 31
GS3 64
GS3 66
GS3 34
GS3 30
GS3 43
GS3 37
GS3 29
GS3 32
GS3 35
GS3 75
GS3 3
GS3 24
GS3 5
GS3 18
GS3 10
GS3 15
GS3 74
GS3 23
GS3 17
GS3 39
GS3 6
GS3 11
GS3 105
GS3 97
GS3 100
GS3 108
GS3 109
GS3 110
GS3 111
GS3 8
GS3 13
GS3 12
GS3 14
GS3 45
GS3 50
GS3 44
GS3 55
GS3 59
GS3 48
GS3 52
GS3 57
GS3 49
GS3 56
GS3 61
GS3 53
GS3 54
GS3 60
GS3 51
GS3 46
GS3 62
GS3 63
0.1

Supplementary Fig. S2 Phylogenetic analysis of six yield genes using SNPs from their respective genes. The phylogeny trees were constructed using FigTree software. Aromatic genotypes – Blue colour; Indica – Red ; Japonica – Purple ; Landrace – Green ; Wild derivatives – Black.

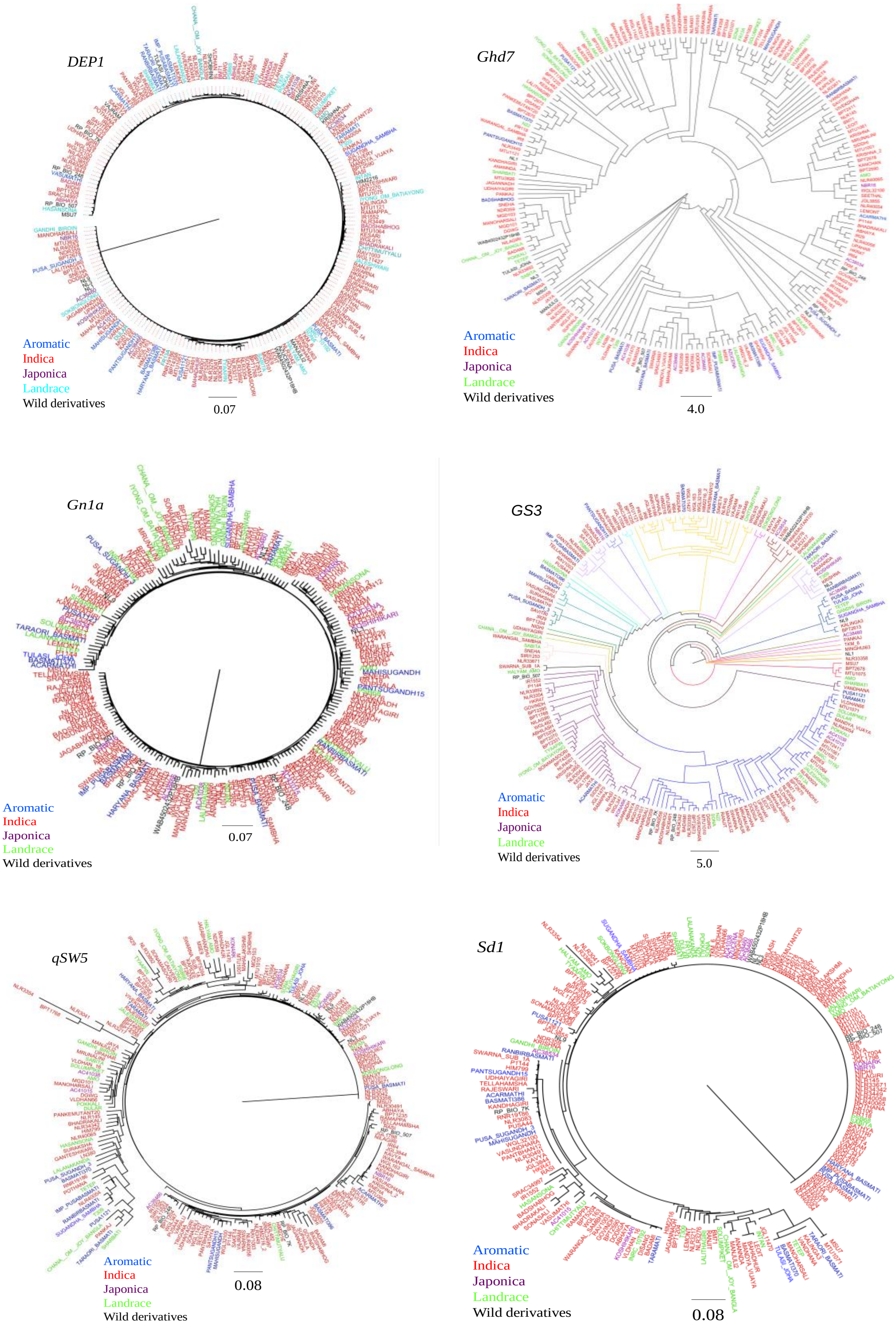

Supplementary Fig. S3 Comparison of mean values of the haplotypes of *Ghd7* (A) and *DEP1* (B) genes for number of panicles for at ARS, Nellore.

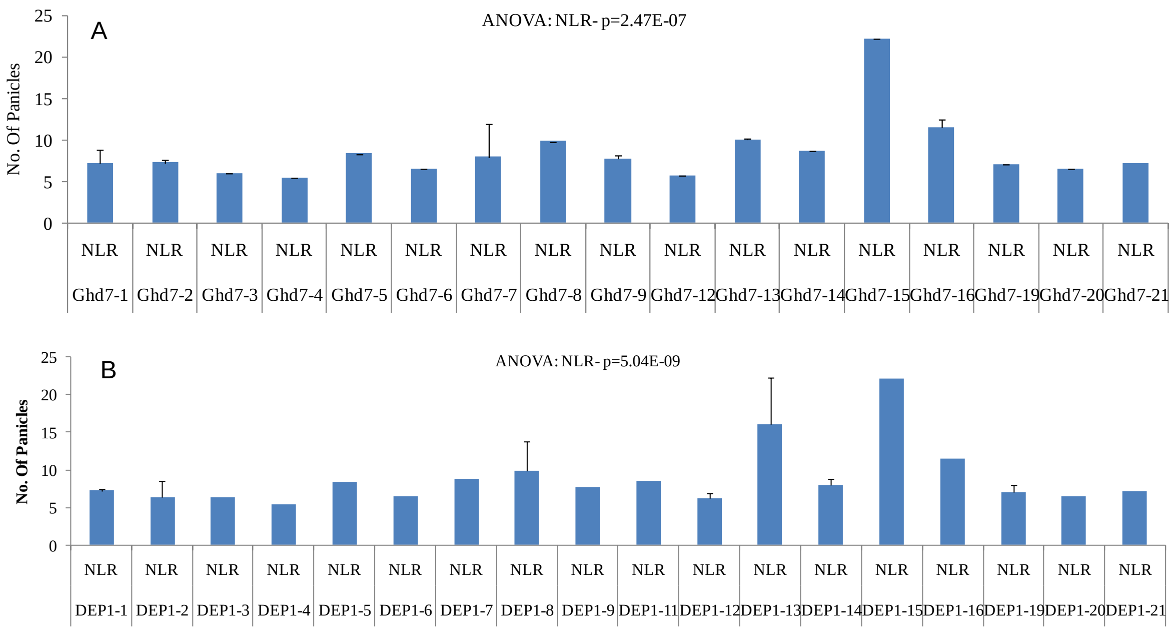

Supplementary Table S1 Summary of morphological traits recorded from the rice genotypes in ARS, Nellore and RARS, Maruteru

Location	Trait	Average	Min.	Max.	SD	CV (%)
ARS, Nellore	Plant height (cm)	90.04	66.00	136.97	11.50	12.77
	Culm height (cm)	73.08	50.86	123.52	11.43	15.63
	No. of Panicles	7.38	2.82	22.22	2.35	31.81
	Panicle length (cm)	23.19	18.67	28.20	1.89	8.16
	Filled grains (No.)	94.95	2.74	216.92	35.32	37.20
	Chaffy grains (No.)	28.99	13.66	77.36	16.31	56.27
	Spikelets per panicle (No.)	123.95	22.60	256.90	41.02	33.10
	Spikelet fertility (%)	76.98	46.78	100.16	10.37	13.47
	Economic yield (g)	9.37	1.21	19.51	3.71	39.64
	Biological yield (g)	26.18	8.20	45.21	7.63	29.13
	Harvest Index	36.24	9.68	90.42	12.47	34.41
	Seed width (mm)	1.88	0.70	2.80	0.44	23.33
	Grain length (mm)	8.63	6.88	12.03	0.99	11.44
	Grain weight (g)	23.45	17.70	31.20	3.23	13.78
RARS, Maruteru	Plant height (cm)	109.77	84.60	162.00	17.02	15.51
	No. f Panicles	8.68	3.30	17.50	2.61	30.06
	Panicle length (cm)	24.45	17.80	36.50	3.31	13.53
	Filled grains (No.)	127.09	104.00	251.30	24.11	18.97
	Chaffy grains (No.)	9.37	0.00	17.50	2.40	25.59
	Spikelets per panicle (No.)	136.44	111.50	265.30	24.74	18.14
	Spikelet fertility (%)	93.03	88.20	96.35	1.61	1.73
	Economic yield (g)	19.35	5.50	45.60	7.09	36.67
	Grain weight (g)	18.01	12.20	27.60	2.64	14.66
	Days to 50% flowering	66.25	49.00	80.50	7.16	10.80
	Spikelet sterility (%)	6.92	0.00	11.80	1.74	25.19

Min.- Minimum, Max. –Maximum, SD- Standard Deviation, CV – Co-Efficient of Variation.

Supplementary Table S2 Correlation analysis of important yield and its contributing traits recorded from the rice accessions in ARS, Nellore and RARS, Maruteru

ARS, Nellore

Trait	PH	CH	NP	PL	FG	CG	SP	SF	EY	BY	HI	GW	GL	GW
PH	1													
CH	0.958**	1												
NP	0.085	0.06	1											
PL	0.459**	0.322**	0	1										
FG	-0.06	-0.03	-0.211**	0.046	1									
CG	0.011	-0.002	-0.232**	0.242**	0.242**	1								
SP	-0.044	-0.025	-0.269**	0.139	0.914**	0.615**	1							
SF	-0.077	-0.043	0.137	-0.250**	0.277**	-0.831**	-0.123	1						
EY	0.088	0.121	0.350**	0.056	0.339**	-0.198*	0.192*	0.405**	1					
BY	0.233**	0.215**	0.441**	0.271**	0.122	0.038	0.115	0.019	0.486**	1				
HI	-0.167*	-0.118	-0.016	-0.175*	0.234**	-0.258**	0.082	0.420**	0.616**	-0.350**	1			
SW	0.061	0.103	-0.115	0.005	-0.141	-0.211**	-0.203*	0.143	0.161*	-0.07	0.259**	1		
GL	0.205*	0.136	-0.177*	0.312**	-0.355**	-0.129	-0.342**	-0.083	-0.156	-0.180*	-0.022	0.354**	1	
GW	-0.102	-0.071	0.022	-0.11	-0.165*	-0.183*	-0.210**	0.08	0.058	0.104	-0.011	0.194*	-0.066	1

RARS, Maruteru

Trait	PH	NP	PL	CG	FG	SP	GW	SS	SF	DFF	EY
PH	1.000										
NP	0.129	1.000									
PL	0.251***	0.146*	1.000								
CG	0.015	0.0279	-0.069	1.000							
FG	-0.065	0.09	-0.023	0.183**	1.000						
SP	-0.056	0.092	-0.032	0.284***	0.994***	1.000					
GWT	0.139 *	0.043	0.134*	-0.076	0.207**	-0.209**	1.000				
SS	0.114	0.0114	0.003	0.430***	-0.416***	-0.359**	0.034	1.000			
SF	-0.0989	-0.0196	0.0030	-0.386 ***	0.426 ***	0.373 ***	-0.017	-0.986 ***	1.000		
DFF	-0.113	-0.059	0.157 *	-0.114	0.104	0.089	-0.0259	-0.069	0.0606	1.000	
EY	0.089	0.819***	-0.079	0.089	0.576***	0.478***	0.154*	-0.152*	0.158*	-0.039	1.000

*** Significance at 0.01% level, **Significance at 1% level, *Significance at 5% level; PH- Plant Height, CH-Culm Height, NP-Number of Panicles, PL-Panicle length, FG-Filled Grains, CG-Chaffy Grains, SP-Spikelets per panicle, SF-Spikelet Fertility, EY-Economic Yield, BY-Biological Yield, HI-Harvest Index, SW-Seed Weight, GL-Grain Length, GW-Grain Width

Supplementary Table S3 Population specific alleles and their corresponding SNP markers. (Markers in italics are novel)

Group	Group specific alleles	Novel alleles	SNPs	*Indels*
DEP1				
Aromatic	2	1	*C9.16412717*	C9.16413214
Indica	24	4	C9.16410224,C9.16410358, C9.16411021, C9.16411269, C9.16411370, C9.16411374, C9.16411375, C9.16412712, C9.16413734, C9.16413742,C9.16413743, C9.16413744, C9.16414264, C9.16414271, C9.16414595, C9.16414599, C9.16415874, C9.16415877, C9.16415878, C9.16415881	*C9.16411273*,*C9.16413744*,*C9.16414274*, *C9.16414604* *C9.16413744*
Japonica	1	1	*C9.16415873*	-
Landrace	6	2	*C9.16410603*, *C9.16410648*	*C9.16411273*,*C9.16413215*,*C9.16413744*, *C9.16415868*
Wild derivatives	4	4	*C9.16411051*, *C9.16415873*	*C9.16413726*, *C9.16413744*
Ghd7				
Aromatic	3	2	C7.9155925	*C7.9154960*,*C7.9154961*
Indica	6	2	*C7.9153979* , *C7.9154953*	*C7.9153974*,*C7.9154956*,*C7.9154960*,*C7.9154961*
Japonica	2	1	*C7.9154955*	*C7.9154961*
Landrace	6	4	*C7.9153975*, *C7.9156081* , *C7.9156180*, *C7.9156228*	*C7.9154961*,*C7.9155399*
Wild derivatives	1	0	C7.9156092	- *C7.9154961*
Gn1a				
Aromatic	4	3	C1.5273008	*C1.5272045*,C1.5272520,*C1.5275527*
Indica	21	16	*C1.5272037*, *C1.5272529*, *C1.5272857*, *C1.5273008*, *C1.5273339*, *C1.5273491*, *C1.5273838*, *C1.5273851*, *C1.5275543*, *C1.5275544*, *C1.5276225*	*C1.5272043*,*C1.5272042*,*C1.5272044*,*C1.5272045*,*C1.5272518*,C1.5272520,C1.5272759,C1.5273847,*C1.5275538*,*C1.5275539*
Japonica	2	1	*C1.5273850*	C1.5272520
Landrace	4	4	*C1.5272523*, *C1.5272917*, *C1.5273838*, *C1.5273849*	-

Wild derivatives	3	1	C1.5276405	*C1.5272513*,C1.5272520
GS3				
Aromatic	1		C3.16730523	-
Indica	15		C3.16730524, C3.16731103, C3.16732255, C3.16732310, C3.16733200, C3.16733450, C3.16733451, C3.16733453, C3.16733454, C3.16733459, C3.16733637, C3.16733872, C3.16734488, C3.16734490, C3.16734492	-
Japonica	0		-	-
Landrace	1		C3.16732258	-
Wild derivatives	1		C3.16734486	-
qSW5				
Aromatic	3	2		*C5.5363982*,C5.5364772,*C5.5365233*
Indica	9	6	*C5.5363971*, *C5.5363976*, *C5.5364757*, *C5.5364760*, *C5.5364766*, *C5.5364767*	*C5.5363980*,*C5.5363982*,*C5.5364770*
Japonica	1	1	-	*C5.5364770*
Landrace	2	1	*C5.5363972*	C5.5364607
Wild derivatives	0	0	-	-
sd1				
Aromatic	0	0	-	-
Indica	4	4	*C1.38382433*, *C1.38382434*, *C1.38382435*	*C1.38382394*
Japonica	1	1	C1.38382165	
Landrace	0	0	-	-
Wild derivatives	1	1	C1.38382165	-

Supplementary Table S4 The marker-trait associations identified in ARS, Nellore and RARS, Maruteru

ARS, Nellore

Gene	Trait	SNP	p	PVE (%)	Method
DEP1	CG	C9.16415032	3.62E-04	11.124	GLM-PCA
	CH	C9.16413000	1.14E-04	14.845	GLM-PCA, GLM-Q, MLM-PCA+K, MLM-Q+K
	CH	C9.16412063	0.00406	8.735	MLM-PCA+K, MLM-Q+K
	CH	C9.16412161	0.00406	8.735	MLM-PCA+K, MLM-Q+K
	EY	C9.16415871	0.00691	9.495	MLM-PCA+K
	EY	C9.16413744	0.01153	5.466	MLM-PCA+K
	FG	C9.16415254	3.69E-04	10.785	GLM-PCA
	GL	C9.16414399	7.59E-06	16.156	GLM-PCA, GLM-Q
	GL	C9.16413000	6.06E-04	10.521	GLM-PCA, GLM-Q
	GL	C9.16414429	0.00804	6.378	GLM-PCA, GLM-Q, MLM-Q+K
	GL	C9.16414739	0.00804	6.378	GLM-PCA, GLM-Q, MLM-Q+K
	GL	C9.16415724	0.0028	7.842	GLM-PCA, GLM-Q, MLM-Q+K
	GL	C9.16414028	1.02E-05	17.142	GLM-PCA, GLM-Q
	GL	C9.16415391	0.00694	6.58	GLM-PCA, GLM-Q, MLM-Q+K
	GL	C9.16413779	9.60E-04	10.788	GLM-Q
	PH	C9.16413000	6.23E-04	12.033	GLM-PCA, GLM-Q, MLM-PCA+K, MLM-Q+K
	PH	C9.16412063	0.00395	8.872	MLM-PCA+K, MLM-Q+K
	PH	C9.16412161	0.00395	8.872	MLM-PCA+K, MLM-Q+K
	PL	C9.16415878	0.01034	5.975	MLM-Q+K
	SP	C9.16415254	3.45E-06	16.131	GLM-PCA, GLM-Q
	SP	C9.16415032	6.21E-04	10.023	GLM-PCA, GLM-Q
	SW	C9.16413275	0.00624	7.85	MLM-Q+K
	SW	C9.16414600	0.00699	5.581	MLM-Q+K
	SW	C9.16414602	0.00699	5.581	MLM-Q+K
Ghd7	NP	C7.9156230	1.12E-07	21.893	GLM-PCA, GLM-Q
	NP	C7.9156258	1.12E-07	21.893	GLM-PCA, GLM-Q
	NP	C7.9156081	1.08E-05	16.317	GLM-PCA, GLM-Q
	NP	C7.9155412	1.17E-04	13.16	GLM-PCA, GLM-Q
	NP	C7.9155431	1.17E-04	13.16	GLM-PCA, GLM-Q
	NP	C7.9155896	1.08E-04	13.269	GLM-PCA, GLM-Q
	NP	C7.9153042	0.00163	10.102	GLM-PCA, MLM-PCA+K
	NP	C7.9153642	1.14E-04	13.469	GLM-Q, MLM-PCA+K, MLM-Q+K
Gn1a	CG	C1.5273339	0.00111	7.846	GLM-PCA
	HI	C1.5273127	0.00446	9.671	GLM-PCA, GLM-Q, MLM-PCA+K, MLM-Q+K
	HI	C1.5272917	0.00542	8.365	MLM-Q+K
	SP	C1.5276405	0.00165	9.666	GLM-PCA
GS3	BY	C3.16733451	5.87E-04	10.756	GLM-Q
	BY	C3.16733453	9.63E-04	10.179	GLM-Q
	EY	C3.16731162	5.19E-04	10.936	GLM-Q
	FG	C3.16733451	6.20E-04	12.309	GLM-PCA, GLM-Q, MLM-PCA+K, MLM-Q+K
	GL	C3.16732887	0.00129	5.802	GLM-Q
	GL	C3.16734121	0.00173	9.928	MLM-PCA+K, MLM-Q+K
	HI	C3.16733451	3.12E-04	12.131	GLM-Q
	HI	C3.16733453	8.66E-04	10.668	GLM-Q
	HI	C3.16733441	1.50E-05	13.313	GLM-Q
	HI	C3.16734492	2.43E-05	12.981	GLM-Q
	HI	C3.16734490	2.78E-05	13.711	GLM-Q
	HI	C3.16734488	2.93E-05	13.434	GLM-Q
	HI	C3.16734484	2.31E-04	11.827	GLM-Q
	HI	C3.16734486	3.15E-04	10.52	GLM-Q
	HI	C3.16734489	3.65E-04	10.342	GLM-Q
	HI	C3.16734487	0.00167	7.986	GLM-Q
	NP	C3.16731567	6.19E-04	9.771	GLM-PCA, MLM-PCA+K
	NP	C3.16733453	0.00155	8.68	GLM-PCA, GLM-Q
	NP	C3.16731566	6.33E-04	8.029	GLM-PCA
	SP	C3.16733451	7.01E-04	10.178	GLM-Q
Qsw5	GW	C5.5365236	4.74E-04	6.729	GLM-Q
	GW	C5.5365234	8.26E-04	6.003	GLM-Q
	GW	C5.5364388	0.01074	6	MLM-PCA+K

	HI	C5.5365234	0.00108	7.604	GLM-Q
Sd1	NP	C1.38381676	3.04E-04	11.655	GLM-PCA, GLM-Q

RARS, Maruteru

Gene	Trait	SNP	p	PVE (%)	Method
DEP1	FG	C9.16411020	0.0011	22.877	GLM-PCA, GLM-Q, MLM-PCA+K, MLM-Q+K
	FG	C9.16413000	0.00102	14.548	GLM-PCA, GLM-Q, MLM-PCA+K, MLM-Q+K
	FG	C9.16412862	0.00111	14.348	GLM-PCA, GLM-Q, MLM-PCA+K, MLM-Q+K
	SP	C9.16411020	0.00126	22.529	GLM-PCA, GLM-Q, MLM-Q+K
	SP	C9.16413000	0.00184	13.383	GLM-PCA, GLM-Q, MLM-PCA+K
	SP	C9.16412862	0.00194	13.268	GLM-PCA, GLM-Q, MLM-PCA+K
GHD7	EY	C7.9156081	4.90E-04	14.752	GLM-PCA
	FG	C7.9153042	8.54E-05	17.579	GLM-PCA, GLM-Q
	FG	C7.9156081	0.00111	13.09	GLM-Q
	SP	C7.9153042	1.23E-04	16.948	GLM-PCA, GLM-Q
	SP	C7.9156081	0.00173	12.421	GLM-PCA
	SP	C7.9153642	0.00193	12.098	GLM-PCA
Gn1a	NP	C1.5272982	0.00209	12.762	GLM-PCA, MLM-PCA+K
	EY	C1.5273850	0.00332	11.005	GLM-PCA, GLM-Q
	GW	C1.5272524	3.96E-04	11.893	GLM-PCA, GLM-Q, MLM-PCA+K, MLM-Q+K
	NP	C1.5272220	1.58E-04	14.897	GLM-PCA
	NP	C1.5273012	1.58E-04	14.897	GLM-PCA
	NP	C1.5273007	2.14E-04	14.428	GLM-PCA
	NP	C1.5276405	0.00494	9.609	GLM-PCA
	NP	C1.5276225	0.00463	12.125	GLM-Q
	PH	C1.5273851	0.00545	17.286	GLM-PCA, MLM-PCA+K
	PH	C1.5276521	0.00132	11.938	GLM-PCA
	PH	C1.5276405	0.00417	10.074	GLM-PCA
GS3	CG	C3.16732765	7.19E-05	17.577	GLM-PCA
	FG	C3.16732255	8.53E-04	10.319	GLM-Q
	FG	C3.16733454	0.00142	9.978	GLM-Q
	SP	C3.16732255	9.02E-04	10.233	GLM-Q
	SP	C3.16733454	0.00149	9.918	GLM-Q
	SS	C3.16732765	6.50E-04	15.677	GLM-PCA, MLM-PCA+K
	SS	C3.16733451	0.00125	12.868	GLM-Q
qSW5	PL	C5.5363979	9.57E-04	20.863	GLM-PCA, GLM-Q

Supplementary Table 5 Haplotype analysis of Ghd7 gene

Haplotype	A1	A2	A3	A4	A5	A6	A7	A8	A9	A10	A11	A12	A13	A14	A15	A16	A17	A18	A19	A20	A21	A22	A23	A24	A25	A26	A27	A28	A29	A30	A31	A32	A33	A34	A35	A36	A37	A38	A39	A40	A41	A42	A43	A44	A45	A46	A47	Aromatic	Indica	Japonica	Landrace	Wild derivatives
Ghd7_14	C	C	A	A	G	G	C	C	C	C	G	T	C	C	T	T	T	T	A	A	C	C	G	G	T	T	C	C	G	G	A	A	C	C	C	C	G	G	C	C	G	G	A	A	C	C	C	0	1	0	1	0
Ghd7_6	G	G	A	A	G	G	C	C	C	C	G	T	C	C	C	C	T	T	A	A	C	C	A	C	T	T	C	C	G	G	A	T	T	T	C	C	G	G	C	A	T	T	A	A	A	A	C	0	0	0	1	0
Ghd7_1	G	G	A	A	G	G	C	C	C	C	G	T	C	C	C	C	T	T	A	A	C	C	A	A	T	T	C	C	G	G	A	A	T	T	C	C	G	G	C	C	T	T	A	A	A	A	C	11	104	8	18	5
Ghd7_11	C	C	A	A	G	G	A	A	G	G	G	T	C	C	C	C	T	T	A	A	C	C	G	G	T	T	C	C	G	G	A	A	T	T	C	C	A	A	C	C	G	G	A	A	C	C	C	1	0	0	1	0
Ghd7_2	G	G	A	A	G	G	C	C	C	C	G	T	C	C	C	C	T	T	A	A	A	A	A	A	T	T	C	C	G	G	A	A	T	T	C	C	G	G	C	C	T	T	A	A	A	A	C	1	3	0	0	0
Ghd7_4	G	G	A	A	G	G	C	C	C	C	G	T	C	C	C	C	T	T	A	A	C	A	A	A	T	T	C	C	G	G	A	A	T	T	C	C	G	G	C	C	T	T	A	A	A	A	C	0	3	0	0	0
Ghd7_3	C	G	A	A	G	G	C	C	C	C	G	T	C	C	C	C	T	T	A	A	C	C	A	A	T	T	C	C	G	G	A	A	T	T	C	C	G	G	C	C	T	T	A	A	A	A	C	0	3	0	0	0
Ghd7_16	C	G	A	A	G	G	C	C	C	C	G	T	C	C	C	T	T	T	A	A	C	C	A	A	T	T	C	C	G	G	A	A	T	C	C	C	G	G	C	C	G	T	A	A	C	A	C	0	1	0	0	0
Ghd7_18	C	G	G	A	A	G	C	C	C	C	C	T	G	C	C	C	C	T	A	A	C	C	G	A	C	T	C	C	C	G	G	A	T	T	T	C	G	G	T	C	G	T	G	A	C	A	G	0	0	0	1	0
Ghd7_9	C	G	A	A	G	G	C	C	C	C	G	T	C	C	C	C	T	T	A	A	C	C	G	A	T	T	C	C	G	G	A	A	T	T	C	C	G	G	C	C	T	T	A	A	A	A	C	0	1	0	0	0
Ghd7_17	G	G	G	A	G	G	C	C	C	C	C	T	C	C	C	C	T	T	A	A	C	C	A	A	C	T	C	C	G	G	G	A	T	T	T	C	G	G	C	C	T	T	G	A	C	A	C	0	1	0	0	0
Ghd7_12	C	C	A	A	G	G	C	C	C	C	G	T	C	C	C	C	T	T	T	T	C	C	G	G	T	T	G	G	G	G	A	A	T	T	C	C	G	G	C	C	G	G	A	A	C	C	C	0	5	0	0	1
Ghd7_13	C	G	A	A	G	G	C	C	C	C	G	T	C	C	C	C	T	T	A	T	C	C	G	G	T	T	G	G	G	G	A	A	T	T	C	C	G	G	C	C	G	G	A	A	C	C	C	0	1	0	0	0
Ghd7_20	C	C	G	G	G	G	C	C	C	C	G	T	G	G	T	T	C	C	A	A	C	C	G	G	G	G	C	C	G	G	G	G	T	T	C	C	G	G	C	C	C	C	G	G	G	G	C	0	0	0	0	1
Ghd7_5	G	G	A	A	G	G	C	C	C	C	G	T	C	T	C	C	T	T	A	A	C	C	A	A	T	T	C	C	G	G	A	A	T	T	C	C	G	G	C	C	T	T	A	A	A	A	C	1	0	0	0	0
Ghd7_19	C	C	G	G	A	A	C	C	C	C	C	A	G	G	C	C	C	C	A	A	C	C	G	G	C	C	C	C	C	C	G	G	T	T	T	T	G	G	C	C	T	T	G	G	G	G	C	1	1	0	0	0
Ghd7_21	T	T	G	G	A	A	G	G	T	T	C	A	G	G	C	C	C	C	A	A	C	C	G	G	C	C	C	C	C	C	G	G	T	T	T	T	G	G	C	C	T	T	G	G	G	G	C	0	0	0	1	0
Ghd7_7	C	G	A	A	G	G	C	C	C	C	G	T	C	C	C	C	T	T	A	T	C	C	G	A	T	T	C	G	G	G	A	A	T	T	C	C	G	G	C	C	G	T	A	A	C	A	C	0	1	0	0	0
Ghd7_10	C	G	A	A	G	G	C	C	C	C	G	T	C	C	C	T	T	T	A	A	C	C	G	A	T	T	C	C	G	G	A	A	T	T	C	C	G	G	C	C	T	T	A	A	A	A	C	0	1	0	0	0
Ghd7_8	C	G	A	A	G	G	C	C	C	C	G	T	C	C	C	T	T	T	A	A	C	C	G	A	T	T	C	C	G	G	A	A	T	C	C	C	G	G	C	C	G	T	A	A	C	A	C	0	2	0	0	0
Ghd7_15	C	G	A	A	G	G	C	C	C	C	C	T	C	C	C	T	T	T	A	A	C	C	G	A	T	T	C	C	G	G	A	A	T	C	C	C	G	G	C	C	G	T	A	A	C	A	C	0	0	0	1	0

Supplementary Table 6 Haplotype analysis of GS3 gene

Haplotype	A1	A2	A3	A4	A5	A6	A7	A8	A9	A10	A11	A12	A13	A14	A15	A16	A17	A18	A19	A20	A21	A22	A23	A24	A25	A26	A27	A28	A29	A30	A31	A32	A33	A34	A35	A36	A37	A38	A39	A40	A41	A42	A43	A44	A45	A46	A47	A48	A49	A50	A51	A52	A53	A54	A55	A56	A57	A58	A59	A60	A61	A62	A63	A64	A65	A66	A67	A68	A69	A70	A71	A72	A73	A74	A75	A76	A77	A78	A79	A80	A81	A82	A83	A84	A85	A86	A87	A88	A89	Aromatic	Indica	Japonica	Landrace	Wild derivatives	
Hap_47	C	C	C	C	A	G	C	C	G	G	G	G	G	G	G	G	G	T	T	A	A	T	C	T	C	C	C	C	G	T	T	T	T	T	T	G	G	G	G	C	C	T	T	T	T	C	G	G	A	A	C	C	A	A	A	A	A	A	C	C	A	A	T	T	T	T	G	G	A	A	G	G	G	G	T	T	A	A	C	C	G	G	G	G	G	A	A	G	G	0	1	0	0	0	1
Hap_58	C	C	C	C	A	A	C	C	G	G	G	G	G	G	G	G	G	T	T	A	A	T	C	T	C	C	C	C	G	T	T	T	T	T	T	G	G	G	G	C	C	T	T	T	T	C	G	G	A	A	C	C	A	A	A	A	A	A	C	C	A	A	T	T	T	T	G	G	A	A	G	G	G	G	T	T	A	A	C	C	G	C	C	T	T	C	C	T	T	0	1	0	0	0	1
Hap_1	C	C	A	A	G	G	A	A	C	G	G	A	A	G	G	A	A	C	C	A	C	G	G	G	C	A	T	T	A	G	C	C	C	T	T	G	G	T	T	C	C	C	C	A	A	C	T	T	G	G	T	T	C	C	G	G	G	G	T	T	G	G	C	C	G	G	T	T	G	G	A	A	G	G	G	G	G	G	C	C	G	G	G	G	G	A	A	G	G	1	0	0	0	0	1
Hap_7	C	C	A	A	G	G	A	A	C	G	G	A	A	G	G	A	A	C	C	A	C	T	G	G	C	A	T	A	A	G	C	C	C	T	T	G	G	T	T	C	C	C	C	A	A	C	T	T	G	C	T	T	C	C	G	G	G	G	T	T	G	G	C	C	G	G	T	T	G	G	A	A	G	G	G	G	G	G	C	C	G	G	G	G	G	A	A	G	G	0	1	0	0	0	1
Hap_21	C	C	A	A	G	G	A	A	C	G	G	A	A	G	G	A	A	C	C	A	C	T	G	T	C	A	C	A	A	G	C	C	C	T	T	G	G	T	T	C	C	C	C	A	A	C	T	T	G	G	T	T	C	C	G	G	G	G	T	T	G	G	C	C	G	G	T	T	G	G	A	A	G	G	G	G	G	G	C	C	G	G	G	G	G	A	A	G	G	0	1	0	0	0	1
Hap_9	C	C	A	A	G	G	A	A	C	G	G	A	A	G	G	A	A	C	C	A	A	G	G	G	C	A	T	T	A	G	C	C	C	T	T	G	G	T	T	C	C	C	C	A	A	C	T	T	G	G	T	T	C	C	G	G	G	G	T	T	G	G	C	C	G	G	T	T	G	G	A	A	G	G	G	G	G	G	C	C	G	G	G	G	G	A	A	G	G	0	0	1	0	0	1
Hap_76	C	C	C	C	G	G	A	A	C	A	A	G	G	A	A	G	G	C	C	C	C	T	G	T	A	A	C	C	G	T	C	C	C	T	T	T	T	G	G	C	C	C	C	T	T	C	G	G	A	A	C	C	A	C	A	G	A	G	C	C	A	G	T	C	T	G	G	T	A	A	G	A	G	G	T	G	A	G	C	C	T	G	G	G	G	A	A	G	G	0	0	0	1	0	1
Hap_107	T	T	A	A	G	G	A	A	C	A	A	G	G	A	A	G	G	C	C	A	A	T	C	T	C	C	C	C	G	T	C	T	C	T	T	G	G	G	T	C	C	T	C	T	T	T	G	G	G	G	T	T	C	C	G	G	G	G	C	C	G	G	C	C	G	G	T	T	A	A	A	A	A	A	G	G	G	G	C	C	G	G	G	G	G	A	A	G	G	0	0	0	1	0	1
Hap_106	T	T	A	A	G	G	A	A	C	A	A	G	G	A	A	G	G	C	C	A	A	T	C	T	C	C	C	C	G	T	C	T	C	T	A	G	T	G	T	C	T	T	C	T	T	T	G	G	G	G	T	T	C	C	G	G	G	G	C	C	G	G	C	C	G	G	T	T	A	A	A	A	A	A	G	G	G	G	C	C	G	G	G	G	G	A	A	G	G	0	1	0	0	0	1
Hap_102	T	T	A	A	G	G	A	A	C	A	A	G	G	A	A	G	G	C	C	A	A	T	C	T	C	A	C	C	A	T	C	C	C	T	A	G	T	T	T	C	T	C	C	T	T	C	G	G	G	G	T	T	C	C	G	G	G	G	C	C	G	G	C	C	G	G	T	T	A	A	A	A	A	A	G	G	G	G	C	C	G	G	G	G	G	A	A	T	T	1	0	0	0	0	1
Hap_72	T	T	A	A	G	G	A	A	C	A	A	G	G	A	A	G	G	C	C	A	C	T	G	G	C	A	T	T	A	G	C	C	C	A	A	T	T	T	T	T	T	T	C	T	T	C	G	G	A	A	C	C	A	A	A	A	A	A	C	C	A	A	T	T	T	T	G	G	A	A	G	G	G	G	T	T	A	A	C	C	G	G	C	G	T	A	C	G	T	0	1	0	0	0	1
Hap_73	T	T	A	A	G	G	A	A	C	A	A	G	G	A	A	G	G	C	C	A	C	T	G	T	C	A	C	C	A	G	C	C	C	A	A	T	T	T	T	T	T	T	C	T	T	C	G	G	A	A	C	C	A	A	A	A	A	A	C	C	A	A	T	T	T	T	G	G	A	A	G	G	G	G	T	T	A	A	C	C	G	G	C	G	T	A	C	G	T	0	1	0	0	0	1
Hap_98	T	T	A	A	G	G	A	A	C	A	A	G	G	A	A	G	G	C	C	C	C	T	G	G	C	A	T	A	A	G	C	C	C	A	A	T	T	T	T	T	T	C	C	T	T	T	G	G	G	G	T	T	C	C	G	G	G	G	C	C	G	G	C	C	G	G	T	T	A	A	A	A	A	A	G	G	G	G	C	C	G	G	G	G	G	A	A	G	G	0	1	0	0	0	1
Hap_99	T	T	A	A	G	G	A	A	C	A	A	G	G	A	A	G	G	C	C	A	C	T	G	G	C	A	T	T	A	G	C	C	C	A	A	T	T	T	T	T	T	C	C	T	T	T	G	G	G	G	T	T	C	C	G	G	G	G	C	C	G	G	C	C	G	G	T	T	A	A	A	A	A	A	G	G	G	G	C	C	G	G	G	G	G	A	A	G	G	0	1	0	0	0	1
Hap_101	T	T	A	A	G	G	A	A	C	A	A	G	G	A	A	G	G	C	C	A	C	T	G	T	C	A	C	A	A	G	C	C	C	A	A	T	T	T	T	T	T	C	C	T	T	T	G	G	G	G	T	T	C	C	G	G	G	G	C	C	G	G	C	C	G	G	T	T	A	A	A	A	A	A	G	G	G	G	C	C	G	G	G	G	G	A	A	G	G	0	0	0	0	1	1
Hap_103	T	T	A	A	G	G	A	A	C	A	A	G	G	A	A	G	G	C	C	A	A	G	G	T	C	A	C	T	A	G	C	T	C	T	A	G	T	G	T	C	T	C	C	T	T	T	G	G	G	G	T	T	C	C	G	G	G	G	C	C	G	G	C	C	G	G	T	T	A	A	A	A	A	A	G	G	G	G	C	C	G	G	G	G	G	A	A	G	G	0	1	0	0	0	1
Hap_104	T	T	A	A	G	G	A	A	C	A	A	G	G	A	A	G	G	C	C	A	C	T	G	T	C	A	T	C	A	G	C	T	C	T	A	G	T	G	T	C	T	C	C	T	T	T	G	G	G	G	T	T	C	C	G	G	G	G	C	C	G	G	C	C	G	G	T	T	A	A	A	A	A	A	G	G	G	G	C	C	G	G	G	G	G	A	A	G	G	0	1	0	0	0	1
Hap_2	C	C	A	A	G	G	A	A	C	G	G	A	A	G	G	A	A	C	C	A	C	T	G	G	C	A	T	T	A	G	C	C	C	T	T	G	G	T	T	C	C	C	C	A	A	C	T	T	G	G	T	T	C	C	G	G	G	G	T	T	G	G	C	C	G	G	T	T	G	G	A	A	G	G	G	G	G	G	C	C	G	G	G	G	G	A	A	G	G	0	3	1	1	0	5
Hap_33	C	C	A	A	G	G	A	A	C	G	G	A	A	G	G	A	A	C	C	A	A	T	C	T	C	A	C	C	A	T	C	T	C	T	T	G	G	G	T	C	C	C	C	A	A	C	T	T	G	G	T	T	C	C	G	G	G	G	T	T	G	G	C	C	G	G	T	T	G	G	A	A	G	G	G	G	G	G	C	C	G	G	G	G	G	A	A	G	G	0	1	0	0	0	1
Hap_16	C	C	A	A	G	G	A	A	C	G	G	A	A	G	G	A	A	C	C	A	C	T	C	T	C	A	C	C	A	G	C	C	C	T	T	G	G	T	T	C	C	C	C	A	A	C	T	T	G	G	T	T	C	C	G	G	G	G	T	T	G	G	C	C	G	G	T	T	G	G	A	A	G	G	G	G	G	G	C	C	G	G	G	G	T	A	A	G	T	1	0	0	0	0	1
Hap_86	C	C	C	A	A	G	C	A	C	G	G	G	A	G	G	G	A	T	C	A	C	T	C	T	C	A	C	C	A	T	C	C	C	T	T	G	G	T	T	C	C	C	C	A	A	C	T	T	G	G	T	T	C	C	G	G	G	G	T	T	G	G	C	C	G	G	T	T	G	G	A	A	G	G	G	G	G	G	C	C	G	G	G	G	T	A	A	G	T	0	1	0	0	0	1
Hap_25	C	C	A	A	G	G	A	A	C	G	G	A	A	G	G	A	A	C	C	A	A	T	C	T	C	A	C	C	A	T	C	C	C	T	T	G	G	T	T	C	C	C	C	A	A	C	T	T	G	G	T	T	C	C	G	G	G	G	T	T	G	G	C	C	G	G	T	T	G	G	A	A	G	G	G	G	G	G	C	C	G	G	G	G	G	A	A	G	G	0	1	0	0	0	1
Hap_26	C	T	A	A	G	G	A	A	C	G	A	G	A	G	A	G	A	C	C	A	A	T	C	T	C	A	C	C	A	T	C	C	C	T	T	G	G	T	T	C	C	C	C	A	A	C	T	T	G	G	T	T	C	C	G	G	G	G	T	T	G	G	C	C	G	G	T	T	G	G	A	A	G	A	G	G	G	G	C	C	G	G	G	G	G	A	A	G	G	0	1	0	0	0	1
Hap_28	C	C	A	A	A	G	C	A	C	G	G	A	A	G	G	G	A	T	C	A	A	T	C	T	C	A	C	C	G	T	C	C	C	T	T	G	G	T	T	C	C	C	C	A	A	C	T	T	G	G	T	T	C	C	G	G	G	G	T	T	G	G	C	C	G	G	T	T	G	G	A	A	G	G	G	G	G	G	C	C	G	G	G	G	T	A	A	G	T	0	1	0	0	0	1
Hap_22	C	C	A	A	G	G	A	A	C	G	G	A	A	G	G	A	A	C	C	A	A	T	C	T	C	A	C	C	G	T	C	C	C	T	T	G	G	T	T	C	C	C	C	A	A	C	T	T	G	G	T	T	C	C	G	G	G	G	T	T	G	G	C	C	G	G	T	T	G	G	A	A	G	G	G	G	G	G	C	C	T	G	G	G	G	A	A	G	G	0	0	0	1	0	1
Hap_96	C	C	C	A	G	G	A	A	C	G	G	G	A	G	G	G	A	C	C	A	A	T	C	T	C	A	C	C	G	T	C	T	C	T	T	G	G	G	T	C	C	C	C	T	A	C	G	T	G	G	C	T	A	C	G	G	G	G	C	T	G	G	C	C	T	G	G	T	G	G	G	A	G	G	G	G	G	G	C	C	G	G	G	G	G	A	A	G	G	0	0	0	0	1	1
Hap_38	C	C	A	A	G	G	A	A	C	G	G	A	A	G	G	A	A	C	C	A	C	T	C	T	C	A	T	C	A	G	C	T	C	T	T	G	G	G	T	C	C	C	C	A	A	C	T	T	G	G	T	T	C	C	G	G	G	G	T	T	G	G	C	C	G	G	T	T	G	G	A	A	G	G	G	G	G	G	C	C	G	G	G	G	G	A	A	G	G	0	1	0	0	0	1
Hap_40	C	C	A	A	G	G	A	A	C	G	G	G	A	G	G	G	A	C	C	A	A	T	C	G	C	A	C	C	A	G	C	T	C	T	T	G	G	G	T	C	C	C	C	A	A	C	T	T	G	G	T	T	C	C	G	G	G	G	T	T	G	G	C	C	G	G	T	T	G	G	A	A	G	G	G	G	G	G	C	C	G	G	G	G	G	A	A	G	G	0	1	0	0	0	1
Hap_19	C	C	A	A	G	G	A	A	C	G	G	A	A	G	G	A	A	C	C	A	A	T	G	G	C	A	T	T	A	G	C	C	C	T	T	G	G	T	T	C	C	C	C	A	A	C	T	T	G	G	T	T	C	C	G	G	G	G	T	T	G	G	C	C	G	G	T	T	G	G	A	A	G	G	G	G	G	G	C	C	G	G	G	G	G	A	A	G	G	0	1	0	0	0	1
Hap_77	C	C	C	C	A	A	C	C	C	G	G	G	A	G	G	G	A	T	C	A	A	T	C	T	C	C	C	C	G	T	C	T	C	T	T	G	G	G	T	C	C	T	C	T	A	C	G	T	A	G	C	T	A	C	A	G	A	G	C	T	A	G	T	C	T	G	G	T	A	G	G	A	G	G	T	T	A	G	C	C	G	G	G	G	G	A	A	G	G	0	1	0	0	0	1
Hap_88	C	C	A	A	G	G	A	A	C	G	G	A	A	G	G	A	A	C	C	C	C	T	C	T	A	A	C	C	G	T	C	C	C	T	T	G	G	T	T	C	C	C	C	A	A	C	G	T	A	G	C	T	A	C	A	G	A	G	C	T	A	G	T	C	T	G	G	T	A	G	G	A	G	G	T	G	A	G	C	C	G	G	G	G	G	A	A	G	G	0	1	0	0	0	1
Hap_4	C	C	A	A	G	G	A	A	C	G	G	A	A	G	G	A	A	C	C	A	A	G	G	G	C	A	C	T	A	G	C	C	C	T	T	G	G	T	T	C	C	C	C	A	A	C	T	T	G	G	T	T	C	C	G	G	G	G	T	T	G	G	C	C	G	G	T	T	G	G	A	A	G	G	G	G	G	G	C	C	G	G	A	G	G	A	A	G	T	0	1	0	0	0	1
Hap_92	C	C	A	A	G	G	A	A	C	G	G	A	A	G	G	A	A	C	C	A	C	T	G	T	C	A	C	T	A	G	C	C	C	T	T	G	G	T	T	C	C	T	C	T	A	C	G	T	A	G	C	T	A	C	A	G	A	G	C	T	A	G	T	C	T	G	G	T	A	G	G	A	G	G	T	T	A	A	C	C	G	G	G	G	G	A	A	G	G	0	0	0	1	0	1
Hap_90	C	C	A	A	G	G	A	A	C	G	G	A	A	G	G	A	A	C	C	A	C	T	G	G	C	A	T	T	A	G	C	C	C	T	T	G	G	T	T	C	C	T	C	T	A	C	G	T	A	G	C	T	A	C	A	G	A	G	C	T	A	G	T	C	T	G	G	T	A	G	G	A	G	G	T	G	A	G	C	C	G	G	G	G	G	A	A	G	G	0	2	0	0	0	2
Hap_95	C	C	C	C	G	G	A	A	C	A	A	G	G	A	A	G	G	C	C	A	C	T	G	G	C	A	T	T	A	G	C	C	C	T	T	T	T	T	T	C	C	T	C	T	A	C	G	T	A	G	C	T	A	C	G	G	A	G	C	T	A	G	T	C	T	G	G	T	A	G	G	A	G	G	T	G	A	G	C	C	G	G	G	G	G	A	A	G	G	0	1	0	0	0	1
Hap_91	C	C	A	A	G	G	A	A	C	G	G	A	A	G	G	A	A	C	C	A	A	G	G	G	C	A	T	T	A	G	C	C	C	T	T	G	G	T	T	C	C	T	C	T	A	C	G	T	A	G	C	T	A	C	A	G	A	G	C	T	A	G	T	C	T	G	G	T	A	G	G	A	G	G	T	G	A	G	C	C	G	G	G	G	G	A	A	G	G	0	1	0	0	0	1
Hap_93	C	C	A	A	G	G	A	A	C	G	G	A	A	G	G	A	A	C	C	A	A	T	G	T	C	A	C	T	A	G	C	C	C	T	T	G	G	T	T	C	C	T	C	T	A	C	G	T	A	G	C	T	A	C	A	G	A	G	C	T	A	G	T	C	T	G	G	T	A	G	G	A	G	G	T	G	A	G	C	C	G	G	G	G	G	A	A	G	G	0	1	0	0	0	1
Hap_94	C	C	A	A	G	G	A	A	C	G	G	A	A	G	G	A	A	C	C	A	A	T	G	T	C	A	C	T	A	G	C	C	C	T	T	G	G	G	T	C	C	T	C	T	A	C	G	T	A	G	C	T	A	C	A	G	A	G	C	T	A	G	T	C	T	G	G	T	A	G	G	A	G	G	T	G	A	G	C	C	T	G	G	G	G	A	A	G	G	0	1	0	0	0	1
Hap_82	C	C	C	A	A	G	C	A	C	G	G	G	A	G	G	G	A	T	C	A	A	T	C	T	C	C	C	C	G	T	T	T	T	T	T	G	G	G	G	C	C	T	C	T	A	C	G	T	A	G	C	T	A	C	A	G	A	G	C	T	A	G	T	C	T	G	G	T	A	G	G	A	G	G	T	G	A	G	C	C	G	G	G	G	G	A	A	G	G	1	1	0	0	0	2
Hap_83	C	C	C	A	A	G	C	A	C	G	G	G	A	G	G	G	A	T	C	A	A	T	C	T	C	C	C	C	G	T	T	T	T	T	T	G	G	G	T	C	C	T	C	T	A	C	G	T	A	G	C	T	A	C	A	G	A	G	C	T	A	G	T	C	T	G	G	T	A	G	G	A	G	G	T	G	A	G	C	C	G	G	G	G	G	A	A	G	G	0	0	0	0	1	1
Hap_78	C	C	C	A	A	G	C	A	C	G	G	G	A	G	G	G	A	T	C	A	A	T	C	T	C	C	C	C	G	T	C	T	C	T	T	G	G	G	T	C	C	T	C	T	A	C	G	T	A	G	C	T	A	C	A	G	A	G	C	T	A	G	T	C	T	G	G	T	A	G	G	A	G	G	T	G	A	G	C	C	G	G	G	G	G	A	A	G	G	0	2	1	0	0	3
Hap_81	C	C	C	A	A	G	C	A	C	G	G	G	A	G	G	G	A	T	C	A	A	T	C	T	C	C	C	C	G	T	C	T	C	T	T	G	G	G	T	C	C	T	C	T	A	C	G	T	A	G	C	T	A	C	A	G	A	G	C	T	A	G	T	C	T	G	G	T	A	G	G	A	G	G	T	G	A	G	C	C	G	G	C	G	T	C	C	G	T	1	0	0	0	0	1
Hap_89	C	C	A	A	G	G	A	A	C	G	G	A	A	G	G	A	A	C	C	A	A	T	C	T	C	C	C	C	G	T	C	T	C	T	T	G	G	G	T	C	C	T	C	T	A	C	G	T	A	G	C	T	A	C	A	G	A	G	C	T	A	G	T	C	T	G	G	T	A	G	G	A	G	G	T	G	A	G	C	C	G	G	G	G	G	A	A	G	G	0	1	0	0	0	1
Hap_79	C	C	C	A	A	G	C	A	C	G	G	G	A	G	G	G	A	T	C	A	A	T	C	T	C	C	C	C	G	T	C	T	C	T	T	G	G	G	T	C	C	T	C	T	A	C	G	T	A	G	C	T	A	C	A	G	A	G	C	T	A	G	T	C	T	G	G	T	A	G	G	A	G	G	T	G	A	G	C	C	T	G	G	G	G	A	A	G	G	0	1	0	0	0	1
Hap_80	C	C	C	A	A	G	C	A	C	G	G	G	A	G	G	G	A	T	C	A	A	T	C	T	C	C	C	C	G	T	C	T	C	T	T	G	G	G	T	C	C	T	C	T	A	C	G	T	A	G	C	T	A	C	A	G	A	G	C	T	A	G	T	C	T	G	G	T	A	G	G	A	G	G	T	G	A	G	C	C	G	G	G	G	T	A	A	G	T	0	2	0	0	0	2
Hap_69	C	C	C	C	A	A	C	C	G	G	G	G	G	G	G	G	G	T	T	A	A	T	C	T	C	C	C	C	G	T	T	T	T	T	T	G	G	G	G	C	C	T	C	T	A	C	G	T	A	G	C	T	A	C	A	G	A	G	C	T	A	G	T	C	T	G	G	T	A	G	G	A	G	G	T	G	A	G	C	C	G	G	G	G	G	A	A	G	G	0	0	0	1	0	1
Hap_65	C	C	C	C	A	A	C	C	G	G	G	G	G	G	G	G	G	T	T	A	A	T	C	T	C	C	C	C	G	T	T	T	T	T	T	G	G	G	G	C	C	T	C	T	A	C	G	T	A	G	C	T	A	C	A	G	A	G	C	T	A	G	T	C	T	G	G	T	A	G	G	A	G	G	T	G	A	G	C	C	G	G	G	G	T	A	A	G	T	1	1	0	0	0	2
Hap_68	C	C	C	C	A	A	C	C	G	G	G	G	G	G	G	G	G	T	T	A	A	T	C	T	C	C	C	C	G	T	T	T	T	T	T	G	G	G	G	C	C	T	C	T	A	C	G	T	A	G	C	T	A	C	A	G	A	G	C	T	A	G	T	C	T	G	G	T	A	G	G	A	G	G	T	G	A	G	C	C	G	G	G	G	G	A	A	G	T	0	1	0	0	0	1
Hap_85	C	C	C	A	A	G	A	A	C	G	G	G	A	G	G	G	A	T	C	A	A	T	C	T	C	C	C	C	G	T	C	C	C	T	T	G	G	T	T	C	C	C	C	A	A	C	T	T	G	G	T	T	C	C	G	G	G	G	T	T	G	G	C	C	G	G	T	T	G	G	A	A	G	G	G	G	G	G	C	C	G	G	G	G	G	A	A	G	G	0	1	0	0	0	1
Hap_84	C	C	C	A	A	G	C	A	C	G	G	G	A	G	G	G	A	T	C	A	A	T	C	T	C	C	C	C	G	T	C	C	C	T	T	G	G	G	T	C	C	C	C	A	A	C	T	T	G	G	T	T	C	C	G	G	G	G	T	T	G	G	C	C	G	G	T	T	G	G	A	A	G	G	G	G	G	G	C	C	G	G	G	G	G	A	A	G	G	0	1	0	0	0	1
Hap_87	C	C	C	A	A	G	C	A	C	G	G	G	A	G	G	G	A	T	C	A	A	T	C	T	C	C	C	C	G	T	C	C	C	T	T	G	G	G	T	C	C	C	C	T	A	C	T	T	G	G	T	T	C	C	G	G	G	G	T	T	A	G	C	C	G	G	T	T	G	G	A	A	G	G	G	G	G	G	C	C	G	G	G	G	G	A	A	G	G	0	1	0	0	0	1
Hap_20	C	C	A	A	G	G	A	A	C	G	G	A	A	G	G	A	A	C	C	A	A	T	G	G	C	A	T	T	A	G	C	C	C	T	T	G	G	T	T	C	C	C	C	A	A	C	T	T	G	G	T	T	C	C	G	G	G	G	T	T	G	G	C	C	G	G	T	T	G	G	A	A	G	G	G	G	G	G	C	C	G	G	G	G	G	A	A	G	G	0	0	0	1	0	1
Hap_41	C	C	A	A	G	G	A	A	C	G	G	A	A	G	G	A	A	C	C	A	A	T	C	T	C	C	C	C	G	T	C	T	T	T	T	G	G	G	T	C	C	C	C	T	A	C	T	T	G	G	T	T	C	C	G	G	G	G	T	T	G	G	C	C	G	G	T	T	G	G	A	A	G	G	G	G	G	G	C	C	T	G	G	G	G	A	A	G	G	0	1	0	0	0	1
Hap_70	C	T	C	A	A	G	C	A	C	G	A	G	G	G	A	G	G	T	C	A	A	T	C	T	C	C	C	C	G	T	C	T	C	T	A	G	T	G	T	C	T	T	C	T	T	C	G	G	A	G	C	T	A	C	A	G	A	G	C	C	A	G	T	C	T	G	G	T	A	A	G	A	G	A	T	G	A	G	C	C	G	G	C	G	T	A	C	G	T	0	1	0	0	0	1
Hap_71	C	T	C	A	A	G	C	A	C	G	A	G	G	G	A	G	G	T	C	A	A	T	C	T	C	C	C	C	G	T	C	T	C	T	A	G	T	G	T	C	T	T	C	T	T	C	G	T	A	G	C	T	A	C	A	G	A	G	C	T	A	G	T	C	T	G	G	T	A	G	G	A	G	A	T	G	A	G	C	C	G	G	G	G	G	A	A	G	G	0	1	0	0	0	1
Hap_42	C	C	A	A	G	G	A	A	C	G	G	A	A	G	G	A	A	C	C	A	A	T	C	T	C	C	C	C	G	T	C	T	C	T	T	G	G	G	T	C	C	T	C	T	A	C	T	T	G	G	T	T	C	C	G	G	G	G	T	T	G	G	C	C	G	G	T	T	G	G	A	A	G	G	G	G	G	G	C	C	T	G	G	G	G	A	A	G	G	0	1	0	0	0	1
Hap_67	C	C	C	C	A	A	C	C	G	G	G	G	G	G	G	G	G	T	T	A	A	T	C	T	C	C	C	C	G	T	C	T	T	T	T	G	G	G	T	C	C	T	C	T	A	T	G	T	G	G	T	T	C	C	G	G	G	G	C	C	G	G	C	C	G	G	T	T	A	A	A	A	A	A	G	G	G	G	C	C	G	G	C	G	T	A	C	G	T	0	1	0	0	0	1
Hap_27	C	C	A	A	G	G	A	A	C	G	G	A	A	G	G	A	A	C	C	A	A	T	C	T	C	C	C	C	G	T	C	C	C	T	T	G	G	G	T	C	C	C	C	A	A	C	T	T	G	G	T	T	C	C	G	G	G	G	T	T	G	G	C	C	G	G	T	T	G	G	A	A	G	G	G	G	G	G	C	C	G	G	G	G	T	A	A	G	T	0	1	0	0	0	1
Hap_36	C	C	A	A	G	G	A	A	C	G	G	G	A	G	A	G	A	C	C	A	A	T	C	T	C	C	C	C	G	T	T	T	T	T	T	G	G	G	T	C	C	T	C	T	A	C	T	T	G	G	T	T	C	C	G	G	G	G	T	T	G	G	C	C	G	G	T	T	A	G	A	A	G	G	G	G	G	G	C	C	G	G	G	G	G	A	A	G	G	0	1	0	0	0	1
Hap_31	C	C	A	A	G	G	A	A	G	G	G	A	A	G	G	A	A	T	C	A	A	T	C	T	C	C	C	C	G	T	C	T	T	G	G	G	G	T	T	C	C	C	C	A	A	C	T	T	G	G	T	T	C	C	G	G	G	G	T	T	G	G	C	C	G	G	T	T	G	G	A	A	G	G	G	G	G	G	C	C	G	G	G	G	G	A	A	G	G	0	0	0	1	0	1
Hap_64	C	C	C	C	A	A	C	C	G	G	G	G	G	G	G	G	G	T	T	A	A	T	C	T	C	C	C	C	G	T	T	T	T	T	T	G	G	G	G	C	C	T	C	T	T	C	G	G	A	A	C	C	A	C	A	G	A	G	C	C	A	G	T	C	T	G	G	T	A	A	G	A	G	A	T	G	A	G	C	C	G	G	C	G	T	A	C	G	T	0	1	0	0	0	1
Hap_66	C	C	C	C	A	A	C	C	G	G	G	G	G	G	G	G	G	T	T	A	A	T	C	T	C	C	C	C	G	T	T	T	T	T	T	G	G	G	G	C	C	T	C	T	A	C	T	T	G	G	T	T	C	C	G	G	G	G	T	T	G	G	C	C	G	G	T	T	G	G	A	A	G	G	G	G	G	G	C	C	G	G	G	G	T	A	A	G	G	1	0	0	0	0	1
Hap_34	C	C	A	A	G	G	A	A	C	G	G	A	A	G	G	A	A	C	C	A	A	T	C	T	C	C	C	C	G	T	C	T	C	T	T	G	G	G	T	C	C	C	C	A	A	C	T	T	G	G	T	T	C	C	G	G	G	G	T	T	G	G	C	C	G	G	T	T	G	G	A	A	G	G	G	G	G	G	C	C	G	G	G	G	G	A	A	G	G	0	4	0	0	0	4
Hap_30	C	C	A	A	G	G	A	A	C	G	G	A	A	G	G	A	A	C	C	A	A	T	C	T	C	C	C	C	G	T	C	T	C	T	T	G	G	G	T	C	C	C	C	A	A	C	T	T	G	G	T	T	C	C	G	G	G	G	T	T	G	G	C	C	G	G	T	T	G	G	A	A	G	G	G	G	G	G	C	C	T	G	G	G	G	A	A	G	G	0	3	0	0	0	3
Hap_43	C	C	A	A	G	G	A	A	C	G	G	A	A	G	G	A	A	C	C	A	A	T	C	T	C	C	C	C	G	T	C	T	C	T	T	G	G	G	T	C	C	T	C	T	A	C	T	T	G	G	T	T	C	C	G	G	G	G	T	T	G	G	C	C	G	G	T	T	G	G	A	A	G	G	G	G	A	G	C	C	T	G	G	G	G	A	A	G	G	0	1	0	0	0	1
Hap_37	C	C	A	A	G	G	A	A	C	G	G	A	A	G	G	A	A	C	C	A	A	T	C	T	C	C	C	C	G	T	C	T	C	T	T	G	G	G	T	C	C	T	C	T	A	C	T	T	G	G	T	T	C	C	G	G	G	G	T	T	G	G	C	C	G	G	T	T	G	G	A	A	G	G	G	G	G	G	C	C	G	G	G	G	G	A	A	G	G	0	1	0	0	0	1
Hap_29	C	C	A	A	A	A	A	A	C	G	G	A	A	G	G	A	A	C	C	A	A	T	C	T	C	C	C	C	G	T	C	T	C	T	T	G	G	G	T	C	C	T	C	T	A	C	T	T	G	G	T	T	C	C	G	G	G	G	T	T	G	G	C	C	G	G	T	T	G	G	A	A	G	G	G	G	G	G	C	C	T	G	G	G	G	A	A	G	G	0	1	0	0	0	1
Hap_32	C	C	A	A	G	G	A	A	C	G	G	A	A	G	G	A	A	C	C	A	A	T	C	T	C	A	C	C	G	T	C	T	C	T	T	G	G	G	T	C	C	T	C	T	A	C	T	T	G	G	T	T	C	C	G	G	G	G	T	T	G	G	C	C	G	G	T	T	G	G	A	A	G	G	G	G	G	G	C	C	G	G	G	G	G	A	A	G	G	0	1	0	0	0	1
Hap_35	C	C	A	A	G	G	A	A	C	G	G	A	A	G	G	A	A	C	C	A	A	T	C	T	C	A	C	C	G	T	C	T	C	T	T	G	G	G	T	C	C	C	C	A	A	C	T	T	G	G	T	T	C	C	G	G	G	G	T	T	G	G	C	C	G	G	T	T	G	G	A	A	G	G	G	G	G	G	C	C	G	G	G	G	G	A	A	G	G	0	1	0	0	0	1
Hap_75	C	C	A	A	G	G	A	A	C	G	G	A	A	G	G	A	A	C	C	A	A	T	C	T	C	C	C	C	G	T	C	T	C	T	T	G	G	G	T	C	C	T	C	T	T	C	G	G	A	G	C	T	A	C	A	G	A	G	C	C	A	G	T	C	T	G	G	T	A	A	G	A	G	A	T	G	A	G	C	C	G	G	C	G	T	A	C	T	T	0	0	0	1	0	1
Hap_3	C	C	A	A	G	G	A	A	C	G	G	A	A	G	G	A	A	C	C	A	C	T	G	T	C	A	T	T	A	G	C	C	C	T	T	G	G	T	T	C	C	C	C	A	A	C	T	T	G	G	T	T	C	C	G	G	G	G	T	T	G	G	C	C	G	G	T	T	G	G	A	A	G	G	G	G	G	G	C	C	G	G	G	G	G	A	A	G	G	0	2	0	1	0	3
Hap_24	C	C	A	A	G	G	A	A	C	G	G	G	A	G	G	A	A	C	C	A	C	T	C	T	A	A	C	C	G	T	C	C	C	T	T	G	G	T	T	C	C	C	C	A	A	C	T	T	G	G	T	T	C	C	G	G	G	G	T	T	G	G	C	C	G	G	T	T	G	G	A	A	G	G	G	G	G	G	C	C	T	G	G	G	G	A	A	G	G	0	0	0	1	0	1
Hap_5	C	C	A	A	G	G	A	A	C	G	G	A	A	G	G	A	A	C	C	A	C	T	G	G	C	A	T	T	A	G	C	C	C	T	T	G	G	T	T	C	C	C	C	A	A	C	T	T	G	G	T	T	C	C	G	G	G	G	T	T	G	G	C	C	G	G	T	T	G	G	A	A	G	G	G	G	G	G	C	C	G	G	G	G	G	A	A	G	T	0	1	0	0	0	1
Hap_18	C	C	A	A	G	G	A	A	C	G	G	A	A	G	G	A	A	C	C	A	A	T	G	T	C	A	C	C	A	T	C	C	C	T	T	G	G	T	T	C	C	C	C	A	A	C	T	T	G	G	T	T	C	C	G	G	G	G	T	T	G	G	C	C	G	G	T	T	G	G	A	A	G	G	G	G	G	G	C	C	G	G	G	G	G	A	A	G	G	0	1	0	0	0	1
Hap_10	C	C	A	A	G	G	A	A	C	G	G	A	A	G	G	A	A	C	C	A	C	T	G	T	C	A	C	C	A	T	C	C	C	T	T	G	G	T	T	C	C	C	C	A	A	C	T	T	G	G	T	T	C	C	G	G	G	G	T	T	G	G	C	C	G	G	T	T	G	G	A	A	G	G	G	G	G	G	C	C	G	G	G	G	G	A	A	G	G	0	1	0	0	0	1
Hap_15	C	C	A	A	G	G	A	A	C	G	G	A	A	G	G	A	A	C	C	A	C	T	G	T	C	A	C	C	A	G	C	C	C	T	T	G	G	T	T	C	C	C	C	A	A	C	T	T	G	G	T	T	C	C	G	G	G	G	T	T	G	G	C	C	G	G	T	T	G	G	A	A	G	G	G	G	G	G	C	C	G	G	G	G	T	A	A	G	T	0	1	0	0	0	1
Hap_74	C	C	A	A	G	G	A	A	C	G	G	A	A	G	G	A	A	C	C	A	C	T	G	T	C	A	C	C	A	G	C	C	C	T	T	G	G	T	T	C	C	T	C	T	A	C	G	G	A	A	C	C	A	A	A	A	A	A	C	C	A	A	T	T	T	T	G	G	A	A	G	G	G	G	T	T	A	A	C	C	G	G	G	G	T	A	A	G	T	0	1	0	0	0	1
Hap_23	C	C	A	A	G	G	A	A	C	G	G	A	A	G	G	A	A	C	C	A	A	T	G	T	C	A	C	C	G	T	C	C	C	T	T	G	G	T	T	C	C	C	C	A	A	C	T	T	G	G	T	T	C	C	G	G	G	G	T	T	G	G	C	C	G	G	T	T	G	G	A	A	G	G	G	G	G	G	C	C	G	G	G	G	G	A	A	G	G	0	0	0	1	0	1
Hap_17	C	C	A	A	G	G	A	A	C	G	G	A	A	G	G	A	A	C	C	A	A	T	G	T	C	A	C	T	A	G	C	C	C	T	T	G	G	T	T	C	C	C	C	A	A	C	T	T	G	G	T	T	C	C	G	G	G	G	T	T	G	G	C	C	G	G	T	T	G	G	A	A	G	G	G	G	G	G	C	C	G	G	G	G	G	A	A	G	G	0	1	0	0	0	1
Hap_39	C	C	A	A	G	G	A	A	C	G	G	A	A	G	G	A	A	C	C	A	A	T	G	T	C	A	C	T	A	G	C	T	C	T	T	G	G	G	T	C	C	C	C	A	A	C	T	T	G	G	T	T	C	C	G	G	G	G	T	T	G	G	C	C	G	G	T	T	G	G	A	A	G	G	G	G	G	G	C	C	G	G	G	G	G	A	A	G	G	0	0	0	1	0	1
Hap_6	C	C	A	A	G	G	A	A	C	G	G	A	A	G	G	A	A	C	C	A	C	G	G	G	C	A	T	T	A	G	C	C	C	T	T	G	G	T	T	C	C	C	C	A	A	C	T	T	G	G	T	T	C	C	G	G	G	G	T	T	G	G	C	C	G	G	T	T	G	G	A	A	G	G	G	G	G	G	C	C	G	G	G	G	T	A	A	G	T	0	1	0	0	0	1
Hap_11	C	C	A	A	G	G	A	A	C	G	G	A	A	G	G	A	A	C	C	A	C	T	G	G	C	A	T	T	A	G	C	C	C	T	T	G	G	T	T	C	C	C	C	A	A	C	T	T	G	G	T	T	C	C	G	G	G	G	T	T	G	G	C	C	G	G	T	T	G	G	A	A	G	G	G	G	G	G	C	T	T	G	G	G	G	A	A	G	G	0	1	0	0	0	1
Hap_105	T	T	A	A	G	G	A	A	C	A	A	A	A	A	A	G	G	C	C	A	A	T	G	G	C	A	T	T	A	G	C	T	C	T	A	G	T	G	T	C	T	T	C	T	T	T	G	G	G	G	T	T	C	C	G	G	G	G	C	C	G	G	C	C	G	G	T	T	A	A	A	A	A	A	G	G	G	G	C	C	G	G	G	G	G	A	A	G	G	0	0	0	1	0	1
Hap_97	T	T	A	A	G	G	A	A	C	A	A	A	A	A	A	G	G	C	C	A	C	T	G	T	C	A	T	A	A	G	C	C	C	A	A	T	T	T	T	T	T	C	C	T	T	T	G	G	G	G	T	T	C	C	G	G	G	G	C	C	G	G	C	C	G	G	T	T	A	A	A	A	A	A	G	G	G	G	C	C	G	G	G	G	G	A	A	G	G	0	1	0	0	0	1
Hap_100	T	T	A	A	G	G	A	A	C	A	A	A	A	A	A	G	G	C	C	A	C	T	G	G	C	A	T	T	A	G	C	C	C	A	A	T	T	T	T	T	T	C	C	T	T	T	G	G	G	G	T	T	C	C	G	G	G	G	C	C	G	G	C	C	G	G	T	T	A	A	A	A	A	A	G	G	G	G	C	C	G	G	G	G	G	A	A	G	G	0	1	0	0	0	1
Hap_108	C	C	A	A	G	G	A	A	C	G	G	G	G	G	G	G	G	C	C	A	C	T	G	G	C	A	T	T	A	G	C	C	C	T	T	G	G	T	T	C	C	C	C	T	T	T	G	G	G	G	T	T	C	C	G	G	G	G	C	C	G	G	C	C	G	G	T	T	G	G	A	A	G	G	G	G	G	G	T	T	G	G	G	G	G	A	A	G	G	0	1	0	0	0	1
Hap_109	C	C	A	A	G	G	A	A	C	G	G	G	G	G	G	G	G	C	C	A	C	T	G	T	C	A	T	C	A	G	C	C	C	T	T	G	G	T	T	C	C	C	C	T	T	T	G	G	G	G	T	T	C	C	G	G	G	G	C	C	G	G	C	C	G	G	T	T	G	G	A	A	G	G	G	G	G	G	T	T	G	G	G	G	G	A	A	G	G	0	1	0	0	0	1
Hap_110	C	C	A	A	G	G	A	A	C	G	G	G	G	G	G	G	G	C	C	C	C	T	C	T	C	A	T	C	A	G	C	C	C	T	T	G	G	T	T	C	C	C	C	T	T	T	G	G	G	G	T	T	C	C	G	G	G	G	C	C	G	G	C	C	G	G	T	T	G	G	A	A	G	G	G	G	G	G	T	T	G	G	G	G	G	A	A	G	G	0	1	0	0	0	1
Hap_111	C	C	A	A	G	G	A	A	C	G	G	G	G	G	G	G	G	C	C	A	A	T	G	T	C	A	C	T	A	G	C	C	C	T	T	G	G	T	T	C	C	C	C	T	T	T	G	G	G	G	T	T	C	C	G	G	G	G	C	C	G	G	C	C	G	G	T	T	G	G	A	A	G	G	G	G	G	G	T	T	G	G	G	G	G	A	A	G	G	0	1	0	0	0	1
Hap_8	C	C	A	A	G	G	A	A	C	G	G	A	A	G	G	A	A	C	C	A	A	T	G	G	C	A	T	T	A	G	C	C	C	T	T	G	G	T	T	C	C	C	C	A	A	C	T	T	G	G	T	T	C	C	G	G	G	G	T	T	G	G	C	C	G	G	T	T	G	G	A	A	G	G	G	G	G	G	C	C	G	G	G	G	T	A	A	G	T	0	2	0	0	0	2
Hap_13	C	T	A	A	G	G	A	A	C	G	A	G	A	G	A	G	A	C	C	A	C	T	G	G	C	A	T	A	A	G	C	C	C	T	A	G	T	T	T	C	T	C	C	A	A	C	T	T	G	G	T	T	C	C	G	G	G	G	T	T	G	G	C	C	G	G	T	T	G	G	A	A	G	G	G	G	G	G	C	C	G	G	G	G	T	A	A	G	T	0	0	0	0	1	1
Hap_12	C	T	A	A	G	G	A	A	C	G	A	G	A	G	A	G	A	C	C	A	C	T	G	G	C	A	T	T	A	G	C	C	C	T	A	G	T	T	T	C	T	C	C	T	A	C	G	T	G	G	T	T	C	C	G	G	G	G	C	T	G	G	C	C	G	G	T	T	A	G	A	A	G	A	G	G	G	G	C	C	G	G	G	G	T	A	A	G	T	0	1	0	0	0	1
Hap_14	C	C	A	A	G	G	A	A	C	G	A	G	A	G	A	G	A	C	C	A	A	T	G	G	C	A	T	T	A	G	C	C	C	T	T	G	G	T	T	C	C	C	C	A	A	C	T	T	G	G	T	T	C	C	G	G	G	G	T	T	G	G	C	C	G	G	T	T	G	G	A	A	G	G	G	G	G	G	C	C	G	G	G	G	T	A	A	G	T	0	1	0	0	0	1
**Hap_45**	**C**	**C**	**C**	**C**	**A**	**A**	**C**	**C**	**G**	**G**	**G**	**G**	**G**	**G**	**G**	**G**	**G**	**T**	**T**	**A**	**A**	**T**	**C**	**T**	**C**	**C**	**C**	**C**	**G**	**T**	**T**	**T**	**T**	**T**	**T**	**G**	**G**	**G**	**G**	**C**	**C**	**T**	**T**	**T**	**T**	**C**	**G**	**G**	**A**	**A**	**C**	**C**	**A**	**A**	**A**	**A**	**A**	**A**	**C**	**C**	**A**	**A**	**T**	**T**	**T**	**T**	**G**	**G**	**A**	**A**	**G**	**G**	**G**	**G**	**T**	**T**	**A**	**A**	**C**	**C**	**G**	**G**	**G**	**G**	**G**	**A**	**A**	**G**	**G**	**2**	**11**	**2**	**2**	**1**	**18**
Hap_50	C	C	C	C	A	A	C	C	G	G	G	G	G	G	G	G	G	T	T	A	A	T	C	T	C	C	C	C	G	T	T	T	T	T	T	G	G	G	G	C	C	T	T	T	T	C	G	G	A	A	C	C	A	A	A	A	A	A	C	C	A	A	T	T	T	T	G	G	A	A	G	G	G	G	T	T	A	A	C	C	G	G	G	T	A	A	A	T	T	0	1	0	0	0	1
Hap_44	C	C	C	C	A	A	C	C	G	G	G	G	G	G	G	G	G	T	T	A	A	T	C	T	C	C	C	C	G	T	T	T	T	T	T	G	G	G	G	C	C	T	T	T	T	C	G	G	A	A	C	C	A	A	A	A	A	A	C	C	A	A	T	T	T	T	G	G	A	A	G	G	G	G	T	T	A	A	C	C	T	G	G	G	G	A	A	G	G	3	9	3	6	2	23
Hap_55	C	C	C	C	A	A	C	C	G	G	G	G	G	G	G	G	G	T	T	A	A	T	C	T	C	C	C	C	G	T	T	T	T	T	T	G	G	G	G	C	C	T	T	T	T	C	G	G	A	A	C	C	A	A	A	A	A	A	C	C	A	A	T	T	T	T	G	G	A	A	G	G	G	G	T	T	A	A	C	C	G	G	G	T	C	A	A	T	T	1	0	0	0	0	1
Hap_59	C	C	C	C	A	A	C	C	G	G	G	G	G	G	G	G	G	T	T	A	A	T	C	T	C	C	C	C	G	T	T	T	T	T	T	G	G	G	G	C	C	T	T	T	T	C	G	G	A	A	C	C	A	A	A	A	A	A	C	C	A	A	T	T	T	T	G	G	A	A	G	G	G	G	T	T	A	A	C	C	G	G	G	T	T	A	A	T	T	0	1	0	0	0	1
Hap_48	C	C	C	C	A	A	C	C	G	G	G	G	G	G	G	G	G	T	T	A	A	T	C	T	C	C	C	C	G	T	T	T	T	T	T	G	G	G	G	C	C	T	T	T	T	C	G	G	A	A	C	C	A	A	A	A	A	A	C	C	A	A	T	T	T	T	G	G	A	A	G	G	G	G	T	T	A	A	C	C	G	G	G	G	T	A	A	G	T	2	10	0	2	0	14
Hap_52	C	C	C	C	A	A	C	C	G	G	G	G	G	G	G	G	G	T	T	A	A	T	C	T	C	C	C	C	G	T	T	T	T	T	T	G	G	G	G	C	C	T	T	T	T	C	G	G	A	A	C	C	A	A	A	A	A	A	C	C	A	A	T	T	T	T	G	G	A	A	G	G	G	G	T	T	A	A	C	C	G	G	G	G	T	A	A	G	T	0	1	0	0	0	1
Hap_57	C	C	C	C	A	A	C	C	G	G	G	G	G	G	G	G	G	T	T	A	A	T	C	T	C	C	C	C	G	T	T	T	T	T	T	G	G	G	G	C	C	T	T	T	T	C	G	G	A	A	C	C	A	C	A	A	A	A	C	T	A	A	T	T	T	T	G	G	A	A	G	G	G	G	T	T	A	A	C	C	G	G	G	T	T	A	A	T	C	0	1	0	0	0	1
Hap_49	C	C	C	C	A	A	C	C	G	G	G	G	G	G	G	G	G	T	T	A	A	T	C	T	C	C	C	C	G	T	C	T	C	T	T	G	G	G	T	C	C	T	T	T	T	C	G	G	A	A	C	C	A	A	A	A	A	A	C	C	A	A	T	T	T	T	G	G	A	A	G	G	G	G	T	T	A	A	C	C	G	G	G	G	T	A	A	G	T	0	2	0	0	0	2
Hap_56	C	C	C	C	A	A	C	C	G	G	G	G	G	G	G	G	G	T	T	A	A	T	C	T	C	C	C	C	G	T	T	T	T	T	T	G	G	G	G	C	C	T	T	T	T	C	G	G	A	A	C	C	A	A	A	A	A	A	C	C	A	A	T	T	T	T	G	G	A	A	G	G	G	G	T	T	A	A	C	C	G	G	G	G	T	A	A	T	C	0	1	0	0	0	1
Hap_61	C	C	C	C	A	A	C	C	G	G	G	G	G	G	G	G	G	T	T	A	A	T	C	T	C	C	C	C	G	T	T	T	T	T	T	G	G	G	G	C	C	T	T	T	T	C	G	G	A	A	C	C	A	A	A	A	A	A	C	C	A	A	T	T	T	T	G	G	A	A	G	G	G	G	T	T	A	A	C	C	G	G	A	G	T	A	A	T	T	0	1	0	0	0	1
Hap_53	C	C	C	C	A	A	C	C	G	G	G	G	G	G	G	G	G	T	T	A	A	T	C	T	C	C	C	C	G	T	C	T	C	T	T	G	G	G	T	C	C	T	T	T	T	C	G	G	A	A	C	C	A	A	A	A	A	A	C	C	A	A	T	T	T	T	G	G	A	A	G	G	G	G	T	T	A	A	C	C	G	G	G	G	G	A	A	G	G	0	1	0	0	0	1
Hap_54	C	C	C	C	A	A	C	C	G	G	G	G	G	G	G	G	G	T	T	A	A	T	C	T	C	C	C	C	G	T	T	T	T	T	T	G	G	G	G	C	C	T	T	T	T	C	G	G	A	A	C	C	A	A	A	A	A	A	C	C	A	A	T	T	T	T	G	G	A	A	G	G	G	G	T	T	A	A	C	C	G	G	G	G	G	A	A	A	A	0	1	0	0	0	1
Hap_60	C	C	C	C	A	A	C	C	G	G	G	G	G	G	G	G	G	T	T	A	A	T	C	T	C	C	C	C	G	T	T	T	T	T	T	G	G	G	G	C	C	T	T	T	T	C	G	G	A	A	C	C	A	A	A	A	A	A	C	C	A	A	T	T	T	T	G	G	A	A	G	G	G	G	T	T	A	A	C	C	G	A	A	T	T	A	A	C	C	0	1	0	0	0	1
Hap_51	C	C	C	C	A	A	C	C	G	G	G	G	G	G	G	G	G	T	T	A	A	T	C	T	C	C	C	C	G	T	T	T	T	T	T	G	G	G	G	C	C	T	T	T	T	C	G	G	A	A	C	C	A	A	A	A	A	A	C	C	A	A	T	T	T	T	G	G	A	A	G	G	G	G	T	T	A	A	C	C	G	G	C	G	T	A	A	G	T	0	1	0	0	0	1
Hap_46	C	C	C	C	A	A	C	C	G	G	G	G	G	G	G	G	G	T	T	A	A	T	C	T	C	C	C	C	G	T	T	T	T	T	T	G	G	G	G	C	C	T	T	T	T	C	G	G	A	A	C	C	A	A	A	A	A	A	C	C	A	A	T	T	T	T	G	G	A	A	G	G	G	G	T	T	A	A	C	C	G	G	G	G	C	A	A	G	G	0	1	0	0	0	1
Hap_62	C	C	C	C	A	A	C	C	G	G	G	G	G	G	G	G	G	T	T	A	A	T	C	T	C	C	C	C	G	T	T	T	T	T	T	G	G	G	G	C	C	T	T	T	T	C	G	G	A	A	C	C	A	A	A	A	A	A	C	C	A	A	T	T	T	T	G	G	A	A	G	G	G	G	T	T	A	A	C	C	G	G	G	G	G	A	C	G	T	0	1	0	0	0	1
Hap_63	C	C	C	C	A	A	C	C	G	G	G	G	G	G	G	G	G	T	T	A	A	T	C	T	C	C	C	C	G	T	T	T	T	T	T	G	G	G	G	C	C	T	T	T	T	C	G	G	A	A	C	C	A	A	A	A	A	A	C	C	A	A	T	T	T	T	G	G	A	A	G	G	G	G	T	T	A	A	C	C	G	G	C	G	T	A	C	G	T	0	1	0	0	0	1
																																																																																											15	128	8	24	7	182

Supplementary Table S7 List of rice genotypes used in the study

S. No.	Variety	Group	Pedigree
1	ACHARMATHI	Aromatic	Local Selection
2	BADSHABHOG	Aromatic	Local Selection
3	BASMATI370	Aromatic	Local selection
4	BASMATI386	Aromatic	Local selection
5	HARYANABASMATI	Aromatic	Sona/Basmati370
6	IMPPUSABASMATI	Aromatic	Pusabasmati1/IRBB55
7	MAHISUGANDH	Aromatic	BK79/Basmati370,
8	PANTSUGANDH15	Aromatic	Basmati370/sadari/buharul/muskan41
9	PUSABASMATI1	Aromatic	Pusa-167Karnal Local
10	PUSASUGANDH3	Aromatic	IET16313/PUSA2504-1-31
11	PUSA1121	Aromatic	Pusa 614-1-2/Pusa 614-2-4-3
12	RANBIRBASMATI	Aromatic	Selection from Basmati370
13	SUGANDHASAMBHA	Aromatic	Early samba/RNR19994
14	TARAMATI	Aromatic	BPT5204/Tella Hamsa
15	TARAORIBASMATI	Aromatic	Pure line selection from HBC 19
16	TULASIJOHA	Aromatic	Rasi/Fine Gora
17	VASUMATHI	Aromatic	PR109/Pak Basmati
18	ABHAYA	Indica	CR 157-392/OR 57-21
19	ABHILASH	Indica	CR63-6218/Pankaj
20	ANANNDA	Indica	MTU-15/Yaikaku Nantoku
21	BADAMI	Indica	Shuphala/Annapurna
22	BAHADHUR	Indica	Pankaj/Mahsuri
23	BHADRAKALI	Indica	Phalguna/IR 36
24	BM71	Indica	Vajram/Bunnel//IR 64
25	BPT1204	Indica	Not Available
26	BPT1235	Indica	Sabarmati/W12708
27	BPT1768	Indica	BPT3301/Mahsuri
28	BPT2270	Indica	BPT5204/CR15 MR 1523
29	BPT2295	Indica	BPT 1768 x NLR 33641
30	BPT2231	Indica	BPT4358/IR64
31	BPT2411	Indica	BPT 5204 x BPT 4358
32	BPT2590	Indica	BPT 5204/MTU 1064//BPT 5204
33	BPT2613	Indica	MTU 7029/Moroberakan
34	BPT2673	Indica	BPT 5204/RP 4677-16-6-1-12-1-2
35	BPT2675	Indica	MTU 7029/MTU 4870/MTU 1081
36	BPT2678	Indica	MTU7029×Moroberaken
37	BPT4358	Indica	Sona Mahsuri/ARC 6650
38	BPT5204	Indica	GEB-24/ T(N)1 / Mahsuri
39	CAUVERY	Indica	TN1/TKM6
40	CR401	Indica	Vijaya/CR94-1512-6
41	DGWG	Indica	Mutant
42	DISANG	Indica	Lachit/Kalinga-3
43	DODIGA	Indica	Not Available
44	GANTESHWARI	Indica	IR-2061-628-1-6-4-3/N-2-2
45	GOVINDH	Indica	IR20/IR24
46	HIM2216	Indica	IR-8/IR-2053-521-1-1/IR-36
47	HIM2216-2	Indica	IR-8/IR-2053-521-1-1/IR-36
48	HIM799	Indica	IR28/Shensi var./IR28
49	HKR47	Indica	Not Available
50	IR1552	Indica	Not Available
51	IR29	Indica	IR833-6-2-1-1 IRI561-149-1/R24*4/o. Nivara

52	IR64	Indica	IR-5857-33-2-1/IR-2061-465-1-5-5
53	IR8	Indica	PETA/DGWG
54	JAGABHANDHU	Indica	Savitri / IR-4819 sel / IR 27301 sel
55	JAGANNADH	Indica	T-141 mutant
56	JAYA	Indica	T(N)1/T-141
57	JGL11470	Indica	JGL418/Gidongibeton
58	JGL17004	Indica	Not Available
59	JGL1798	Indica	Samba mahsury/Kavya
60	JGL3844	Indica	Not Available
61	JGL3855	Indica	Samba mahsury/ARC 5984/Kavya
62	KALINGA3	Indica	AC 540/Ratna
63	KANCHAN	Indica	Jagathi/Mahsuri
64	KANDHAGIRI	Indica	Parijat/IR-13429-94-3-2-2
65	KAPILEE	Indica	Heera x Annada
66	KAVYA	Indica	WGA27120/WGL17672/Mahsuri/Surekha
67	KESARI	Indica	Kumar/Jagannath
68	KONARK	Indica	Lalat x OR 135-3-4
69	KRISHNA	Indica	Chandan /BPT 5204
70	KRISHNA2	Indica	Chandan /BPT 5205
71	LALITHAGIRI	Indica	Badami/IR-1966-364
72	LEMONT	Indica	`Lebonnet'\ F1 of the cross CI9881/PI331581
73	LUIT	Indica	Heera x Annada
74	LN380	Indica	Mutant
75	MAHALAXMI	Indica	Pankaj/Mahsuri
76	MANDYA VIJAYA	Indica	Sona/Mahsuri
77	MANOHARSALI	Indica	Latisali/Guachari
78	MGD101	Indica	Teqing/Binam// Teqing///Teqing
79	MGD103	Indica	Not Available
80	MINGHUI63	Indica	Not Available
81	MRUNALINI	Indica	Mahalaxmi / OR 633 - 7
82	MSE9	Indica	Not Available
83	MTU1001	Indica	MTU 5249 x MTU 7014
84	MTU1010	Indica	Krishnaveni/IR-64
85	MTU1061	Indica	PLA1100/MTU1010
86	MTU1064	Indica	PLA 1100/MTU 1010
87	MTU1071	Indica	Recipient
88	MTU1075	Indica	MTU2716/MTU1010
89	MTU1121	Indica	BPT5204/MTU BB 8-24
90	MTU3626	Indica	IR8/MTU3
91	NDR359	Indica	BG-90-2-4 / 08677
92	NIDHI	Indica	Sona/ARC-14529
93	NILAGIRI	Indica	Suphala/DZ-192
94	NLR145	Indica	CICA-4/ IR-625-23-3-1 / Tetep (Swarnamukhi)
95	NLR3041	Indica	Not Available
96	NLR3042	Indica	Not Available
97	NLR30491	Indica	IR-36 x IR-2508 (Bharani (NLR-30491)
98	NLR3083	Indica	Not Available
99	NLR3217	Indica	Not Available
100	NLR3276	Indica	Not Available
101	NLR33358	Indica	Selection from IR-50
102	NLR33359	Indica	Selection from IR-50
103	NLR3354	Indica	Selection from IR-50
104	NLR33671	Indica	Not Available
105	NLR33892	Indica	NLR27999/MTU4870

106	NLR34342	Indica	Not Available
107	NLR34449	Indica	IR72/BPT5204
108	NLR4001	Indica	Not Available
109	NLR40024	Indica	IR50/CR14-8
110	NLR40054	Indica	MTU7029/RNR19994
111	NLR40058	Indica	Not Available
112	NLR40065	Indica	Not Available
113	P1144	Indica	Not Available
114	PANKAJ	Indica	Peta/Tongaki Rotan
115	PANKEMUTANT20	Indica	Not Available
116	PANTDHAN12	Indica	Govind/UPR 201
117	POTHANA	Indica	IR-579/WGL-12708
118	PR106	Indica	IR-8/ Peta 5/ Bella Patna
119	PR118	Indica	Not Available
120	PUSA44	Indica	IARI-5901-2/IR-8
121	RAJESWARI	Indica	T-90/IR-8
122	RAMAPPA	Indica	KAVYA/AC20
123	RANJIT	Indica	Pankaj / Mahsuri
124	RASI	Indica	TN1 / Co.29
125	RAVI	Indica	M-63-83×(RP-79-5×Rikutonorin-21)
126	RNR19186	Indica	BPT 5204 / Tella hamsa
127	SAKET4	Indica	TKM6/IR8
128	SATHYA	Indica	Tellahamsa/Rasi
129	SAVITRI	Indica	Pankaj/Jagannadh
130	SEETHAL	Indica	Not Available
131	SHOBHINI	Indica	Early Samba/ RNR 19994
132	SIDDHI	Indica	Not Available
133	SIRI1253	Indica	Not Available
134	SNEHA	Indica	Annada/CR 143-2-2
135	SONAMASOORI	Indica	Sona/Mahsuri
136	SONASALI	Indica	RP-1015-348-84-1/SONA/Manoharsali
137	SRAC34997	Indica	Not Available
138	SUPHALA	Indica	T-141 x TN-1
139	SURAKSHA	Indica	Sasyasree/MR-1523
140	SWARNA	Indica	Vasista / Mahsuri
141	SWARNASUB1A	Indica	Swarna/FR13A
142	TELLAHAMSHA	Indica	HR-12/T(N)1
143	TKM 6	Indica	GEB24/CO18
144	TRIGUNA	Indica	Swarnadhan/RP-1579-38
145	UDHAIYAGIRI	Indica	IRAT-138/IR-13543-66
146	UPAHAR	Indica	Mahalaxmi/IR62
147	VAJRAM	Indica	MTU4569/ARC6650
148	VANDHANA	Indica	C22 x Kalakeri
149	VARALU	Indica	Erramallelu/ CR 544-1-2
150	VASUNDHARA	Indica	Phalguna/IET 6858
151	VIVEKDHAN 62	Indica	China 4/BG367-4
152	VLDHAN 16	Indica	JP-5/YRL-1
153	VLDHAN65	Indica	Himalaya 2/ VL Dhan 16
154	WARANGALSAMBHA	Indica	BPT 5204/ARC5566/BPT3291
155	WGL11427	Indica	Not Available
156	WGL163	Indica	Not Available
157	WGL32100	Indica	Divya/BPT 5204
158	WGL347	Indica	Swarna Mukhi/kavya Warangal
159	WGL482	Indica	Not Available

160	WGL915	Indica	SN2R/IRPPN 39
161	KOSHIHIKARI	Japonica	Not Available
162	AC38460	Japonica	Not Available
163	AC38466	Japonica	Not Available
164	AC38534	Japonica	Not Available
165	AC41015	Japonica	Not Available
166	AC41038	Japonica	Not Available
167	AZUCENA	Japonica	Not Available
168	Nipponbare	Japonica	Not Available
169	DULAR	Landrace	Local selection
170	FR13A	Landrace	Local selection
171	AMO	Landrace	Local selection
172	CHANAOMJOYBANGLA	Landrace	Local selection
173	CHITTIMUTYALU	Landrace	Local selection
174	GANDHIBIROIN	Landrace	Local selection
175	HALYAMAMO	Landrace	Local selection
176	HASANSONA	Landrace	Local selection
177	INRC10192	Landrace	Local selection
178	INTAN	Landrace	Local selection
179	IYONGOMBATIAYONG	Landrace	Local selection
180	JALESHWARI	Landrace	Local selection
181	LALANAKANDA	Landrace	Local selection
182	MANJULI2	Landrace	Local selection
183	Nagina 22	Landrace	Selection from Rajbhog
184	POKKALI	Landrace	Local selection
185	PSB68	Landrace	Local selection
186	SABITA	Landrace	Pure line sel. from Boyan
187	SHARBATI	Landrace	Local selection from uttar pradesh
188	SOKBONGLONG	Landrace	Local selection
189	SOLUMPIKET	Landrace	Local selection
190	SONA	Landrace	Local selection
191	T309	Landrace	Local selection
192	TETEP	Landrace	Local selection
193	TYAAPIN	Landrace	Local selection
194	NL1	Wild derivatives	*O. glaberrima/O.sativa* derivative
195	NL3	Wild derivatives	*O. glaberrima/O.sativa* derivative
196	NL9	Wild derivatives	*O. glaberrima/O.sativa* derivative
197	WAB4502432P18HB	Wild derivatives	*O. glaberrima/O.sativa* derivative
198	RPBIO/248	Wild derivatives	Swarna/*O.nivara* (IRGC81848)
199	RPBIO/7K	Wild derivatives	KMR3/*O.rufipogan*
200	RPBIO/50-7	Wild derivatives	Swarna/*O.nivara*

Supplementary Table S8 The overlapping primer sequences used for sequencing of the targeted genes

**Gene**	**Primer**
Gn1a	
*Gn1a*_1F	cttcagggtgaacgagaagc
*Gn1a*_1R	agctgtggtgaccgagactt
*Gn1a*_2F	cctcgagggttcaaatgtgt
*Gn1a*_2R	cgtcggagaagaaggatgag
*Gn1a*_3F	gccaccttgtcccttctaca
*Gn1a*_3R	ccaagtccatgtcacaccag
*Gn1a*_4F	ctggtgtgacatggacttgg
*Gn1a*_4R	ggacgacattgagggagaaa
*Gn1a*_5F	caaagatctgtgcgccacta
*Gn1a*_5R	ccattgatcgatctccctgt
*Gn1a*_5R1	gatcgatctccctgtcaagc
*Gn1a*_6F	cgcacatatctgttgttctgc
*Gn1a*_6R	ccacatgtgtgtgtgacgtg
GS3	
*GS3*_1F	ccctacgacgacaaggtgtt
*GS3*_1R	gtgcatgatgctttcaccac
*GS3*_2F	gcgacacggactcttcgtta
*GS3*_2R	caaagttacaaaagttggacctca
Ghd7	
*Ghd7*_1F	cgtcagggactcaaaagagc
*Ghd7*_1R	aatttggcgactcctcattg
*Ghd7*_1R1	tcagcgagatcgatgacttg
*Ghd7*_2F	catacggatccagcctctgt
*Ghd7*_2R	ggcctgggtccattaatttt
*Ghd7*_3F	cacggactcacagagtgtgc
*Ghd7*_3R	aatgggccatcgatcactaa
*Ghd7*_4F	aaggaccaaatccatccaca
*Ghd7*_4R	acagaggctggatccgtatg
qSW5	
*qSW5*_1F	cgtgtggtaattccgatgtg
*qSW5*_1R	gcttaccaggaagcctctga
*qSW5*_2F	taatcatggcgtgtgttcgt
*qSW5*_2R	gctctcctcgtcgtctcact
*qSW5*_3F	tggcctcctctcctcttctt
*qSW5*_3R	gtacccgtacgacgacgact

sd1	
*sd1*_1F	GAAACTACTGCGAGCCCAAG
*sd1*_1R	GCCACCATGAGATTGGAGAT
*sd1*_7F	AAGCCCACAGACACAAAACC
*sd1*_7R	AGGCCACTCAGGTCAGTTGT
*sd1*_10F	TATTTTCCGATGGTGTGACG
*sd1*_10R	TCCCCACAAATTCCTTCAGA
*sd1*_4F	AGGAAATGCACTCCAACCAC
*sd1*_6R	ATGGCGGGTAGTAGTTGCAC

DEP1	
*DEP1*_3F	GGTGGATCGGGTATGTTTTG
*DEP1*_3R	CAGCAATTGGTAGAGCAC
*DEP1*_3F	GGTGGATCGGGTATGTTTTG
*DEP1*_3R	CTATTGGCGCTTCAATGGTT
*DEP1*_5F	GAACCTTTTGCCATTTTGGA
*DEP1*_5R	ACAATTGAGGCACCTTGGTC
*DEP1*_8F	GCGAGATCACGTTCCTCAAG
*DEP1*_8R	CTGAGTGACCATGCACCACAAT
*DEP1*_9F	ACCATGGTTTGAGGCTTTTG
*DEP1*_9R	GATTTGCTAAGGCCCTGTT
*DEP1*_10F	AGCAGTGGTGGTAGTAGTAGAAAG
*DEP1*_10R	AGAGGGAAGAGAGAGTTCAACAGC
*DEP1*_11F	ACGCAGCCATGATCCATCCCT
*DEP1*_11R	GAGCGAGCGAGTGTGAAAGAGA

Supplementary Table S9 The list of rice genotypes under each haplotype

Haplotype	Aromatic	Genotype(s)	Indica	Genotype(s)	Japonica	Genotype(s)	Landrace	Genotype(s)	Wild derivatives	Genotype(s)	Total
Ghd7-1	11	ACARMATHI, BASMATI370, BASMATI386, HARYANBASMATI, IPUSBASMAT, PANSUGAND15, PUSABASMATI, PUSASUGANDH3, S 130 RANBIRBASMATI, TARAMATI, VASUMATI	104	ABHAYA, ABHILASH, ANANNDA, BHADRAKALI, BM71, BPT1204, BPT1768, BPT2270, BPT2295, BPT2331, BPT2411, BPT2590, BPT2613, BPT2673, BPT2675, BPT2678, BPT4358, BPT5204, CAUVERY, CR401, DISANG, DODIGA, GANTESHWARI, GOVINDH, HIM2216, HIM799, HKR47, IR1552, IR29, IR64, IR8, JAGABHANDU, JAYA, JGL11470, JGL3855, KALINGA3, KANCHAN, KANDHAGIRI, KAPILEE, KESARI, KONARK, KRISHNA, LEOIT, LN380, MAHALAKSHMI, MINGHUI63, MRUNALINI, MSE9, MTU1001, MTU1010, MTU1061, MTU1071, MTU1075, MTU1121, NIDHI, NLR145, NLR30491, NLR33671, NLR34342, NLR3449, NLR40024, NLR40058, NLR40065, P1144, PANMUTANT20, PANTDHAN12, PR106, PR118, PUSA44, RAJESHWARI, RAMAPPA, RASI, RAVI003, RNR19186, SAKET4, SATHYA, SAVITHRI, SEETHAL, SIDDHI, SIRI1253, SONAMASOORI, SONASALI, SRAC34997, SUPHALA, SURAKSHA, SWARNA, SWARNASUB1A, TELLAHAMSA, TKM6, TRIGUNA, UPAHAR, VAJRAM, VANDHANA, VARALU, VASUNDHARA, VIVEKDHAN, VLDHAN16, VLDHAN66, WARASAMBHA, WGL163, WGL32100, WGL347, WGL482	8	AC38460, AC38466, AC38534, AC41015, AC41038, AZUCENA, KOSHIHIKARI, NBR16	18	AMO, CHITTIMUT, DULAR, GANDHIBIROIN, HALYAMAMO, HASANSONA, INRC10192, INTAN, IYONGOMBAT, JALESHWARI, LALANAKANDA, N22, PSB68, SHARBATI, SOKBONGLONG, SOLUMPIKET, SONA, T309, TYAAPIN	5	NL1, S 105 NL9, RPBIO248, RPBIO507, RPBIO7K	146
Ghd7-2	1	MAHISUGANDH	3	MANDYAVIJAYA, JGL 1798, JGL 3844							4
Ghd7-3			3	BAHADHUR, KAVYA, MTU 3626							3
Ghd7-4			3	JGL17004, LALITHAGIRI, RANJITH							3
Ghd7-5	1	PUSA1121									1
Ghd7-6							1	FR13A			1
Ghd7-7			1	BPT1235							1
Ghd7-8			2	PANKAJ, WGL 915							2
Ghd7-9			1	WGL11427							1
Ghd7-10			1	JAGANNADH							1
Ghd7-11			1	BADAMI			1	POKKALI			2
Ghd7-12			5	DGWG, NDR359, MANOHARSALI, MGD101, MGD103					1	WAB45024	6
Ghd7-13			1	SNEHA							1
Ghd7-14			1	NILAGIRI			1	CHANAOM			2
Ghd7-15	1	BADSHABHOG									1
Ghd7-16			1	UDHAIYAGIRI							1
Ghd7-17			1	LEMONT							1
Ghd7-18							1	TETEP			1
Ghd7-19	1	TARAORIBASMATI	1	POTHANA							2
Ghd7-20									1	NL3	1
Ghd7-21							1	SABITA			1
**Total**	**15**		**129**		**8**		**23**		**7**		**182**

Haplotype	Aromatic	Genotype(s)	Indica	Genotype(s)	Japonica	Genotype(s)	Landrace	Genotype(s)	Wild derivatives	Genotype(s)	Total
GS-1	1	TARAMATI									1
GS-2			3	KESARI, MTU 1061, VLDHAN66	1	AC41038	1	SOLUMPIKET			5
GS-3			2	BPT2411, MTU1071			1	POKKALI			3
GS-4			1	RAMAPPA							1
GS-5			1	SIDDHI							1
GS-6			1	JGL11470							1
GS-7			1	KONARK							1
GS-8			2	JGL1798, WGL347							2
GS-9					1	AC41015					1
GS-10			1	SONAMASOORI							1
GS-11			1	MTU1001							1
GS-12			1	NLR40058							1
GS-13									1	RPBIO7K	1
GS-14			1	NILAGIRI							1
GS-15			1	JGL3855							1
GS-16	1	ACARMATHI									1
GS-17			1	RASI							1
GS-18			1	BPT4358							1
GS-19			1	JAYA							1
GS-20							1	DULAR			1
GS-21			1	MANDYAVIJAYA							1
GS-22							1	INRC10192			1
GS-23							1	IYONGOMBAT			1
GS-24							1	JALESHWARI			1
GS-25			1	ABHAYA							1
GS-26			1	JAGANNADH							1
GS-27			1	ABHILASH							1
GS-28			1	WGL482							1
GS-29			1	BPT1768							1
GS-30			3	KAPILEE, MAHALAKSHMI, UPAHAR							3
GS-31							1	TYAAPIN			1
GS-32			1	BPT2331							1
GS-33			1	NLR40024							1
GS-34			4	BPT2675, JAGABHANDU, MSE9, VLDHAN16							4
GS-35			1	BM71							1
GS-36			1	BPT2270							1
GS-37			1	BPT2590							1
GS-38			1	LALITHAGIRI							1
GS-39							1	FR13A			1
GS-40			1	BPT5204							1
GS-41			1	BPT2295							1
GS-42			1	LEOIT							1
GS-43			1	CAUVERY							1
GS-44	3	PUSABASMATI, RANBIRBASMATI,TARAORIBASMATI	9	BPT2613, KALINGA3, BPT2678, MTU1075, DISANG, MINGHU163, TKM6, KRISHNA, ANANNDA	3	AC38466, AC38460, KOSHIHIKARI	6	SOKBONGLONG, GANDHIBIROIN, TETEP, LALANKANDA, T309, INTAN	2	NL9, NL1	23
GS-45	2	PANSUGAND15, PUSA1121	11	SRAC34997, BPT1235, PANKAJ, JGL17004, KAVYA, IR8, JGL3844, PR106, PR118, VANDHANA, NLR145	2	AZUCENA, NBR16	2	AMO, SHARBATI	1	NL3	18
GS-46			1	KANDHAGIRI							1
GS-47			1	HIM799							1
GS-48	2	IPUSBASMAT, HARYANABASMATI	10	NLR40065, RAVI003, SUPHALA, WGL915,PANTDHAN12,SAKET4,POTHANA,VAJRAM,RNR19186,SEETHAL			2	CHITTIMUT, PSB68			14
GS-49			2	BHADRAKALI, GANRESHWARI							2
GS-50			1	TELLAHAMSHA							1
GS-51			1	NLR3449							1
GS-52			1	SONASALI							1
GS-53			1	MTU1121							1
GS-54			1	IR64							1
GS-55	1	BASMATI370									1
GS-56			1	TRIGUNA							1
GS-57			1	WGL32100							1
GS-58			1	HIM2216							1
GS-59			1	WGL163							1
GS-60			1	MTU3626							1
GS-61			1	WGL11427							1
GS-62			1	RAJESWARI							1
GS-63			1	SATHYA							1
GS-64			1	PUSA44							1
GS-65	1	BASMATI386	1	VARALU							2
GS-66	1	MAHISUGANDH									1
GS-67			1	CR401							1
GS-68			1	SURAKSHA							1
GS-69							1	HASANSONA			1
GS-70			1	NIDHI							1
GS-71			1	UDHAIYAGIRI							1
GS-72			1	NLR33671							1
GS-73			1	SIRI1253							1
GS-74			1	WARSAMBHA							1
GS-75							1	SABITA			1
GS-76							1	CHANAOM			1
GS-77			1	VASUNDHARA							1
GS-78			2	BPT1204, LN380	1	AC38534					3
GS-79			1	LEMONT							1
GS-80			2	IR29, SAVITRI							2
GS-81	1	VASUMATHI									1
GS-82	1	PUSASUGANDH3	1	PANMUTANT20							2
GS-83									1	WAB45024	1
GS-84			1	GOVINDH							1
GS-85			1	HKR47							1
GS-86			1	P1144							1
GS-87			1	IR1552							1
GS-88			1	BAHADHUR							1
GS-89			1	DODIGA							1
GS-90			2	KANCHAN, SWARNA							2
GS-91			1	MRUNALINI							1
GS-92							1	HALYAMAMO			1
GS-93			1	SWARNASUB1A							1
GS-94			1	RANJIT							1
GS-95			1	SNEHA							1
GS-96									1	RPBIO507	1
GS-97			1	BPT2673							1
GS-98			1	NLR34342							1
GS-99			1	NLR30491							1
GS-100			1	VIVEKDHAN							1
GS-101									1	RPBIO248	1
GS-102	1	BADSHABHOG									1
GS-103			1	BADAMI							1
GS-104			1	MTU1010							1
GS-105							1	SONA			1
GS-106			1	DGWG							1
GS-107							1	N22			1
GS-108			1	MGD101							1
GS-109			1	MGD103							1
GS-110			1	NDR359							1
GS-111			1	MANOHARSALI							1
Total	15		128		8		24		7		182

Haplotype	Aromatic	Genotype(s)	Indica	Genotype(s)	Japonica	Genotype(s)	Landrace	Genotype(s)	Wild derivatives	Genotype(s)	Total
sd1-1	1	BADSHABHOG	3	LALITHAGIRI, SRAC34997, RANJIT			1	INTAN			5
sd1-2			2	ANANNDA, IR1552							2
sd1-3							1	CHANAOM			1
sd1-4			5	BHADRAKALI, LEOIT, MANDYAVIJAYA, BAHADHUR, SONASALI							5
sd1-5	10	ACARMATHI, HARYANBASMATI, IPUSBASMAT, MAHISUGANDH, PANSUGAND15 , PUSA1121, PUSABASMATI, PUSASUGANDH3, S 130 RANBIRBASMATI, TARAMATI	107	ABHAYA, ABHILASH, BADAMI, BPT1235, BPT1768, BPT2270, BPT2295, BPT2331, BPT2411, BPT2590, BPT2613, BPT2673, BPT2675, BPT2678, BPT4358, BPT5204, CAUVERY, CR401, DGWG, DISANG, DODIGA, GANTESHWARI, GOVINDH, HIM2216, HIM799, HKR47, IR29, IR8, JAGABHANDU, JAYA, JGL 17004, JGL 1798, JGL3844, JGL3855, KANCHAN, KANDHAGIRI, KAPILEE, KAVYA, KESARI, KONARK, KRISHNA, LN380, MAHALAKSHMI, MGD101, MGD103, MINGHUI63, MRUNALINI, MSE9, MTU1001, MTU1010, MTU1061, MTU1075, MTU1121, MTU3626, NDR359, NIDHI, NILAGIRI, NLR145, NLR30491, NLR33671, NLR34342, NLR3449, NLR40024, NLR40058, NLR40065, P1144, PANKAJ, PANMUTANT20, PANTDHAN12, POTHANA, PR106, PR118, PUSA44, RAMAPPA, RASI, RAVI003, RNR19186, SAKET4, SATHYA, SAVITHRI, SEETHAL, SIDDHI, SIRI1253, SNEHA, SONAMASOORI, SUPHALA, SURAKSHA, SWARNA, SWARNASUB1A, TELLAHAMSA, TKM6, TRIGUNA, UDHAIYAGIRI, UPAHAR, VAJRAM, VARALU, VASUNDHARA, VIVEKDHAN, VLDHAN16, VLDHAN66, WARASAMBHA, WGL11427, WGL163, WGL32100, WGL347, WGL482, WGL915	7	AC38460, AC38466, AC38534, AC41038, AZUCENA, KOSHIHIKARI, NBR16	16	AMO, CHITTIMUT, DULAR FR13A, GANDHIBIROIN, INRC10192, IYONGOMBAT, JALESHWARI, LALANAKANDA, N22, POKKALI, PSB68, SABITA, SHARBATI, SOKBONGLONG, SONA	7	NL1, S 104 NL3, S 105 NL9, RPBIO248, RPBIO507, RPBIO7K, WAB45024	147
sd1-6	1	BASMATI386					1	TYAAPIN			2
sd1-7			1	RAJESWARI							1
sd1-8					1	AC41015					1
sd1-9			1	IR64							1
sd1-10			1	JAGANNADH							1
sd1-11							1	HALYAMAMO			1
sd1-12			1	BM71			1	SOLUMPIKET			2
sd1-13	1	VASUMATHI					2	HASANSONA, T 309			3
sd1-14			1	LEMONT							1
sd1-15			1	BPT1204							1
sd1-16	2	BASMATI370, TARAORIBASMATI	4	MTU1071, VANDANA, MANOHARSALI, KALINGA3			1	TETEP			7
sd1-17			1	JGL11470							1
Total	15		128		8		24		7		182

Haplotype	Aromatic	Genotype (s)	Indica	Genotype (s)	Japonica	Genotype (s)	Land race	Genotype (s)	Wild derivatives	Genotype (s)	Total
Gn1a-1	5	ACARMATHI, PUSA1121, S 130 RANBIRBASMATI, TARAMATI, TARAORIBASMATI	14	JGL11470, NLR40058, NLR3449, BADAMI, BPT1204, GOVINDH, BHADRAKALI, MTU1001, KANCHAN, NLR33671, IR29, BPT5204, VIVEKDHAN, WGL11427	1	AC38460	8	CHITTIMUT, DULAR FR13A, IYONGOMBAT, WGL11427, N22, SOLUMPIKET, SONA, TYAAPIN	2	NL3,NL9	30
Gn1a-2	6	BADSHABHOG, BASMATI370, BASMATI386, HARYANBASMATI, IPUSBASMAT, PANSUGAND15, PUSABASMATI, PUSASUGANDH3	64	ABHILASH, BPT1235, BPT2590, BPT2673, BPT2675, BPT2678, BPT4358, CAUVERY, DODIGA, GANTESHWARI, HKR47, JAGABHANDU, JAYA, JGL 17004, JGL3844, JGL3855, KALINGA3, KANDHAGIRI, KAVYA, KESARI, LALITHAGIRI, LN380, MAHALAKSHMI, MRUNALINI, MSE9, MTU1010, MTU1071, MTU1121, NDR359, NILAGIRI, NLR30491, NLR34342, NLR40024, NLR40065, PANMUTANT20, POTHANA, PR106, RAJESHWARI, RAMAPPA, RANJIT, RASI, RAVI003, RNR19186, SATHYA, SEETHAL, SIDDHI, SIRI1253, SONASALI, SRAC34997, SURAKSHA, SWARNA, SWARNASUB1A, TELLAHAMSA, TKM6, TRIGUNA, UPAHAR, VANDHANA, VARALU, VASUNDHARA, VLDHAN66, WARASAMBHA, WGL163, WGL347, WGL482, WGL915	3	AC41015, AC41038, NBR16	8	GANDHIBIROIN, INRC10192, INTAN, JALESHWARI, POKKALI, SABITA, SHARBATI, FR13A	4	RPBIO2 48, RPBIO5 07, RPBIO7 K, WAB450 24	85
Gn1a-3			4	HIM799, IR 1552, NIDHI, SAVITRI							4
Gn1a-4			1	IR8							1
Gn1a-5			10	MANDYAVIJAYA, MANOHARSALI, MGD101, MGD103, BAHADUR, BPT2295, BPT2331, JGL1798, SONAMASOORI, WGL32100	1	AC38466	2	SOKBONGLONG, T309			13
Gn1a-6			1	ANANNDA							1
Gn1a-7							1	CHANAOM			1
Gn1a-8			1	BPT1768							1
Gn1a-9			5	BM71, DGWG, DISANG, KAPILEE, MRUNALINI			1	TETEP			6
Gn1a-10	2	MAHISUGANDH, PAN SUGAND 15	2	PR118, SNEHA			2	AMO, PSB 68			6
Gn1a-11			1	SUPHALA							1
Gn1a-12			2	KRISHNA, PANKAJ							2
Gn1a-13			1	KONARK							1
Gn1a-14			1	MTU1075							1
Gn1a-15	1	VASUMATHI	9	MINGHUI63, MTU 1061, ABHAYA, IR64, SAKET4, VLDHAN16, HIM2216, CR401, VAJRAM	1	AZUCENA					11
Gn1a-16			2	NLR145, PANTDHAN12							2
Gn1a-17							1	HASANSONA			1
Gn1a-18					1	KOSHIHIKARI					1
Gn1a-19							1	HALYAMAMO			1
Gn1a-20			2	BPT2411, BPT 2613	1	AC38534			1	NL1	4
Gn1a-21			4	JAGANNADH, MTU 3626, PUSA44, UDHAIYAGIRI							4
Gn1a-22			1	LEOIT							1
Gn1a-23			1	BPT2270							1
Gn1a-24			2	LEMONT, P1144							2
Gn1a-25	1	BASMATI370									1
Total	15		128		8		24		7		182

Haplotype	Aromatic	Genotype(s)	Indica	Genotype(s)	Japonica	Genotype(s)	Landrace	Genotype(s)	Wild derivatives	Genotype(s)	Total
Qsw5-1	2	BASMATI370, PUSASUGANDH3	3	GANTESHWARI, LN380, SURAKSHA			1	LALANAKANDA			6
Qsw5-2	1	BADSHABHOG	1	MANOHARSALI	1	AC41015					3
Qsw5-3			1	MGD101							1
Qsw5-4			7	BADAMI, VLDHAN16, DGWG, MRUNALINI, UPAHAR, JAGANNADH, IR8	1	AC41038	4	AMO, POKKALI, SABITA, SOLUMPIKET			12
Qsw5-5			1	VLDHAN66							1
Qsw5-6			9	GOVINDH, JGL 11470, SUPHALA, NLR34342, BHADRAKALI, NLR145, NLR40065, PANMUTANT20, KONARK							9
Qsw5-7			1	JAGABHANDHU							1
Qsw5-8			2	MSE9, NDR359							2
Qsw5-9							1	DULAR			1
Qsw5-10	8	ACARMATHI, BASMATI386, HARYANBASMATI, MAHISUGANDH, PANSUGAND15, PUSABASMATI, TARAMATI, VASUMATI	95	ABHAYA, ABHILASH, ANANNDA, BM71, BPT1204, BPT1235, BPT2270, BPT2295, BPT2331, BPT2411, BPT2590, BPT2613, BPT2673, BPT2675, BPT2678, BPT4358, BPT5204, CAUVERY, CR401, DISANG, DODIGA, HIM2216, HKR47, IR1552, IR29, IR64, JGL 17004, JGL 1798, JGL3844, JGL3855, KALINGA3, KANCHAN, KANDHAGIRI, KAPILEE, KAVYA, KESARI, KRISHNA, LALITHAGIRI, LEMONT, LEOIT, MAHALAKSHMI, MANDYAVIJAYA, MGD103, MINGHUI63, MTU1001, MTU1010, MTU1061, MTU1071, MTU1075, MTU1121, MTU3626, NIDHI, NILAGIRI, NLR30491, NLR33671, NLR3449, NLR40024, NLR40058, P1144, PANTDHAN12, PR106, PR118, PUSA44, RAJESHWARI, RAMAPPA, RANJIT, RASI, RAVI003, SAKET4, SATHYA, SAVITHRI, SEETHAL, SIDDHI, SIRI1253, SONAMASOORI, SONASALI, SRAC34997, SWARNA, SWARNASUB1A, TELLAHAMSA, TKM6, TRIGUNA, UDHAIYAGIRI, VAJRAM, VANDHANA, VARALU, VASUNDHARA, VIVEKDHAN, WARASAMBHA, WGL11427, WGL163, WGL32100, WGL347, WGL482, WGL915	6	AC38460, AC38466, AC38534, AZUCENA, KOSHIHIKARI, NBR16	13	CHITTIMUT, FR13A, GANDHIBIROIN, HALYAMAMO, INRC10192, INTAN, IYONGOMBAT, JALESHWARI, N22, PSB68, SOKBONGLONG, SONA, TYAAPIN	7	NL1, S 104 NL3, S 105 NL9, RPBIO248, RPBIO507, RPBIO7K, WAB45024	129
Qsw5-11			1	SNEHA							1
Qsw5-12			1	BPT1768							1
Qsw5-13			1	JAYA							1
Qsw5-14	1	IPUSBASMAT	2	POTHANA, RNR19186			1	TETEP			4
Qsw5-15	1	RANBIRBASMATI					1	T309			2
Qsw5-16			2	BAHADHUR, HIM799			1	HASANSONA			3
Qsw5-17	1	TARAORIBASMATI	1	PANKAJ							2
Qsw5-18							1	CHANAOM			1
Qsw5-19	1	PUSA1121					1	SHARBATI			2
Total	15		128		8		24		7		182
